# Overcoming polymorphism: a revised list of shell characters for the phylogenetic analysis of soft-shelled turtles (*Pan-Trionychidae*)

**DOI:** 10.1186/s13358-025-00360-x

**Published:** 2025-05-30

**Authors:** Walter G. Joyce

**Affiliations:** Department of Geosciences, University of Fribourg, 1700 Fribourg, Switzerland

**Keywords:** *Testudines*, *Trionychia*, *Trionychidae*, Character ordering, Outgroup, Character performance

## Abstract

Soft-shelled turtles (*Pan-Trionychidae*) are one of the primary clades of turtles with a particularly rich fossil record reaching back to the Early Cretaceous. Yet, the evolution of the group has been difficult to resolve, in part because the fossil record mostly consists of shells and because the shells are known to exhibit high levels of polymorphism, making it difficult to establish parsimony-informative characters. A revision of the shell osteology of extant and extinct pan-trionychid turtles resulted in the development of 69 revised and novel characters with over 221 derived character states. Of these, 40 are multistate characters and 11 morphometric characters that utilize length, surface, and angular measurements. In a first step, the characters were scored for 530 regularly developed individuals representing all currently recognized species of living trionychids. The primary dataset confirms that most characters are affected by high levels of polymorphism. Statistical analyses conclude that much variability can be attributed to ontogenetic changes. In a second step, the primary data was used to code terminal taxa by reference to the most adult individuals for characters controlled by ontogeny. Terminals were otherwise only scored polymorphic if at least 20% of individuals displayed a particular character state. A phylogenetic analysis concludes that the new characters converge best upon the emerging molecular consensus, if characters are run ordered. All three utilized outgroups have a negative impact on ingroup relationships and character evolution, which can only partially be addressed through the use of a molecular backbone. The reduction of polymorphism by reference to adults and a minimum frequency of 20% yields more parsimony-informative characters and character states. A brief account is provided on how to diagnose all extant clades and species of trionychids using osteological characters.

**Supplementary Information:**

The online version contains supplementary material available at 10.1186/s13358-025-00360-x.

## Introduction

**Fig. 1:**
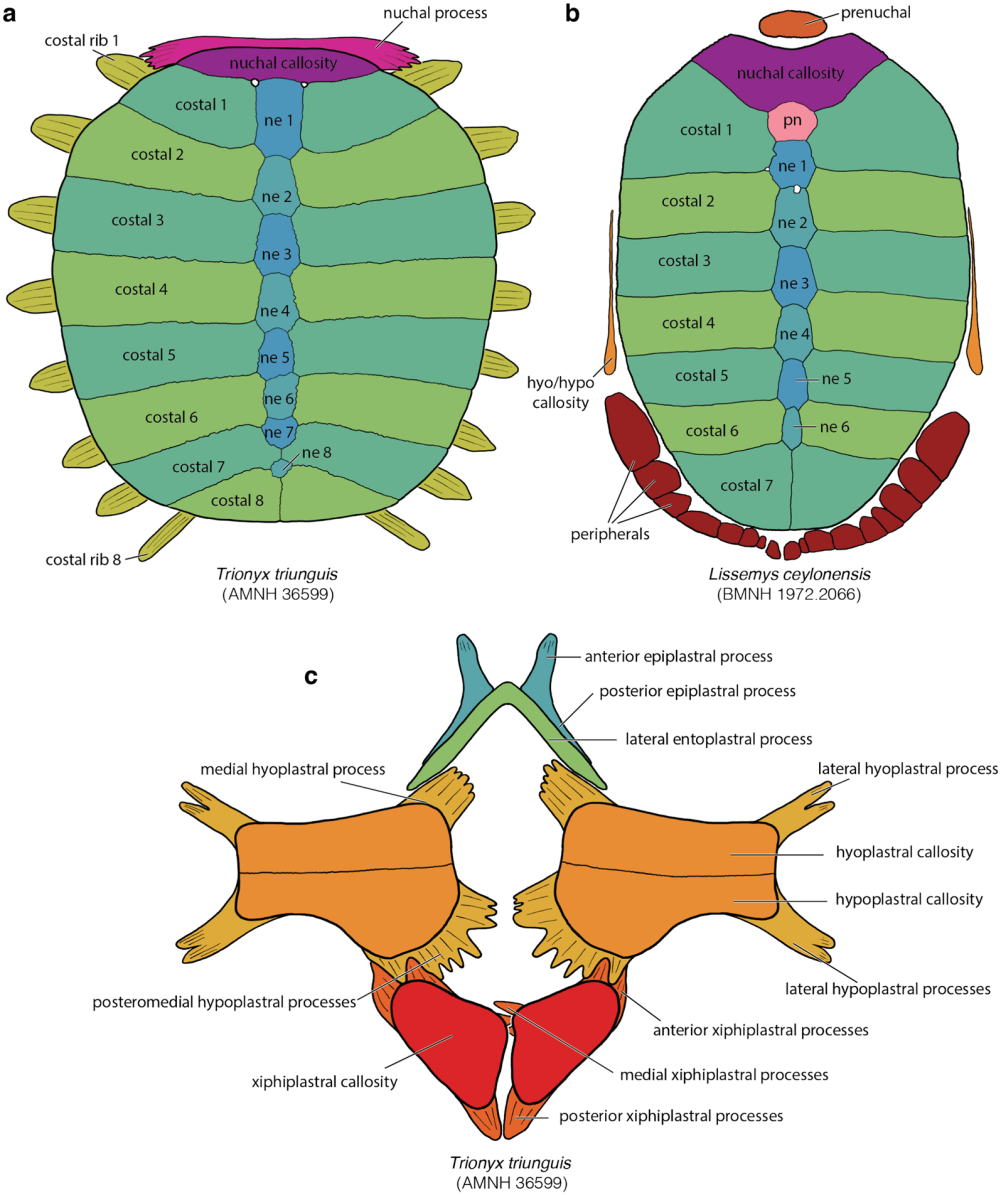
A summary of the morphological shell nomenclature of pan-trionychids used herein. Line drawings of carapace in dorsal view: **a** *Trionyx triunguis* (AMNH 36599), **b** *Lissemys ceylonensis* (BMNH 1972.2066), **c** *Trionyx triunguis* (AMNH 36599). Images are not to scale. ne, neural; pn, preneural; hyo, hypoplastron; hypo, hypoplastron

Soft-shelled turtles (*Trionychidae*) are one of the primary crown groups of extant turtles (*Testudines*). They are easily recognizable externally, as the shell is greatly flattened and covered by a thickened skin, not keratinous scales, allowing these animals to bury themselves for extended times in soft sediments to ambush their prey while respiring over the surface of their body (Bagatto & Henry, 1999; Ernst & Barbour, 1989; Pritchard, 1979; Ultsch, et al., 1984). The shell of trionychids is highly apomorphic relative to other turtles: the peripherals and pygals are lost and the epi- and entoplastra rebuilt into boomerang-shaped elements (Meylan, 1987). In addition, an unusually strong distinction is developed between the underlying “endoskeletal” portions of the shell (e.g., dermal portions of the shoulder girdle, ribs, gastralia; Lyson et al., 2013; Zangerl, 1939) and the overlying, metaplastically ossified dermis (Scheyer et al., 2007). This distinction is particularly pronounced in the nuchal and plastron, where convention dictates that the strap-like deep tissue portions are referred to as “processes,” while the textured dermal portions are referred to as “callosities” (Meylan, 1987; Fig. 1).

The history of the total group of soft-shelled turtles (*Pan-Trionychidae*) appears to be relatively well documented by a rich fossil record that extends back to the Early Cretaceous and includes all major landmasses but Antarctica (Georgalis & Joyce, 2017; Vitek & Joyce, 2015). Yet, the evolution of the group remains somewhat shrouded in mystery, as the phylogenetic relationships of most fossil forms remain unclear, thus obscuring the investigation of adaptive patterns, biogeography, or evolutionary rates. These difficulties are not only a result of the fragmentary nature of the trionychid fossil record, mostly shells, but also particularly high levels of polymorphism (Brinkman et al., 2017; Meylan, 1987), which is the cumulative result of ontogenetic changes, phenotypic variation, minor pathologies, and sexual dimorphism (see below) and which complicates the development of informative phylogenetic characters.

The first to address the phylogenetic relationships of trionychid turtles in earnest was Meylan (1987), who developed 59 informative osteological characters, of which 24 are multistate, 9 utilize polymorphic character states, 4 ontogenetic characteristics, and 5 morphometric. While more recent molecular data (e.g., Engstrom et al., 2004; Le et al., 2014; Thomson et al., 2021) has all but obviated the need for morphological characters that resolve the phylogenetic relationships of extant trionychids, Meylan’s (1987) character/taxon matrix has gained increasing importance over the years for paleontologists, who have utilized it to assess the phylogenetic relationships of numerous extinct pan-trionychids, not only by including fossil taxa, but also by adding new characters that address newly discovered morphologies (Brinkman et al., 2017; Cadena, 2016; Danilov et al., 2014; Edgar et al., 2022; Evers et al., 2023; Gardner et al., 1995; Girard et al., 2024; Jasinski et al., 2022; Joyce & Lyson, 2010, 2011, 2017; Joyce et al., 2009, 2016, 2018; Li et al., 2015; Lyson et al., 2021; Massonne et al., 2023; Valdes et al., 2017; Vitek, 2011, 2012; Vitek et al., 2018). Yet, given that most fossil pan-trionychid taxa are known from shells only, it is increasingly clear that the fossil record demands a revised set of shell characters that integrates new observations from the fossil record while rigorously addressing issues associated with polymorphism.

The purpose of this study is to establish an expanded set of shell characters of extant trionychids using best practices for the purpose of better resolving the evolutionary history of the group using fossils. As the phylogeny of recent trionychids is well resolved using molecular data (Engstrom et al., 2004; Le et al., 2014; Thomson et al., 2021) the accuracy of the new characters will be assessed by reference to a molecular consensus tree. In contrast to previous analyses, all raw observational data at the level of the individual is gathered and reported separately from the final coding of all involved species. This has the advantage of providing the community with the opportunity to include additional specimens to the matrix while exploring alternative coding strategies. An equally needed, expanded set of non-shell characters will be provided in a separate contribution.

## Materials and methods

### Sampling

Trionychids exhibit high levels of polymorphism associated with growth (i.e., juveniles have different morphotypes than adults), sexual dimorphism, and phenotypic plasticity (Meylan, 1987). As single individuals are rarely representative of their species, this study attempts to document the morphology of each taxon by sampling as many subadult to adult individuals as possible. This is not a trivial task as no single museum collection is available that densely samples all extant species of trionychids, a situation which is certainly not unique to the group (see Garbin et al., 2018 for *Geoemydidae*). A significant sample can nevertheless be realized through travel or the use of digital media, in particular photographs. Either approach has its merits and pitfalls. Assuming that good lighting and microscopes are available, studying museum specimens in person has the advantage of allowing rigorous assessment of all externally visible character systems and acquisition of direct measurements for morphometric characters, but observations cannot be rechecked at a later date without renewed access to the same specimens. The use of photographs, on the other hand, allows continuously revising character definitions and scoring characters consistently, but measurements may be distorted and some characters might not be observable due to poor lighting or focus in the available pictures. These problems are somewhat diminished when working with trionychid shells, as osteological specimens are typically preserved in partial to full disarticulation, thus allowing for their documentation in straight views. In addition, most elements are relatively flat, thereby minimizing distortion. As visiting museums to obtain a sufficient dataset was not feasible within the confines of this study, I opted for using digital photographs of museum specimens as the primary source of character information. The most important downside of the approach, the distortion of measurements, was addressed by developing characters less affected by this source of error and by incorporating measurement uncertainty into the coding procedures (see List of Characters below).

The set of photographs utilized herein is a collaborative effort of the fossil turtle community (see Acknowledgements). As a result, the photographs I utilized are uneven in their qualities as they were taken over the course of the last four decades by different people using different equipment and with different goals in mind. Many characters are consequently scored unknown for particular specimens, because the character in question is not visible in the available photographs or because the relevant anatomical system was not photographed at all. This loss of information is partially compensated, once again, through the large sample size.

The initial sample of photographs of more than 700 osteological specimens was surveyed for inclusion in this study. Specimens were excluded if they did not include shell material, if they were greatly damaged or highly abnormal (e.g., kyphotic individuals such as *Amyda cartilaginea* PCHP 4930, *Apalone ferox* USNM 73199, or *Trionyx triunguis* SMF 32812), or if they were too incomplete or too juvenile (e.g., lacking shell callosities). Specimens with uncertain taxonomic affinities were disregarded as well (see Taxonomy below). The final sample consists of 530 individuals representing all currently recognized species of extant trionychids, with exception of *Pelodiscus* species, which were grouped into a single taxon (see Taxonomy below). Six species stand out by having poor sampling of five individuals or less, namely *Chitra vandijki* (N = 1), *Nilssonia leithii* (N = 2), *Nilssonia nigricans* (N = 4), *Palea steindachneri* (N = 3), *Pelochelys signifera* (N = 1), and *Rafetus swinhoei* (N = 5). *Amyda ornata* stands out by having a particularly large sample (N = 102), which is three times greater than the next best sampled species. On average, each taxon is represented by about 18 individuals. The final list of specimens is provided in Additional file 1. The full set of photographs can be downloaded from Dryad (10.5061/dryad.jq2bvq8m2).

### Alpha taxonomy

The taxonomy of extant trionychids has undergone numerous changes over the course of the last two centuries (e.g., Boulenger, 1889; Duméril & Bibron, 1834; Gray, 1831; Meylan, 1987; Siebenrock, 1909; TTWG, 2021; Wermuth & Mertens, 1961) and it is therefore to be expected that many museum specimens are labelled or cataloged using outdated names and/or incorrect identifications. I, therefore, verified the taxonomic identity of all specimens prior to further analysis using the taxonomy of TTWG (2021) by recognizing 34 species of extant trionychids.

In a first pass, I updated all disused taxonomic names by reference to TTWG (2021), who exhaustively list the junior synonyms of all extant taxa they recognize.

A number of taxa consistently recognized as species over the course of the last decades were recently split into two or more species on molecular grounds, including what was formerly *Amyda cartilaginea* (now two species), *Chitra chitra* (now three species), *Lissemys punctata* (now three species), *Pelochelys bibroni* (now three species), and *Pelodiscus sinensis* (now six species) (see TTWG, 2021 for most recent summary). While the relevant studies typically list external characteristics that allow diagnosing the new species they recognize, osteological characters are not listed (Farkas et al., 2019; McCord & Pritchard, 2002; Webb, 2002). As all currently recognized species originate from distinct regions of the world, I reidentified in a second round all individuals of the affected species complexes by reference to their locality data and maps published by TTWG (2021). As a result, some wild caught specimens with good locality data needed to be excluded from the analysis, as they originate from geographic areas with uncertain taxonomic attribution, such as *Amyda* specimens from the Malay Peninsula (TTWG, 2021; e.g., *Amyda cartilaginea* FMNH 224117 and FMNH 224223). In total, the taxonomic attribution of more than 100 individuals was updated relative to their museum labels in this step.

In a third and final step, the species attribution of all specimens was reaffirmed by reference to their anatomy. This step was somewhat complicated by the fact that no synthesis exists on the osteology of extant trionychids beyond the character state matrix of Meylan (1987) and that high level of previously reported polymorphism preclude exclusive use of key characters (Meylan, 1987). In a heuristic process, pictures of all specimens initially attributed to a particular taxon were morphologically compared to one another and the scorings of Meylan (1987) in a search for morphological outliers (e.g., NMB 29978, a specimen identified as *Trionyx triunguis* on the label, but exhibiting split lateral nuchal processes, which is inconsistent with this taxon). The morphological outliers were then compared to other taxa in search of more consistent attributions (e.g., NMB 29989 exhibits split lateral nuchal processes, a preneural, a complete neural column, a posteriorly constricted carapacial outline, and enlarged eighth costals, which is consistent with *Cycloderma frenatum*). The identity of specimens known to not be collected from the wild (e.g., zoo and pet trade specimens) was scrutinized with particular care, as their identity was often revealed to be incorrect and because they lack locality data that might reaffirm a particular identification. However, as the source of most specimens is not apparent from museum labels or data bases, it was not possible to clarify as part of this study if the inclusion of zoo and pet trade specimens introduces a systematic bias. The specific identity of numerous specimens attributed to taxa recently split into multiple species based on molecular grounds (see above) could not be clarified beyond the generic level if locality data was lacking, as the relevant literature does not highlight morphological differences and because specimens with unambiguous locality data did not reveal characters that would help the identification of specimens that lack locality data. The exception to the rule are the three currently recognized species of *Lissemys*, which can be distinguished with relative ease by reference to the size and attachment of their prenuchal and the development of the plastral callosities. All in all, this third step led to the complete reidentification of about 20 individuals and omission of approximately 50 others, mostly juvenile animals from the pet trade. The most important reidentification is perhaps PCHP 2746, which was reported by Pritchard (2001) as the largest known shell of *Apalone mutica*, but actually represents a relatively regularly sized female *Apalone spinifera*.

The *Pelodiscus* species complex remains morphologically intractable for the moment for multiple reasons. Until relatively recently (e.g., Ernst & Barbour, 1989), all six currently recognized species were thought to form a single species, *Pelodiscus sinensis*, that today occurs along the entire eastern cost of temperate to tropical Asia and the nearby Islands of Hainan, Taiwan, and Japan (TTWG, 2021). Indeed, all osteological specimens included in this study are cataloged under that name. Members of the *Pelodiscus* species complex have been farmed for hundreds of years across East Asia, often represent hybrids, and were readily moved across Asia for trade (e.g., Fritz et al., 2010; Farkas et al., 2019; Gong et al., 2018, 2022; Stuckas & Fritz, 2011; Suzuki et al., 2014; Zhang et al., 2017;). So, while many of the available specimens are associated with locality data, it is not possible to ascertain their taxonomic identity by reference to their locality, as it is typically unclear if the individual is a true native collected from the wild or a farmed animal. Given the deep temporal divergence of the *Pelodiscus* group (Le et al., 2014), morphologically differentiating all six currently recognized species is a worthwhile goal, also as a way to phylogenetically place fossil representatives of the group (Chow & Yeh, 1958; Georgalis & Joyce, 2017; Yeh, 1963). This could be accomplished by gathering morphological data from specimens with known provenance and genetic affinities, for instance by CT scanning wet specimens preserved in alcohol. However, as this is far beyond the scope of this study, all *Pelodiscus* specimens are herein united into a single terminal taxon, *Pelodiscus* sp.

The oldest pan-trionychid shell preserved in dorsal and ventral view belongs to the holotype of *Perochelys lamadongensis* from the Early Cretaceous of China (Li et al., 2015). This fossil was added to the matrix for potential use as an outgroup.

The final identification of all utilized specimens is provided in the character-taxon matrix (see Additional file 1).

### Anatomical nomenclature

The shell of turtles in general, and of trionychid in particular, is universally agreed to be a tightly intermingled composite formed by numerous “endoskeletal” elements (i.e., dorsal vertebrae and ribs, cleithra, interclavicle, clavicles, and gastralia) and the metaplastically ossified dermis (e.g., Lyson et al., 2013; Scheyer 2009; Zangerl, 1939). Among extant turtles, the shell of trionychids is unusual, as the deep tissue component can more easily be distinguished from the more surficial dermal component. There is a long tradition in the trionychid literature of referring to the metaplastically ossified portions of the plastron as callosities (e.g., Gray, 1831). As the mode of growth appears to be the same across the shell (Scheyer 2009), I here apply this term to the metaplastic portions of the carapace as well.

Although the vast majority of authors use a relatively consistent nomenclature for the shell, some inconsistencies exist (e.g., Meylan, 1987 versus Kordikova, 2002, see below). I, here, use the following nomenclature: the carapace consists of neurals and costals, which are the metaplastic callosities of the underlying dorsal vertebrae and ribs. The nuchal consists of a superficial callosity and underlying processes. Pan-trionychids variously develop neomorphic preneurals, prenuchals, and peripherals. My rationale for using the term preneural is explained in detail below (see Discussion). The plastron consists of an entoplastron and paired epi-, hyo-, hypo-, and xiphiplastra. As with the nuchal, all superficial ossifications, if present, are termed callosities, while the deeper, strut-like deep tissues are referred to as processes. Karl (1997) proposed a nomenclatural scheme for these processes, but this system is rarely used, perhaps because the long Latin names are too cumbersome. As an alternative, I am here using a simplified English nomenclature, where the epiplastron has an anterior and posterior process, the entoplastron a pair of lateral processes, the hyoplastron lateral and medial processes, the hypoplastron lateral and posteromedial processes, the latter of which can be subdivided into medial and posterior processes, and the xiphiplastron anterior, medial, and posterior processes. To avoid cluttering the images with labels, all nomenclature is fully labelled in Fig. 1, but otherwise only color coded.

### Characters

All current pan-trionychid phylogenies utilizing morphology as their primary source of data can be traced back to the analysis of Meylan (1987), who developed 59 phylogenetically informative skeletal characters. To address issues associated with continuous characters and polymorphism, Meylan (1987) developed 24 multistate characters, of which 9 utilize scaled coding (sensu Wiens, 1995; e.g., 0 = absent; 1 = polymorphic; 2 = present). Meylan (1987) also developed 6 morphometric characters and 4 characters that utilize ontogenetic observations. 23 characters pertain to the shell. Over the course of the last two decades, new shell characters were added to this matrix by Joyce et al. (2009), Joyce and Lyson (2011, 2017), Vitek (2011, 2012), Brinkman et al. (2017), Vitek et al. (2018), and Jasinski et al., 2022, but a systematic revision of the initial characters is still outstanding.

In an attempt to create an exhaustive character list, all previously developed shell characters were organized by anatomical region. Each anatomical system was then examined one by one in search of character expansions or new characters. The 69 characters listed below were developed using the following guidelines. Further details are otherwise listed with each character below.

Discrete characters: Of the 69 characters developed herein, only 15 utilize naturally-discrete character states (e.g., the presence of a prenuchal, peripherals, a preneural, or supernumerary plastral ossifications; the shape, orientation, and number of neurals; the number of lateral hyo- and hypoplastral processes and medial xiphiplastral processes). To utilize all available character variation, morphoclines were developed that pertain to the full spectrum of available observations (e.g., one, two, or three lateral hyoplastral processes). To ease scoring, character states were labelled, when possible, with numbers that match the anatomy they denote (e.g., character state 6 marks a neural reversal at neural 6).

Continuous and morphometric characters: 40 of the 69 characters developed herein utilize segments within a factual or theoretical continuum of observations. To avoid vague, qualitative character state delimitations, characters were quantified where possible. For this purpose, length, surface, and angle measurements were obtained from photographs using the public domain program ImageJ (Schneider et al., 2012). Measurements were chosen that remain mostly undistorted in views typically captured in photographs (e.g., straight dorsal and ventral views). The available morphospace was then evenly subdivided into two or more character states that form morphoclines, typically at round numbers, to ease scoring. To avoid overemphasizing changes at the chosen thresholds while acknowledging measurement uncertainly, specimens nearing a given threshold by ± 10–15% of the interval assigned to a particular character were scored polymorphic across the boundary. These values were chosen because they represent typical margins of error in biological systems. The remaining, non-morphometric continuous characters were otherwise worded as carefully as possible to allow scoring objectively.

### Initial character scoring and minor abnormalities

All 69 newly developed shell characters were scored separately for the preselected set of 530 specimens using the freeware program Mesquite 3.81 (Maddison & Maddison, 2023). As many specimens are incomplete or insufficiently documented in the available pictures, about one third of the cells are scored unknown.

Note was taken of all specimens affected by minor abnormalities. The most common minor abnormalities observed in the available sample are right/left asymmetries in the lateral contacts of the neural column (about 28% of individuals) and right/left asymmetries in costal count (e.g., *Lissemys scutata* CUMZ T154, which lacks its eighth left costal; *Apalone mutica* YPM R11680, which displays a supernumerary first right costal), both of which are typically the result of a scoliotic dorsal vertebral column. Additional, minor abnormalities include the presence of supernumerary neurals and paired costals (e.g., *Pelodiscus* sp. USNM 539335) and various post-natal pathologies. Individuals with extreme abnormalities, such as kyphotic shells, were omitted from the study completely in a previous step (see Sampling above). All polymorphism was recorded using the backslash symbol.

All primary measurements and abnormalities were documented in the comments section of each cell (see Additional file 1).

The scoring of all individuals serves as the bases for the final coding of each terminal taxon (see below). Their listing at the level of the individual will allow future researchers to add additional specimens to the matrix or apply alternative coding strategies.

### Sexual dimorphism as a source of polymorphism

The vast majority of living trionychids show no apparent sexual dimorphism beyond the relative length of the tail. Indeed, sexual dimorphism is so minor that the topic of sexual dimorphism is barely raised in species accounts (see https://iucn-tftsg.org/cbftt/). A clear exception are the three living species of *Apalone*, where the female is substantially larger than the male, but no osteological differences to the shell have previously been noted between the sexes (Webb, 1962). Although the possibility remains that the shell varies systematically between the sexes for the characters developed herein, this seems implausible, as sexual differences typically have a functional basis (e.g., combat, mating, or oviposition). Unfortunately, the sex of most animals in the present sample is neither listed on the tags, nor apparent from the skeleton. Indeed, while it is possible to ascertain that large individuals of all three *Apalone* specimens plausibly represent females, there is no rationale for identifying medium sized individuals as males, as these might represent subadult females as well. It is therefore not possible to test explicitly for a correlation between character development and the sexes. As is, the coding method developed herein favors the characteristics seen in *Apalone* females as they are the larger of the two sexes. Given that I am unable to spot any other systematic differences, I presume that future studies are not likely to find considerable sexual dimorphism and that the coding method implemented herein will not be proven to be problematic, at least in regard to sexual dimorphism. The assertion is supported by the observation that other groups of turtles often show great amounts of sexual size dimorphism (Berry & Shine, 1980), but only minor shape differences between the sexes (Ernst & Barbour, 1989).

### Ontogeny as a source of polymorphism

As much variation across pan-trionychids is plausibly correlated with ontogeny (Gardner & Russell, 1994), all characters were tested for a correlation with size. For this purpose, the straight carapace length of all animals was collected, typically using ImageJ from pictures of the carapace and a scale. The straight carapace length of each individual was then divided by the straight carapace length of the largest known individual of its species (see Character 1 below for list of sources) to obtain a value in percent expressing the relative size of the individual relative to its greatest potential size. This number serves as a proxy for the skeletal maturity of the specimen. The character scoring of all individuals and their relative skeletal size were then ranked in Excel using the RANK.AVG function and the Spearman's rank-order correlation coefficient (hereafter *SCC*) was then calculated using the COREL function. I here classify correlation values from 0 to 0.25 to be absent, from 0.25 to 0.5 to be weak, from 0.5 to 0.75 to be moderate, and from 0.75 to 1.00 to be strong. The highest values found in the present sample are in the high 80 s for characters with apparent correlation with ontogeny, so values in the 90 s are neither found, nor expected for this biological system. As the available sample of osteological specimens strongly undersamples juvenile specimens (only 6% of specimens originate from the lower quartile), any correlation found is likely an underestimate. This conclusion is supported by the observation that higher correlations are found for taxa with better sampling of juveniles (e.g., *Amyda ornata*). The identification of characters as being controlled by ontogeny allows adjusting for this source of polymorphism when establishing the coding of species.

### Coding of species

I implemented a mixed coding strategy for all terminal taxa that reduces the amount of polymorphism observed in the primary sample by utilizing the character states found in the most skeletally mature individuals of a given species for ontogenetically controlled characters (not the average, as suggested by Gardner & Russell, 1994) and by only scoring character states that occur with a frequency of at least 20%. I select a 20% threshold over the use of a method that uses exact frequencies (see Wiens, 1995, 1999 for examples) for two primary reasons. First, I am not confident that my sample is sufficiently random and large to correctly document frequencies in modern turtle populations. And second, frequency methods are difficult to apply to characters that include more than three character states. The 20% threshold is intended to suppress minor polymorphism based on regular phenotypic plasticity. As all primary observations based on individuals are made available as part of this study, future research will be able to integrate additional specimens and adjust their coding strategy. To allow exploring the impact of my coding strategy, I created a secondary set of terminals (“MaxPoly”) that encode all polymorphism found in the primary dataset.

The full character matrix, including the primary coding of species of the scoring of all individuals, is provided in Additional file 1.

### Phylogenetic analysis

The character taxon matrix was imported into TNT 1.5 (Goloboff & Catalano, 2016) for parsimony analysis. Unless stated otherwise, all settings were left as per default. The tree buffer size was set to 40,000 trees. For all analyses, 1000 replicates of random addition sequences were followed by a round of tree bisection and reconnection. Numerous analyses were performed that vary in the ordering of characters that form morphoclines (i.e., characters 1, 3, 5, 6, 8, 10, 11, 17, 18, 21, 22, 24, 26, 31, 34, 35, 39, 41–44, 47, 48, 51–54, 56, 58–62, 65, 66, 68), the use of implied weighting (K = 3 and 6; Ezcurra, 2024; Goloboff et al., 2018), the use of an outgroup (i.e., the adocid *Adocus amtgai*, the carettochelyid *Carettochelys insculpta*, and the Early Cretaceous pan-trionychid *Perochelys lamadongensis*), and the selection of terminals (see Discussion below for the exact sequence of analyses). Analyses run without an outgroup were secondarily rooted after the analysis to create a monophyletic *Cyclanorbinae* and *Trionychinae*. As a way to assess the phylogenetic accuracy of a given analysis (i.e., its convergence upon the molecular signal), all most parsimonious trees of a given analysis were compared to the emerging molecular consensus (see Discussion below) by calculating quartet-based measures (Estabrook et al., 1985) using the R package Quartet v1.2.6 (Smith, 2019). For simplicity, average results are reported. This metric was chosen over distance metrics, such as Robinson-Foulds, as information is utilized from the relationships of four taxa, instead of just two. I purposefully did not compare the emerging molecular consensus to strict consensus trees, as strict consensus trees universally include unresolved nodes, which can inflate numbers, as polytomies are ignored. This demands weighing accuracy against resolution. The results are reported and discussed below (see Discussion).

### Institutional abbreviations

AMNH, American Museum of Natural History, New York, USA; BMNH, Natural History Museum, London, UK; CUMZ, Chulalongkorn University Museum of Natural History, Bangkok, Thailand; EOWL, EO Wilson Laboratory, Gorongosa National Park, Mozambique; FMNH, Field Museum of Natural History, Chicago, Illinois, USA; IVPP, Institute of Vertebrate Paleontology and Paleoanthropology, Beijing, China; KU, University of Kansas Natural History Museum, Lawrence, Kansas, USA; MCZ, Museum of Comparative Zoology, Cambridge, Massachusetts, USA; MNHN, Muséum national d’Histoire naturelle, Paris, France; MTD, Museum für Tierkunde Dresden, Dresden, Germany; NDGS, North Dakota Geological Survey at the North Dakota Heritage Center and State Museum, Bismarck, North Dakota, USA; NHMB, Naturhistorisches Museum Basel, Basel, Switzerland; NMB, Museum für Naturkunde Berlin, Berlin, Germany; NMW, Naturhistorisches Museum Wien, Vienna, Austria; OMNH, Sam Noble Museum, Norman, Oklahoma, USA; PCHP, Peter C. H. Pritchard Collection at the Turtle Conservancy, Ojai, California, USA; RH, Ren Hirayama Collection at Waseda University, Tokyo, Japan; ROM, Royal Ontario Museum, Toronto, Canada; SMF, Senckenberg Naturmuseum, Frankfurt, Germany; TMM, Texas Memorial Museum, Austin, Texas, USA; TMP, Tyrrell Museum of Palaeontology, Drumheller, Alberta, Canada; UCMP, University of California Museum of Paleontology, Berkeley, California, USA; UCMVZ, Museum of Vertebrate Zoology, Berkeley, California, USA; UMMZ, Museum of Zoology, Ann Arbor, Michigan, USA; USNM, National Museum of Natural History, Washington DC, USA; VNUH, Vietnam National University, Hanoi, Vietnam; YPM, Yale Peabody Museum of Natural History, New Haven, Connecticut, USA; ZMC, Zoologisk Museum, Copenhagen, Denmark; ZSI, Zoological Survey of India, Kolkata, India.

## List of characters

### Character 1: Carapacial disk size

**Fig. 2:**
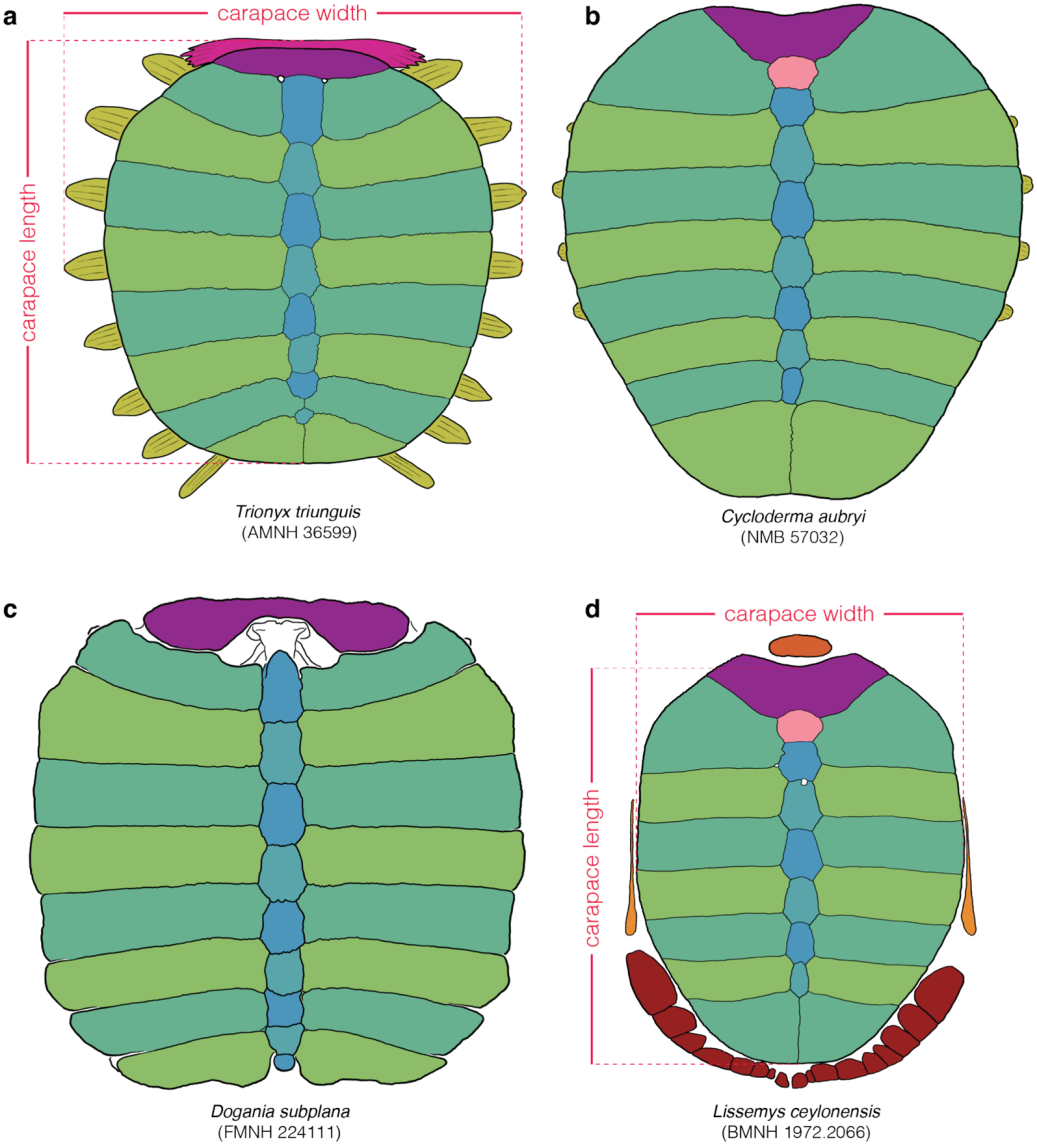
Line drawings of select carapaces in dorsal view highlighting variation in bone development: **a** *Trionyx triunguis* (AMNH 36599), **b** *Cycloderma aubryi* (NMB 57032), **c** *Dogania subplana* (FMNH 224111), **d** *Lissemys ceylonensis* (BMNH 1972.2066). Images are not to scale. Colors are used to highlight the anatomical systems labelled in Fig. 1

*Character Definition:* Maximum straight median length of the bony carapacial disk of skeletally mature specimens, including the nuchal, neurals, and costals (Fig. 2a), but excluding the prenuchal and peripherals (Fig. 2d) (modified from Meylan, 1987, p. 24; Valdes et al., 2017, p. 20; Vitek, 2012, p. 85): 0 =  < 10 cm; 1 = 10–19.5 cm; 2 = 20–29.5 cm; 3 = 30–39.5 cm; 4 = 40–49.5 cm; 5 = 50–59.5 cm; 6 = 60–69.5 cm; 7 = 70–79.5 cm; 8 = 80–89.5 cm; 9 =  ≥ 90 cm.

Alternative character states: 0 =  < 20 cm; 1 = 20–39.5 cm; 2 = 40–59.5 cm; 3 = 60–79.5 cm; 4 =  ≥ 80 cm.

Comments: Meylan (1987) developed a character that groups all small-bodied trionychids (bony carapace disk length less than 20 cm) relative to all larger-bodied trionychids. As some fossil pan-trionychids are notably large, Vitek (2012) formulated an additional character that groups all “giant” pan-trionychids (bony carapace disk length greater than 60 cm). The bony carapacial disk of adult pan-trionychids in fact varies gradually from approximately 10 to 100 cm in straight median length (Hutchison, 2009; Pritchard, 2001; Vitek, 2012). I, therefore, here phrase a single character that evenly divides the full span into ten character states that form a morphocline. Naturally, the length of the bony carapace does not correspond to the length of the carapace itself in all extant trionychids (Pritchard, 2001). As many phylogenetic programs do not allow for the usage of 10 character states, I provide an alternative coding for this character that evenly divides the available morphocline in five character states.

The vast majority of measurements used herein were obtained from photographs using ImageJ and, therefore, only approximate the straight midline length as observed in dorsal view, not the midline length over the curve (Fig. 2). To highlight uncertainty, all measurements were rounded to the next half centimeter. In rare cases, for instance when a specimen is lacking its carapace, coarse estimates were obtained using other body parts and ratios apparent from other specimens, often with broad margins of uncertainty.

The final scoring of each species is based on the largest documented specimen. As this study does not necessarily sample the largest known individuals for each extant species, this was in part achieved by critical reevaluating record specimens listed by Meylan (1987) and Pritchard (2001). However, records were only utilized if they are based on straight measurements and specimens housed in museum collections. A summary with annotations is provided in Table 1.

**Table 1:** Maximum documented straight median length of the bony carapacial disk of extant trionychids

	Meylan (1987)	Pritchard (2001)	New estimate	Sources
*Cyclanorbinae*				
*Co. elegans*	475 mm	490 mm	ca. 47.5 cm	(NMW 1437; Meylan, 1987)
*Co. senegalensis*	325 mm^1^	325 mm	ca. 23.5 cm	(BMNH 1949.1.3.57; Meylan, 1987)
*Cd. aubryi*	365 mm	365 mm	ca. 42.5 cm	(NMB 8697b; new record)
*Cd. frenatum*	535 mm	535 mm	ca. 53.5 cm	(BMNH 1947.3.4.16; Meylan, 1987)
*Li. ceylonensis*	*–*	*–*	ca. 32.5 cm	(NHMB 2398; new record)
*Li. punctata*	370 mm^2^	370 mm2	ca. 25.5 cm	(NMW 1232; new record)
*Li. scutata*	–	230 mm^3^	ca. 20.5 cm	(CUMZ T169; new record)
*Amydini*				
*A. cartilaginea*	316 mm^4^	563 mm^4^	ca. 44.0 cm	(RH129; new record)
*A. ornata*	–	–	ca. 57.0 cm	(CUMZ T128; Pritchard, 2001)
*D. subplana*	250 mm	217 mm	ca. 19.5 cm	(PCHP 4674; Pritchard, 2001)
*N. formosa*	274 mm	420 mm	ca. 34.5 cm	(PCHP 5035; Pritchard, 2001)
*N. gangetica*	485 mm^5^	600 mm	ca. 47.5 cm^5^	(ZSI 1893; Annandale, 1912)
*N. hurum*	416 mm	416 mm	ca. 41.5 cm	(ZSI 16752; Annadale, 1912)
*N. leithii*	380 mm^6^	380 mm	ca. 38.9 cm^6^	(FMNH 224231; Meylan, 1987)
*N. nigricans*	403 mm^7^	550 mm	ca. 38.5 cm^7^	(ZSI 1994; Annandale, 1912)
*Pa. steindachneri*	170 mm	300 mm	ca. 20.0 cm	(RH 1201; new record)
*Pelodiscus sp.*	201 mm	201 mm	ca. 20.0 cm	(ZSM 429/1911; Meylan, 1987)
*Chitrini*				
*C. chitra*	550 mm^8^	738 mm	ca. 79.0 cm	(CUMZ exhibit, new record)
*C. indica*	–	724 mm	ca. 49.0 cm	(NHMB 2706, new record)
*C. vandijki*	*–-*	*–*	?	(data insufficient)
*Pc. bibroni*	–	745 mm	ca. 42.0 cm	(PCHP 11230, new record)
*Pc. cantorii*	415 mm^9^	730 mm	ca. 42.5 cm	(CUMZ T120, new record)
*Pc. signifera*	–	–	?	(data insufficient)
*Apalonini*				
*A. ferox*	371 mm	442 mm	ca. 37.0 cm	(UF 45341; Meylan, 1987)
*A. mutica*	124 mm	180 mm^10^	ca. 18.0 cm	(FMNH 22064; new record)
*A. spinifera*	186.5 mm	288 mm	ca. 25.0 cm	(YPM 10892; new record)
*R. euphraticus*	282 mm	450 mm	ca. 31.0 cm	(PCHP 4062; Pritchard, 2001)
*R. swinhoei*	490 mm	635 mm	ca. 63.5 cm	(Ngoc Son Temple; Pritchard, 2001)
*T. triunguis*	410 mm	410 mm	ca. 52.0 cm	(NMW 32237, new record)

^1^Meylan (1987) reported BMNH 1949.1.3. to measure 325 mm, but this appears to be a typo, as my own measurements indicate this specimen to be about 235 mm or 23.5 cm

^2^The record specimen listed by Meylan (1987) and Pritchard (2001) for *Lissemys punctata* was reported by Deraniyagala (1939) from Sri Lanka and therefore represents *Lissemys ceylonensis*. This specimen does not appear to be housed in a museum and the measurement was likely taken over the curse. It is therefore disregarded herein

^3^The measurement listed by Pritchard (2001) for *Lissemys scutata* are over the curve of the carapace

^4^The records listed by Meylan (1987) and Pritchard (2001) for *Amyda cartilaginea* originate from specimens now attributable to *Amyda ornata*

^5^Annandale's measurement appears to originate from ZSI 1893, which we, however, only measured to have a length of about 47.5 cm

^6^Meylan's (1987) specimen EOM 2819 is now catalogued as FMNH 224231

^7^Annandale's measurement appears to originate from ZSI 1994, which we, however, only measured to have a length of about 38.5 cm

^8^The record listed by Meylan (1987) for *Amyda indicate* originate from specimens now attributable to *Chitra chitra*

^9^The record listed by Meylan (1987) for *Pelochelys bibroni* originate from specimens now attributable to *Pelochelys cantori*

^10^Pritchard’s (2001) record specimen, PCHP 2746, is here reidentified as *Apalone spinifera*, not *Apalone mutica*

As a way to explore correlations between ontogeny and other characters, I utilize the relative size of each individual as a proxy for its relative skeletal maturity. For this purpose, I divided the estimated straight carapace length of each individual by the largest known straight carapace length of its species. The resulting value is expressed in percent. For simplicity, specimens with a size range are represented by the midpoint of their size estimate. All obtained values can be found in the comments section of each individual for this character (see Additional file 1).

### Character 2: Carapacial disk outline

*Character Definition:* Carapacial disk outline of skeletally mature specimens (modified from Meylan, 1987, p. 25): 0 = overall rounded (Fig. 2a, c); 1 = posterior half constricted, posterior costals much narrower than anterior costals (Fig. 2b, d).

*Comments:* In the vast majority of pan-trionychids, the carapacial disk is a rounded structure that is about as wide anteriorly as posteriorly (state 0; Fig. 2a, c). In a small assortment of cyclanorbines, however, the posterior costals are notably shorter mediolaterally, resulting in a carapacial disk that is constricted posteriorly (state 1; Fig. 2b, d). This space either remains empty, as in *Cycloderma aubryi* (Fig. 2b), or is partially filled by neomorphic peripherals, as in *Lissemys ceylonensis* (Fig. 2d). Specimens that exhibit an intermediate condition were liberally scored polymorphic (i.e., 0/1).

Although this characteristic is clearly developed as present or absent in most trionychids, high levels of polymorphism are present in *Cyclanorbis senegalensis*, with various individuals exhibiting a rounded carapace, a posteriorly constricted one, or an intermediate one. Statistical analysis do not find a relationship with ontogeny for this taxon (*SCC* = − *0.20*; see Additional file 2). I, therefore, code this taxon as polymorphic, in contrast to Meylan (1987), who coded this taxon as absent. My scorings otherwise fully agree with those of Meylan (1987).

### Character 3: Carapacial Sculpturing 1

**Fig. 3:**
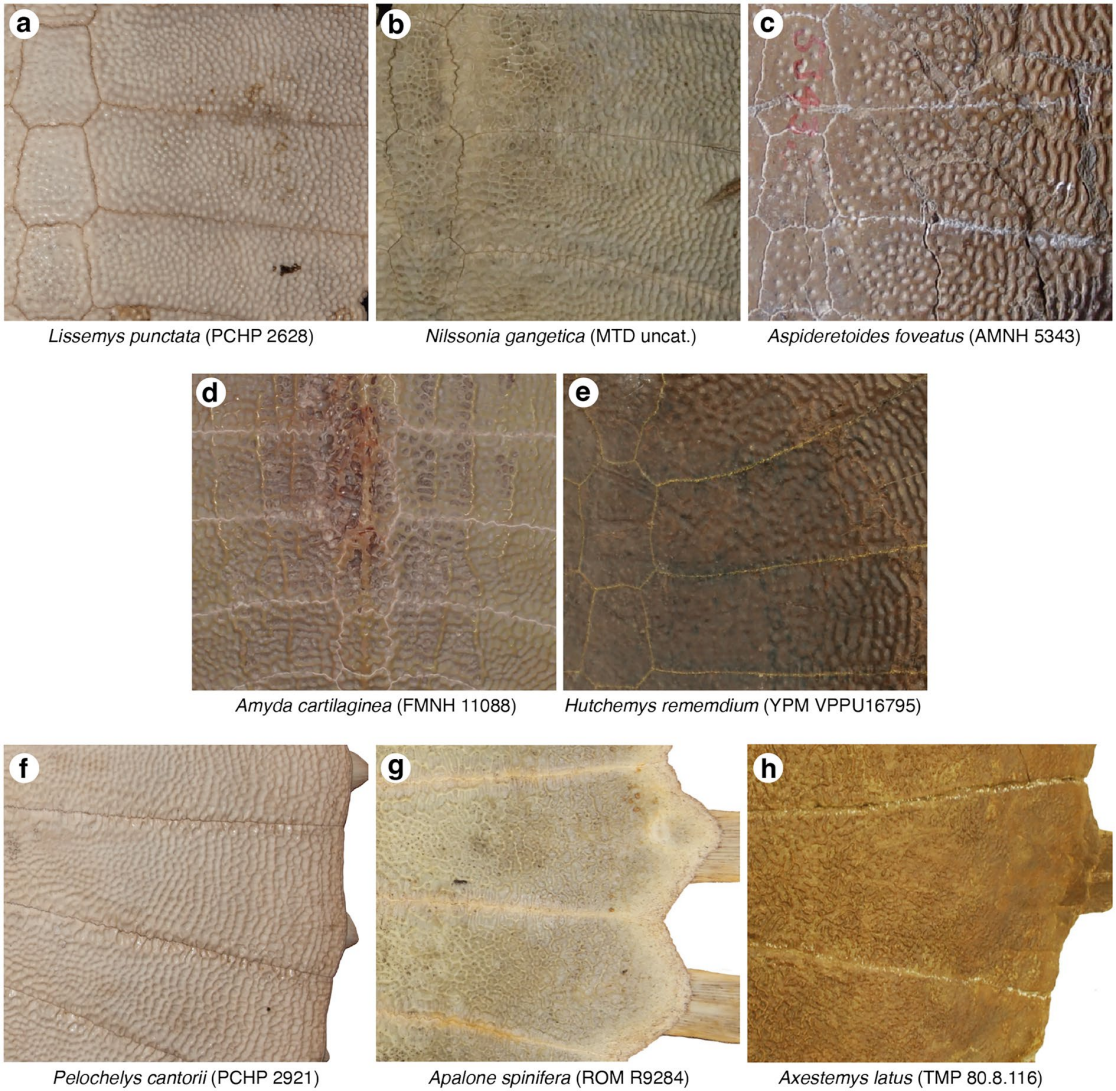
Photographs of select carapaces in dorsal view highlighting details in surface texture: **a** *Lissemys punctata* (PCHP 2628), **b** *Nilssonia gangetica* (MTD uncat.), **c** *Aspideretoides foveatus* (AMNH 5343), **d** *Amyda cartilaginea* (FMNH 11088), **e** *Hutchemys rememdium* (YPM PU16795), **f** *Pelochelys cantorii* (PCHP 2921), **g** *Apalone spinifera* (ROM R9284), **h** *Axestemys latus* (TMP 80.8.116). Images are not to scale

*Character Definition:* Carapacial surface sculpturing (new character): 0 = mostly consists of raised tubercles (Fig. 3a); 1 = mostly consists of a netted pattern formed by raised ridges (Fig. 3b); 2 = mostly consists of distinct circular depressions (Fig. 3c).

*Comments:* The surface of pan-trionychid carapaces is ornamented by a distinct surface texture that allows recognizing fossil shell fragments with ease (Hutchison & Archibald, 1986). Variations to this surface texture were historically used to diagnose extinct species (e.g., Hay, 1908), but more recent work agrees that substantial variation at the individual and species level preclude utilizing surface texture in the rigorous diagnosis of species (e.g., Gardner & Russell, 1994; Georgalis & Joyce, 2017; Vitek & Joyce, 2015). Although I agree that nuanced variations in surface texture should not be used to diagnose extinct species, general texture patterns are nevertheless relatively stable. I recognize three broad categories of carapacial surface texture. The first consists of raised tubercles and is typical among extant cyclanorbines (state 0; Fig. 3a). The second consists of a netted network of narrow, reticulating ridges (state 1; Fig. 3b). This is the dominant pattern among pan-trionychids throughout their evolutionary history. The third consists of distinct circular depressions (state 2; Fig. 3c). This pattern is mostly found among extinct pan-trionychids, such as the late Campanian *Aspideretoides foveatus* (Gardner et al., 1995; Leidy, 1860).

The external compact bone of pan-trionychid shell callosities consists of a deeper ply-wood layer and a surficial layer that forms the sculpturing (Scheyer et al., 2007). Informal observations suggest that the surficial layer becomes thicker during ontogeny and often grades in thickness from the outside to the inside of the callosities. Some taxa thus document a gradation from no sculpturing to isolated tubercles (Fig. 3h), or from isolated tubercles to a netted pattern, or from a netted pattern to a rain drop pattern (Fig. 3c, e). As a result, while many specimens score either 0, 1, or 2, all individuals with intermediate surface texture either score 0/1 or 1/2, but not 0/2. As such, it appears that the three character states I recognize herein form a morphocline.

As many individuals show variation across the shell (e.g., Fig. 3c, e, g, h), I scored this character by reference to the dominant pattern apparent across the carapace, while liberally scoring individuals polymorphic if two morphotypes are equally predominant or if intermediate morphotypes are apparent (e.g., raised tubercles that partially coalesce to ridges, as in *Cycloderma aubryi*). However, I only coded species polymorphic, if polymorphism is consistently found across the species (i.e., found in more than 20% of individuals). At the species level, polymorphism (i.e., variation across the shell) is prevalent in *Apalone mutica, Apalone spinifera*, *Cyclanorbis* spp., and *Cycloderma* spp. *Lissemys* spp. consistently score 0 while the remaining trionychines consistently score 1.

### Character 4: Carapacial Sculpturing 2

*Character Definition:* Longitudinal carapacial striations in skeletally mature specimens (modified from Joyce et al., 2018, p. 95): 0 = absent (Fig. 3a, b, c, e, f, g, h); 1 = present (Fig. 3d).

*Comments:* The carapace of many pan-trionychids are decorated with anteroposteriorly arranged striations (state 1; Fig. 3d), either raised ridges, as in some extant *Amyda cartilaginea* or *Apalone ferox*, or depressed sulci, as in the extinct *Gilmoremys lancensis* (Gilmore, 1916; Joyce & Lyson, 2011). Statistical analysis of the data obtained from all extant trionychids reveals a weak, almost moderate negative correlation with ontogeny (*SCC* = − *0.49*; see Additional file 2), thereby generally supporting the previously phrased notion that striations fade during ontogeny (e.g., Joyce et al., 2018; Massonne et al., 2023). However, values range from absent to weak when taxa are analyzed individually (e.g., *SCC* = − *0.50* for *Cycloderma frenatum*; *SCC* = − *0.33* for *Amyda ornata*; *SCC* =  + *0.22* for *Apalone ferox*; see Additional file 2). I, nevertheless, maintain the original character concept of Joyce et al. (2018) and mostly code species by reference to the most skeletally mature specimens available. In my sample of extant trionychids, striations are variously developed among the adults of *Amyda* spp., *Apalone ferox*, and *Pelodiscus* sp. I, therefore, coded these taxa as polymorphic. Although this character is not parsimony informative among extant trionychids, as no extant taxon unambiguously exhibits the derived character states, it is retained, as numerous fossil taxa exhibit unambiguous carapacial striations.

### Character 5: Carapacial sculpturing 3

*Character Definition:* Fading of sculpturing from the margins to the center of the carapace (modified from Joyce et al., 2009, p. 67): 0 = absent (Fig. 3b, d); 1 = present, but minor (Fig. 3a); 2 = present and distinct (Fig. 3c, e).

*Comments:* In the vast majority of pan-trionychids, the surface of the carapace is decorated by a surface texture that does not differ substantially across the carapace (state 0; Fig. 3b, d). This contrasts a small group of taxa, where the surface texture either fades distinctly from the periphery towards the center (state 1; Fig. 3a), such as in many cyclanorbines, or disappears completely (state 2; Fig. 3c, e), such as in the extinct plastomenid *Hutchemys* spp. (Girard et al., 2024; Joyce et al., 2009). Among extant trionychids, this characteristic is mostly found among cyclanorbines, which, however, show no correlation of this characteristic with ontogeny (*SCC* = *0.01*; see Additional file 2). I, therefore, code taxa as polymorphic if a character state has a frequency above 20%, regardless of the relative skeletal maturity of the individuals in the sample. The three states for a morphocline that can be ordered.

### Character 6: Carapacial sculpturing 4

*Character Definition:* Lateral extent of surficial sculpturing of costals in skeletally mature specimens (new character): 0 = sculpturing reaches margins of costals (Fig. 3f); 1 = sculpturing does not reach margins of costals, 10% or less of costal margin is smooth (Fig. 3g); 2 = sculpturing does not reach margins of costals, more than 10% of costal margin is smooth (Fig. 3h).

*Comments:* I recognize three states that pertain to the development of sculpturing along the distal margins of the costals and that form a morphocline that can be ordered. In the vast majority of pan-trionychids, the sculpturing reaches the distal margins of the costals (state 0; Fig. 3f), but a narrow (state 1, Fig. 3g), or broad (state 2, Fig. 3h) strip lacking sculpturing is developed along the distal margins of others. Gardner and Russell (1994) documented that this character is somewhat variable in some pan-trionychids, but consistently developed in others. This observation is broadly confirmed herein. While statistical analysis of all extant trionychines only suggests a weak negative correlation with ontogeny (*SCC* = − *0.38*; see Additional file 2), moderate negative correlations are apparent when better sampled taxa are analyzed alone (*SCC* = − *0.54* for *Amyda ornata*; *SCC* = − *0.53* for *Apalone ferox*; see Additional file 2). I, therefore, code each taxon based on the morphology apparent in the most adult individuals.

Fully covered costals are persistently present in the vast majority of skeletally mature extant pan-trionychids. A narrow strip lacking sculpturing occurs occasionally in the adults of some taxa (e.g., *Apalone spinifera*, *Pelodiscus sinensis*, coded polymorphic), but is persistently present in others (e.g., *Rafetus euphraticus*). The condition with the broad strip lacking sculpturing is only found in fossil forms, such as the extinct *Axestemys quinni* (Schmidt, 1945; Vitek, 2012).

### Character 7: Carapacial kinesis

Character definition: Carapacial kinesis within the carapacial disk in skeletally mature specimens (new character): 0 = absent (Fig. 2a, b, d); 1 = present (Fig. 2c).

**Fig. 4:**
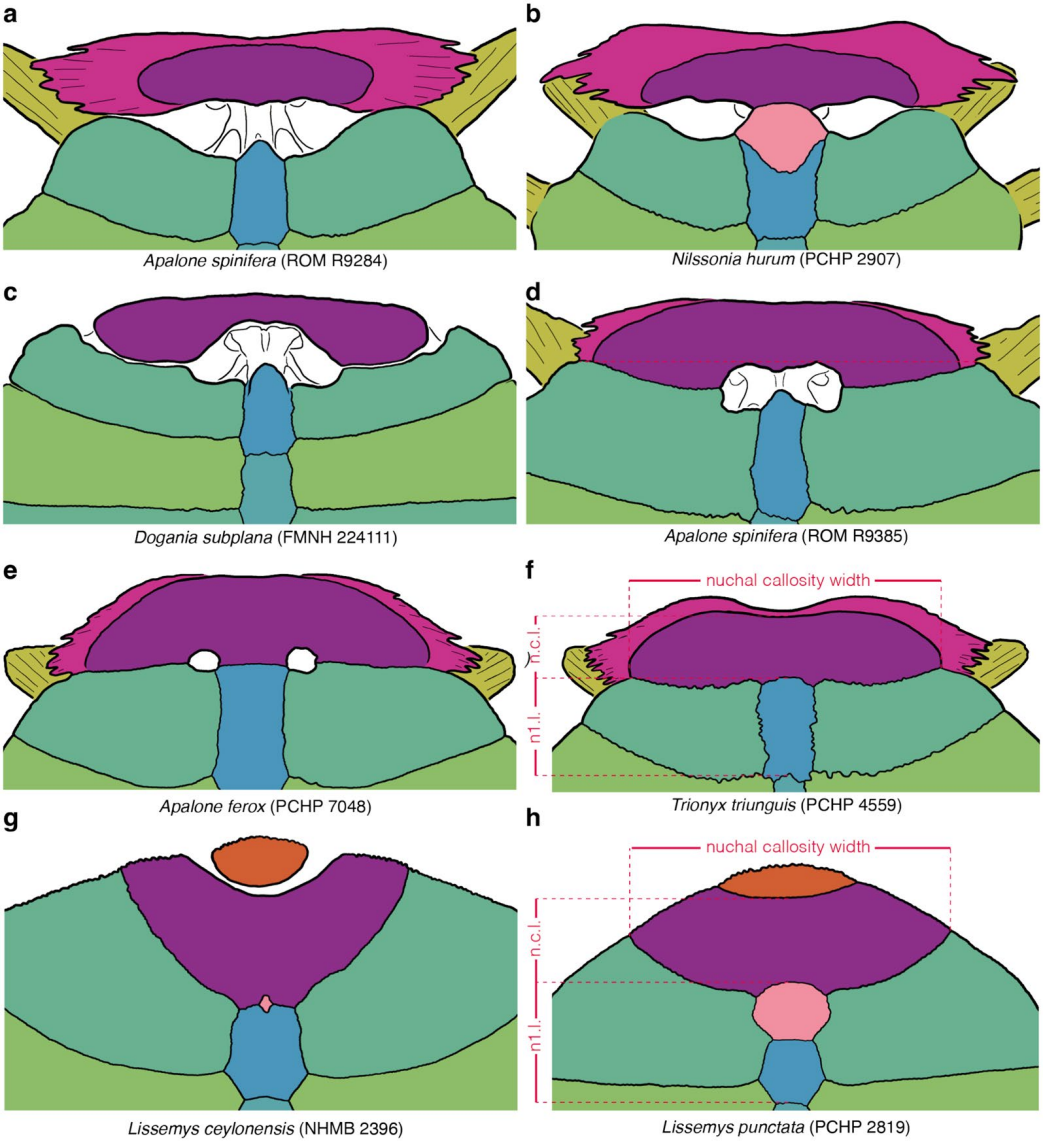
Line drawings of the anterior part of select carapaces in dorsal view highlighting variation in bone development: **a** *Apalone spinifera* (ROM R9385), **b** *Nilssonia hurum* (PCHP 2907), **c** *Dogania subplana* (FMNH 224111), **d** *Apalone spinifera* (ROM R9284), **e** *Apalone ferox* (PCHP 7048), **f** *Trionyx triunguis* (PCHP 4559), **g** *Lissemys ceylonensis* (NHMB 2396), **h** *Lissemys punctata* (PCHP 2819). Images are not to scale. Colors are used to highlight the anatomical systems labelled in Fig. 1. Anatomical nomenclature is highlighted in Fig. 1. n1.l., neural 1 length; n.c.l., nuchal callosity length

Comments: In the vast majority of subadult to adult pan-trionychids, the regular elements of the carapace (i.e., nuchal, neurals, and costals) are sutured to form a solid structure (state 0; Fig. 2a, b, d). Pritchard (1993) reported that the carapace of *Dogania subplana* shows evidence of what he termed "generalized carapacial kinesis" or "carapacial pankinesis," where many elements of the carapace are able to move relative to their neighbors (state 1, Fig. 2c). Pritchard (1993) concluded that this is achieved through the retention of syndesmotic contacts into adulthood and the formation of a complete row of neurals that fully separated the two rows of costals from one another. I, furthermore, note that the costals are mostly arranged transversally, that an uneven carapacial margin is retained in subadults and adults, and that a noticeable hinge, visible as a gap in dorsal view, is developed between the nuchal and the first pair of costals (Figs. 2c, 4c). I am unaware of any other taxon, extinct or extant, to display this set of characteristics. Hasan (1941) reported that the prenuchal and peripherals of *Lissemys punctata* are kinetically connected to the main carapacial disk, but this type of kinesis is disregarded for this character, as it is located outside of the main carapacial disk.

### Character 8: Prenuchal

Character definition: Prenuchal in skeletally mature specimens (modified from Meylan, 1987, 7): 0 = absent (Figs. 2a, b, c; 4a–f); 1 = present, but not articulated with shell in adult individuals (Figs. 2d; 4g); 2 = present and articulated with the shell in adult individuals (Fig. 4h).

Comments: The prenuchal is a neomorphic shell bone situated anterior to the nuchal (Meylan, 1987; Fig. 2d). It only appears in ontogeny after hatching (Delfino et al., 2010). The prenuchal is suturally attached to the carapacial disk in all herein studied subadult and adult specimens of *Lissemys punctata* (state 2; Fig. 4g), but is consistently surrounded by skin in *Lissemys ceylonensis*, *Lissemys scutata*, and *Cyclanorbis senegalensis* (state 1; Fig. 3h). As statistical analysis finds a moderate positive correlation of this characteristic with ontogeny for *Lissemys* punctata and *Lissemys scutata* (*SCC* = *0.59*; see Additional file 2), I code each taxon for the condition seen in the most adult available individuals, also as the prenuchal of hatchlings has been reported to be small or lacking in the affected species (McGovern et al., 2021; Stoffert, 1889).

As the presence of a prenuchal correlates strongly with the development of a deep nuchal notch in extant cyclanorbines, I suggest that fossils should be scored as lacking a prenuchal, if the anterior margin of the shell is straight or convex. Fossil cyclanorbines, turtles for which there is reason to believe that a prenuchal may have been present, with a deep nuchal notch, on the other hand, should be scored uncertain, even when the prenuchal is absent, to highlight that a floating prenuchal may have been present, but was lost taphonomically. As the prenuchal appears in ontogeny unconnected to the rest of the carapace, I treat this character as a morphocline that can be ordered.

### Character 9: Neomorphic peripherals

Character definition: Neomorphic peripherals (as modified by Joyce et al., 2016, p. 5 from Meylan, 1987, p. 5): 0 = absent (Fig. 2a, b, c); 1 = present (Fig. 2d).

Comments: The analysis of Meylan (1987) was developed for a broad set of turtles and, therefore, included a character that counted the number of peripheral elements: 22, as in the vast majority of crown turtles, 20, as in kinosternids and carettochelyids, 14–18, as in *Lissemys* spp. (Fig. 2d), and 0, as in all other known pan-trionychids (Fig. 2a, b, c). As this analysis only pertains to pan-trionychids, I restrict this character to the peripheral elements that occur in *Lissemys* spp. (Fig. 2d), as suggested by Joyce et al. (2016).

There has been some debate regarding the homology of trionychid peripherals relative to those of other turtles. Boulenger (1889) and Zangerl (1969), for instance, suggested that the peripheral elements of *Lissemys* evolved independently (i.e., neomorphically) from those of other turtles, while Siebenrock (1909) and Hummel (1929) believed them to be rudimentary. Delfino et al. (2010) more recently concluded that the peripherals of this taxon are plausibly neomorphic, but also that they may share a deeper homology with the peripherals of other turtles and thus should be viewed as true peripherals. In the context of a more global analysis, I agree that it is prudent to regard all peripheral elements as homologous to reflect this deep homology. However, as the peripherals of *Lissemys* differ significantly from those of extant turtles by being too numerous and not being associated with the ribs (Brinkman et al., 2017), as I herein am interested in homology within *Pan-Trionychidae*, and as molecular data strongly suggests that *Lissemys* spp. is nested deeply within the tree (e.g., Thomson et al., 2021), I herein view the peripherals of this taxon as neomorphic, while acknowledging that this is a semantic preference.

The available sample confirms that the number of neomorphic peripherals varies among *Lissemys* spp., a conclusion drawn previously by Delfino et al. (2010) based on a larger sample. As the evolutionary history of neomorphic peripherals is unclear, special care should be accorded when scoring fossil cyclanorbines.

### Character 10: Nuchal callosity 1

Character definition: Width of nuchal callosity (Fig. 4f, h) relative to greatest carapacial disk width (Fig. 2a, d) as seen in dorsal view in skeletally mature specimens (modified from Meylan, 1987, 1): 0 =  ≤ 30%; 1 = 30–50%; 2 = 50–70%; 3 =  ≥ 70%. Specimens approaching a given threshold within 3 percentage points are scored polymorphic.

Comments: Meylan (1987) developed a character that captures variation to the width to length ratio of the nuchal. I decided to greatly expand this character for two reasons. First, the relative size and proportions of the nuchal processes varies independently from those of the nuchal callosity. As a result, the character of Meylan (1987) may group taxa that exhibit, for instance, a wide callosity and wide nuchal processes. Second, as phrased, the character may unite taxa that have similar nuchal proportions, even if the nuchal itself differs greatly in size relative to the full shell. I, therefore, here subdivide the character of Meylan (1987) into three separate characters that capture three different aspects: the width of the nuchal callosity relative to the carapacial disk (this character), the length of the nuchal callosity relative to length of costals I (see Character 11 below), and the width of the nuchal processes relative to the carapacial disk (see Character 18 below).

This character measures the width of the nuchal callosity (Fig. 4f, h) relative to the greatest width of the carapace, a measurement that includes the ribs (Fig. 2a, d). In all cases, measurements were obtained from photographs taken in dorsal view using ImageJ. Although straight dorsal views were preferred, the precise angle is not essential, as the two width measurements taken herein are distorted proportionally when viewing a specimen at an angle. As a full gradation is apparent, I subdivided this character into four character states that form a morphocline that can be ordered. Specimens that approach the arbitrarily selected thresholds between two character states within 3 percentage points are scored polymorphic (e.g., a specimen with a measurement of 53% is scored 1/2 as it approximates the threshold of 50% by three percentage points). This approach is chosen to downweigh thresholds and to address measurement error. As already highlighted by Gardner and Russell (1994), the size and proportions of the nuchal callosity changes substantially during ontogeny. Statistical analysis across all taxa confirms a moderate positive correlation in that more skeletally mature individuals have relatively wider nuchal callosities (*SCC* = *0.52*; see Additional file 2). Moderate to high values are retrieved for individual taxa (e.g., *SCC* = *0.88* for *Amyda ornata*; *SCC* = *0.78* for *Apalone ferox*; see Additional file 2). Species were, therefore, herein coded by reference to the most skeletally mature specimens. Whenever the most skeletally mature specimens approach the threshold between two character states, species were coded polymorphic.

### Character 11: Nuchal callosity 2

Character definition: Midline depth of the nuchal callosity (Fig. 4f, h) in anterior oblique view relative to the midline length of preneural and/or neural I (Fig. 4f, h) of skeletally mature individuals (modified from Meylan, 1987, 1): 0 = shallow, < 60%; 1 = intermediate, 60–100%; 2 = deep, > 100%. Specimens approaching a given threshold within 5 percentage points are scored polymorphic.

Comments: This is one of three characters derived from a character originally developed by Meylan (1987) pertaining to the shape of the nuchal (see Character 10 above).

I here recognize three character states that pertain to the midline length of the nuchal callosity relative to the midline length of the preneural and/or neural I (Fig. 4f, h): shallow (less than 60%), intermediate (60% to 100%), and deep (more than 100%). All measurements were taken based on photographs using ImageJ. However, as the midline depth of the nuchal is greatly distorted in a straight dorsal view, especially in highly domed specimens, measurements were obtained, where possible, based on photographs taken perpendicular to the nuchal region. If such photographs were unavailable, measurements were also obtained based on straight dorsal views of the carapace, but only for relatively flat specimens, as distortion is minimized. In all cases, measurement obtained from incorrectly oriented specimens underestimate the true value, as the nuchal appears shorter than it actually is in straight dorsal view. Specimens that approach the arbitrarily selected thresholds between two character states within 5 percentage points were scored polymorphic (see Character 10 above for example).

In contrast to the width of the nuchal callosity (see Character 10 above), statistical analysis across all taxa only shows no significant positive correlation with ontogeny in that more skeletally mature individuals only have slightly deeper nuchal callosities (*SCC* = *0.19*; see Additional file 2), but correlations vary from absent to strong for individual taxa (e.g., *SCC* = *0.10* for *Apalone ferox*; *SCC* = *0.23* for *Cyclanorbis senegalensis*; *SCC* = *0.84* for *Amyda ornata*; see Additional file 2). This suggests that the nuchal callosity of most taxa forms initially along the midline and then grows strongly towards the sides, but only minorly towards the anterior. I nonetheless code each taxon by reference to the most skeletally mature individuals, as no taxon shows a negative correlation with size. Species were coded polymorphic, if similarly sized adult individuals crossed the threshold, or if the most skeletally mature individual was scored polymorphic. The three available character states form a morphocline that can be ordered.

### Character 12: Nuchal callosity 3

Character definition: Formation of a nuchal notch by the nuchal callosity in skeletally mature specimens (modified from Joyce et al., 2009, p. 68): 0 = nuchal notch absent or minor (Fig. 4a–f); 1 = deep nuchal notch present (Fig. 4g, h).

Comments: The anterior margin of the shell of the vast majority of pan-trionychids is more or less straight (state 0; Fig. 4a, b, c, d, e, f), but a well-developed nuchal notch is formed by the nuchal callosity (not the nuchal processes) in an eclectic assortment of extinct and extant species, particularly cyclanorbines (state 1; Fig. 4g, h) and plastomenids (Girard et al., 2024). This notch is filled by the prenuchal in some cyclanorbines (see Character 8 above; Fig. 4h). Special care must be accorded to scoring highly domed specimens, as even a well-developed nuchal notch may be hidden from sight in dorsal view (Fig. 4h). An oblique view perpendicular to the nuchal is therefore favorable. In extant taxa with a developed nuchal notch, statistical analysis shows a weak positive correlation between the development of a nuchal notch and ontogeny (*SCC* = *0.43*; see Additional file 2). I, therefore, coded each species by reference to the most skeletally mature specimens.

### Character 13: Nuchal callosity 4

**Fig. 5:**
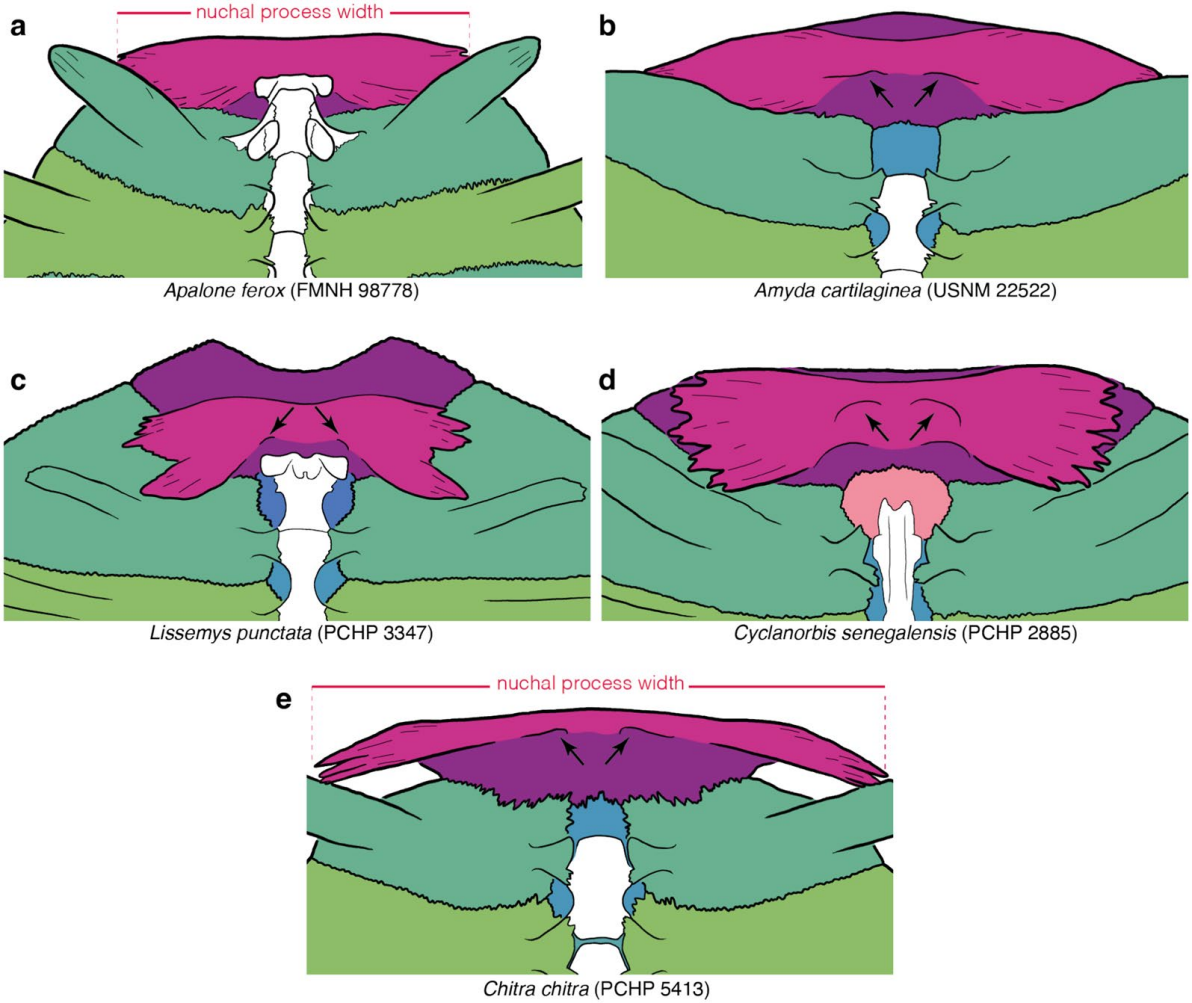
Line drawings of the anterior part of select carapaces in ventral view highlighting variation in bone development: **a** *Apalone ferox* (FMNH 98778), **b** *Amyda cartilaginea* (USNM 22522), **c** *Lissemys punctata* (PCHP 3347), **d** *Cyclanorbis senegalensis* (PCHP 2885), **e** *Chitra chitra* (PCHP 5413). Images are not to scale. Colors are used to highlight the anatomical systems labelled in Fig. 1. Anatomical nomenclature is highlighted in Fig. 1. Black arrows highlight location of paired depression on the ventral side of the nuchal

Character definition: Anterior protrusion of nuchal callosity beyond the anterior margins of the nuchal processes in skeletally mature specimens (new character): 0 = absent or minor (Fig. 5a, d, e); 1 = clear anteromedian protrusion is present (Fig. 5b); 2 = a clear protrusion is present along the entire anterior margin of the nuchal processes (Fig. 5c).

Comments: In the majority of pan-trionychids, the anterior margin of the nuchal callosity runs posterior or parallel to the anterior margin of the nuchal processes (Fig. 4a, b, c, d, e, f) and can therefore not or barely be seen in ventral view (state 0; Fig. 5a, b, d). In an eclectic assortment of species, mostly *Amyda* spp., the nuchal callosity protrudes significantly beyond the anterior margin of the nuchal processes along the midline (state 1; Fig. 5b; previously noted by Gardner & Russell, 1994), while in others, mostly cyclanorbines, the nuchal callosity protrudes significantly beyond the entire anterior margin of the nuchal processes (state 2; Fig. 5c). Although this characteristic shows no variation in most extant taxa, those that exhibit polymorphism show moderate to strong correlations with ontogeny, in that more mature individuals are more likely to display one of the two derived character states (*SCC* = *0.81* for *Cycloderma frenatum*; *SCC* = *0.40* for trionychine species with anterior protrusion; *SCC* = *0.81* for *Amyda ornata* only; see Additional file 2). The scoring for each species is therefore based on the most skeletally mature specimen available.

It is notable that among the present sample of extant trionychids, state 1 only occurs in trionychines and state 2 in cyclanorbines. The three available character states also do not form a logical morphocline. It is for these two reasons that I do not order this character.

This character should be scored using specimens where the anterior margin of the nuchal is neither embedded in skin, nor covered by dried tissue, as these may cache anterior protrusions. It is important to note that thick nuchal callosities sometimes appear to form anterior protrusions when viewed directly from ventral. A somewhat oblique view is therefore best to determine if a true and substantial protrusion is present.

### Character 14: Nuchal callosity 5

Character definition: Lateral protrusion of nuchal callosity beyond the margins of the nuchal processes in skeletally mature specimens (new character): 0 = absent or minor (Fig. 5a, b, c, e); 1 = clearly present (Fig. 5d).

*Comments:* In the majority of pan-trionychids, the nuchal processes are wider than the nuchal callosity (state 0, Fig. 5a, b, c, e). This contrasts with the extant *Cyclanorbis senegalensis*, where the nuchal callosity is often broader than the underlying nuchal processes. This is best seen in ventral view, where the nuchal processes do not fully cover the nuchal callosity laterally (state 1; Fig. 5d). Statistical analysis highlights a weak positive correlation of this character with ontogeny, in that a lateral protrusion is more common in skeletally mature specimens (*SCC* = *0.21* for *Cyclanorbis senegalensis* only; see Additional file 2), but this value is likely too low, given that the available sample lacks true juveniles for this taxon. I, therefore, code each taxon by reference to the most skeletally mature individual.

### Character 15: Nuchal callosity 6

Character definition: Shape of the nuchal contact with costal 1 (modified from Jasinski et al., 2022, p. 96): 0 = suture absent or straight to curved (Fig. 2); 1 = suture wavy, due to the presence of “posterolateral bulges” (Jasinski et al., 2022, Fig. 3).

Comments: In the majority of pan-trionychids, the nuchal processes suturally contact the costal series posteriorly along a straight to curved suture, if at all. In a small number of fossil forms, this contact is developed sinuously due to the presence of posterolateral protrusions of the nuchal that lap onto the costals, as first noted by Jasinski et al. (2022). This character is not parsimony informative among extant trionychids, as it is developed in none. The character is retained, nonetheless, to help better resolve the fossil record of extinct forms.

### Characters 16 and 17: Suprascapular fontanelles 1 and 2

Initial observations: Posterior sutural contacts of nuchal callosity and suprascapular fontanelles (modified from Meylan, 1987, p. 18; Vitek, 2012, p. 88): 0 = posterior contacts of nuchal callosity absent (Fig. 4a); 1 = posteromedian contact of nuchal callosity present, suprascapular fontanelles, if present, remain laterally open (Fig. 4b); 2 = broad posterior contact present, suprascapular fontanelles absent (Fig. 4f, g, h); 3 = broad posterior contact interrupted by paired, circular suprascapular fontanelles (Fig. 4e); 4 = posterolateral contacts present, suprascapular fontanelles confluent (Fig. 4c, d).

Character 16, definition: Formation of suprascapular fontanelles during ontogeny (modified from Vitek, 2012, p. 88): 0 = nuchal callosity never establishes posterior contact with neurals or costals; 1 = nuchal callosity initially contacts neurals posteromedially to form two laterally open suprascapular fontanelles; 2 = nuchal callosity initially contacts costals lateromedially to form two medially confluent suprascapular fontanelles.

Character 17, definition: Closure of suprascapular fontanelles during ontogeny (modified from Meylan, 1987, p. 18): 0 = suprascapular fontanelles close early in ontogeny, at less than 30% of maximum adult body size; 1 = suprascapular fontanelles close mid ontogeny, at 30–70% of maximum adult body size; 2 = suprascapular fontanelles close late in ontogeny, at greater than 70% adult size; 4 = suprascapular fontanelles never or rarely close.

Comments: Many pan-trionychids exhibit paired fontanelles at the posterior contact of the nuchal callosity with the neurals and costals that are referred to as suprascapular fontanelles as they are located above the dorsal contact of the scapular processes with the shell (Fig. 4e). I note that suprascapular fontanelles form initially along one of two distinct pathways. The nuchal callosity typically originates at the midline, but lacks any sutural contacts with the neural column or costals (character 16, state 0; Fig. 4a). This condition is retained in the presumably adult holotype of the extinct *Perochelys lamadongensis* (Li et al., 2015). As the nuchal callosity expands, it either initially contacts the neural column posteromedially (character 16, state 1; Fig. 4b) or it initially contacts the costals posterolaterally to frame medially confluent suprascapular fontanelles (character 16, state 2; Fig. 4c, d). These conditions are retained in adults of the extinct *Axestemys latus and Axestemys quinni* (Gardner et al., 1995; Schmidt, 1945) and the extant *Dogania subplana*, respectively. The three states do not form a morphocline and should not be ordered.

Meylan (1987) noticed that suprascapular fontanelles of trionychids close at varying stages over the course of their ontogeny. He, therefore, developed a character that unites taxa in which fontanelles close at hatching, taxa in which fontanelles close in large adults only, and taxa that maintain open fontanelles throughout their ontogeny. I am not able to replicate the observation that some trionychid hatch with closed suprascapular fontanelles, as I am unaware of studies that sufficiently document this part of ontogeny for a larger sample of trionychids and as my sample of photographs only includes few hatchlings and juveniles. The study of Stoffert (1889) on the ontogeny of *Lissemys ceylonensis*, however, suggests that some trionychids may not form suprascapular fontanelles in the first place, but rather close up the existing space by having the initial posteromedian contact expand laterally. If the relative size of an individual is taken as a proxy for ontogeny, my large sample of subadult to adult trionychids with known dimensions nevertheless allows me to confirm that suprascapular fontanelles can close during all phases of ontogeny and that systematic differences exist between species. I, therefore, rephrase the original character of Meylan (1987) to unite turtles, in which suprascapular fontanelles never form or close early in ontogeny (less than 30% of maximum straight carapace length), mid-ontogeny (30–70%), late in ontogeny (greater than 70%), or rarely to never. These character states form a morphocline that can be ordered.

The two characters that I developed in regard to suprascapular fontanelles are logistically difficult to encode, as the scoring of each taxon must be extracted from observations made from the full sample of individuals. I, therefore, utilize one column in the matrix to score observations at the level of the individual and from which the coding of each taxon is then derived. This column must be deactivated when running the phylogenetic analysis, as it does not represent a true character.

### Character 18: Nuchal processes 1

Character definition: Width of nuchal processes (Fig. 5a, e) relative to the greatest width of the carapace in ventral view in skeletally mature specimens (modified from Meylan, 1987, p. 1): 3 = 30–40%; 4 = 40–50%; 5 = 50–60%; 6 = 60–70%; 7 = 70–80%. Specimens approaching a given threshold within 2 percentage points are scored polymorphic.

Comments: This is the third character that documents variation to the shape and proportions of the nuchal (see Character 10 above for a broader introduction). Although the width of the nuchal callosity (see Character 10 above) often corresponds to the width of the nuchal processes, the two measurements are independent, as the nuchal callosity is much broader than the underlying nuchal processes in some taxa (e.g., *Cyclanorbis senegalensis*) but much narrower in others (e.g., some representatives of the extinct *Axestemys*). Measurements for this character were taken in ventral view using ImageJ, as the width of the nuchal processes is often obscured by the nuchal callosity in dorsal view (Fig. 5a, e). The greatest width of the carapace includes the free rib tips. As a full gradation is apparent among extant trionychids from less than 30% to more than 60%, characters states were formed that evenly divide the spectrum into segments of 10 percentage points, but specimens were scored intermediate, if their measurements approach a given threshold within 2 percentage points. To allow this character to expand in the future, character states were labeled according to the interval they signify (e.g., character 3 encompasses measurements from 30–40%). Statistical analysis reveals a weak negative relationship in that larger individuals have proportionally narrower nuchal processes than smaller individuals (*SCC* = − *0.33*; see Additional file 2). The final scoring for each taxon, therefore, takes ontogeny into account while addressing variation and measurement uncertainty through the liberal use of polymorphism. The character states of this character form a morphocline.

### Character 19: Nuchal processes 2

Character definition: Anteroposterior depth of nuchal processes (new character): 0 = short, lateral processes of nuchal jointly form an anteroposteriorly narrow, recurved strut consisting of tightly packed subprocesses (Fig. 5e); 1 = long, lateral processes form laterally expanded combs that jointly give the nuchal processes the shape of a bow tie (Fig. 5a, b, c, d).

Comments: Significant variation is apparent in the anteroposterior depth of the nuchal processes (costiform processes of Meylan, 1987) among pan-trionychids. The nuchal processes either converge to form a single, rib like process that may consist of a small number of tightly packed subprocesses, such as in *Chitra* spp. (Fig. 5e) or the fossil *Hutchemys walkerorum* (Jasinski et al., 2022), or a broad, bow-tie-like element consisting of laterally expanding fans made up of many subprocesses (Fig. 5a, b, c, d). No intraspecific variation is apparent for this character.

Although this character appears to be naturally discrete among extant trionychids, I presume that rich sampling of fossil trionychids will reveal intermediates. However, as the nuchal processes are often difficult to distinguish from the overlying nuchal callosity in fossils, I refrain from developing a morphometric character, as it would not be possible to implement it rigorously using fossils. In that regard, scoring juveniles is advantageous, as the nuchal processes of many taxa are not yet covered by the nuchal callosities.

### Character 20: Nuchal processes 3

Character definition: Shape of nuchal processes (modified from Meylan, 1987, 2): 0 = lateral processes form a tight comb (Fig. 5a, b, d, e); 1 = comb laterally split into anterior and posterior processes separated by a distinct gap (Fig. 5c).

*Comments:* This character follows Meylan (1987) by uniting the comb-like lateral processes seen in the vast majority of pan-trionychids (state 0; Fig. 5a, b, d,), as opposed to the split comb seen in many cyclanorbines (state 2; Fig. 5c). No variation is apparent within any given species.

### Character 21: Nuchal processes 4

Character definition: Placement of nuchal processes relative to costal I (new character): 0 = nuchal processes situated anterior and dorsal to costal I (Fig. 5a, b, e); 1 = nuchal processes slightly underlap the anterior margins of costal I (Fig. 5d); 2 = nuchal processes strongly underlap anterior margins of costal I (Fig. 5c).

Comments: The nuchal processes of the vast majority of pan-trionychids form a tight comb that is situated anterior and slightly dorsal to costal I. As a result, the distal aspects of the nuchal processes are partially covered by costal I, typically its rib, in ventral view (state 0; Fig. 5a, b, e). In cyclanorbines, the nuchal processes either slightly (state 1; Fig. 5d) or strongly (state 2; Fig. 5c) underlap costal I. This costal is therefore partially covered by the nuchal in ventral view. This character forms a morphocline. No variation is apparent within each taxon.

### Character 22: Dorsal vertebra

Character definition: Location of the articulation of the cervical vertebral column with the dorsal vertebral column relative to the nuchal (reworded from Meylan, 1987, 3): 0 = in the posterior third of the nuchal (Fig. 5c); 1 = in the central third of the nuchal (Fig. 5a, b, d); 2 = in the anterior third of nuchal (Fig. 5e).

Comments: The highly mobile articulation between the cervical and dorsal column is situated below the nuchal in pan-trionychids, but variation is apparent to its exact placement. Meylan (1987) noted that the joint is either situated below the anterior, middle (Fig. 5a), or posterior thirds of the nuchal and created a character that captures this variation. However, the first dorsal vertebra disarticulates easily after death (Fig. 5b, c, d, e), so, as phrased by Meylan (1987), this character cannot be scored for many specimens, especially fossils. Fortunately, the location of the cervical/dorsal joint fully correlates with paired depression on the ventral side of the nuchal that provide space for the capsule that surround the joint, either being located in the posterior (Fig. 5c, black arrows), central (Fig. 5b, d, black arrows), and anterior third of the median length of the nuchal (Fig. 5e, black arrows). This character can thus be scored using either criterion. The three available character states form a morphocline that can be ordered. Only extremely minor levels of polymorphism are observed among the available sample of extant trionychids.

### Character 23: Preneural

Character definition: Preneural (modified from Meylan, 1987, 4): 0 = absent (Figs. 2a, c; 4a, c, d, e, f); 1 = present (Figs. 2b, d; 4b, g, h).

Comments: Fossil and recent pan-trionychids variously exhibit one or two neural elements between the first pair of costals (Figs. 2, 4). Hay (1904) introduced the term "preneural" to refer to the anterior of the two elements, but did not comment explicitly on its homology. The novel term “preneural”, nevertheless, suggests that he believed it to be neomorphic relative to other turtles. Over the course of the last decades, the preneural of pan-trionychids has been argued to be the homolog of the first neural of other turtles (Meylan, 1987), a neomorphic structure unrelated to the neurals (Cherepanov, 1995), or a true first neural not homologous to the first neural of other turtles (Kordikova, 2002). In all cases, the relationships of the neurals to the underlying vertebral column was used as a source of topological evidence, yet, the results are highly contradictory. After reviewing all of the available data, I here conclude that the anterior element is a neomorphic bone best termed "preneural." My rationale is outlined below (see Discussion). To avoid confusion, I address this element, unless noted otherwise, as the preneural.

The presence or absence of the preneural is remarkably stable for each species within the available sample. Rare exceptions include NHMB 2396 *Lissemys ceylonensis* (Fig. 4g), which possesses a strongly reduced preneural, and CUMZ T37 *Amyda ornata* and CUMZ T53 *Amyda ornata*, which exhibit small, irregularly shaped elements in the position of a preneural. Cherepanov (1995) reported similar exceptions for *Pelodiscus* sp. and *Trionyx triunguis*, but I doubt the veracity of the latter claim, as the depicted morphology resembles *Nilssonia* spp.

### Character 24: Neurals 1

Character definition: Number of neurals, not including the preneural, but including neural gaps (modified from Joyce et al., 2016, p. 12; Meylan, 1987, p. 14; Vitek, 2011, p. 12): 0 = neurals absent; 1 = 1 neural present; 2 = 2 neurals present; 3 = 3 neurals present; 4 = 4 neurals present; 5 = 5 neurals present; 6 = 6 neurals present; 7 = 7 neurals present; 8 = 8 neurals present; 9 = 9 neural present.

**Fig. 6:**
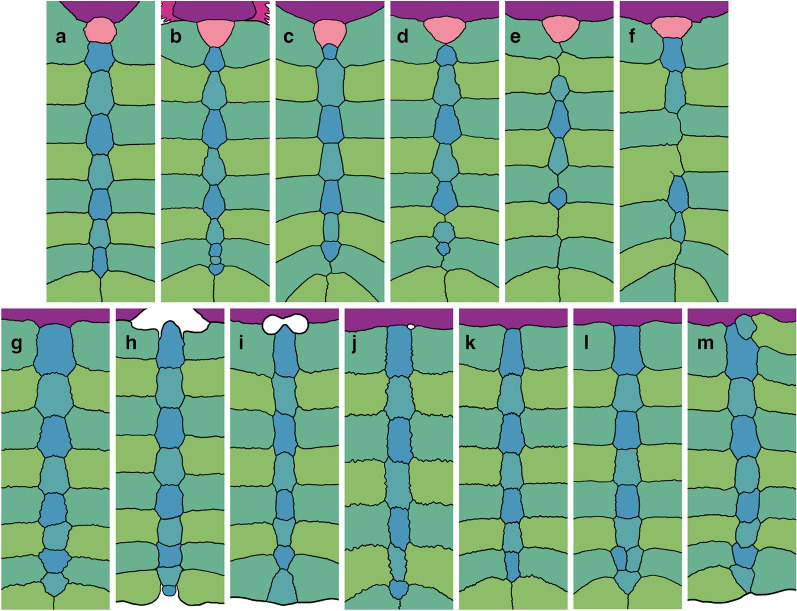
Line drawings of the neural column of select carapaces in dorsal view highlighting variation in bone development: **a** *Cycloderma aubryi* (NMB uncat.), **b** *Cyclanorbis elegans* (BMNH 1954.1.14.3), **c** *Hutchemys rememdium* (YPM-PU 16795), **d** *Cyclanorbis elegans* (PCHP 8215), **e** *Cyclanorbis senegalensis* (MTD 38635), **f** *Cyclanorbis elegans* (NMW 1439), **g** *Chitra chitra* (PCHP 4937), **h** *Dogania subplana* (FMNH 224111), **i** *Apalone mutica* (PCHP 2655), **j** *Apalone ferox* (AMNH 57384), **k** *Pelodiscus* sp. (PCHP 3425), **l** *Pelodiscus* sp. (PCHP 3422), **m** *Amyda ornata* (CUMZ T42). Images are not to scale. Colors are used to highlight the anatomical systems labelled in Fig. 1

Comments: I here follow Vitek (2011) by utilizing discrete character states and ease scoring by utilizing the same number for the character state as the number of neurals defining the given character state (i.e., character state “7” is defined as seven neurals being present; Fig. 6a, c, j). The observed neural counts in the available sample range all the way from one (scored as “1”) to nine (scored as “9”).

Meylan (1987) and Gardner and Russell (1994) documented a large amount of intraspecific variation to the count of neurals among pan-trionychids. To address this variation, Meylan (1987) developed character states defined by the presence of polymorphism (e.g., 0 = 8 neurals; 1 = 7 or 8 neurals; 2 = 7 neurals). This approach was certainly novel, at least within the literature pertaining to turtle systematics, by explicitly addressing the problem of polymorphism, but I here note three problems with this approach. First, as Meylan’s (1987) character does not quantify polymorphism, disproportionate weight can be given to a single individual with an unusual neural count. Second, as much variation appears to be caused by developmental irregularities (Fig. 6), not genetic factors, it is unclear if species are coded polymorphic based on deformed individuals. And third, it is difficult to score fossil taxa based on single individuals. Vitek (2011) addressed the latter issue by converting the scoring of Meylan (1987) into discrete character states, but the first two issues remain unresolved to date.

While scoring each individual, I took note of the presence of abnormalities in a given specimen, including unusual or asymmetric costal counts (e.g., 7 or 9, with exception of representatives of *Apalone*, which regularly exhibit the presence of only 7 costals; Fig. 6f, m), the presence of split neurals (i.e., the presence of two neurals in the space normally filled by a single neural; Fig. 6b, l), the presence of fused neurals (i.e., unusual, polygonal elements spanning two costals; Fig. 6k), or the absence of neurals (i.e., the absence of a neural element in the space normally filled by a neural; Fig. 6d, e). Highly abnormal individuals were disregarded when establishing the scoring for each species (Fig. 6f, m). Gaps were counted, to avoid incorrectly grouping individuals with non-homologous element (e.g., the specimen in Fig. 6e is scored as possessing five neurals [i.e., neurals associated with neurals I–V], even if the first is not developed). Fused neurals were counted separately. Finally, species were only coded polymorphic if more than 20% of well-formed individuals display the second character state (i.e., more than 1 individual out of 5). The available character states form a morphocline that can be ordered.

In contrast to Meylan (1987), the preneural is not included in the count of neurals. My scorings therefore systematically differ from those of Meylan (1987) by one.

### Character 25: Neurals 2

Character definition: Neural reversal (new character): 0 = absent, all neurals are hexagonal and have posteriorly short sides (Fig. 6a, d, e); 1 = present, a square neural is present that separates more anterior hexagonal neurals with posteriorly short sides from more posterior hexagonal neurals with anteriorly short sides (Fig. 6b, c, h, i, l).

Comments: The neural column of pan-trionychids either consists of a row of hexagonal elements with short posterior sides (state 0; 6a) or shows a "reversal" in that the anterior elements are hexagonal with short posterior sides, while the posterior ones are hexagonal with short anterior sides. The transition is bridged by a square neural (state 1; Fig. 6c, h, i, l). This character is not only affected by the many of the abnormalities I list for the previous character (Fig. 6b, d, e, k), but also through the presence of right/left asymmetries and scoliosis to the vertebral column, which can drag reversals across numerous elements (Fig. 6f, g, i, j, m). My coding for each taxon is, therefore, mostly undertaken by reference to regularly formed individuals. Abnormal specimens and polymorphism were addressed as in the previous character (see Character 24 above). I see no evidence that the presence of a neural reversal changes over the course of ontogeny. As above, my scorings differ systematically from those of Meylan (1987) as I do not include the preneural in the neural count.

### Character 26: Neural 3

Character definition: Placement of neural reversal (modified from Joyce et al., 2016, p. 15; Meylan, 1987, p. 17; Vitek, 2011, p. 76, 77): X = reversal located at neural X (i.e., 4 = reversal at neural IV).

Comments: Meylan (1987) developed two characters that utilize variation to the location of the neural reversal (i.e., placement of the square neural that reverses neural orientation) and variation in the amount of neural reversal variation, including use of character states defined by the presence of polymorphism. I here present a single character that uses discrete character states only. Scoring is eased by utilizing the same number for the character state as the number of the neural at which the reversal occurs (i.e., character state “6” is defined as the reversal being located at neural VI; Fig. 6c, h). The variation found in the available sample ranges from a reversal at neural I (scored as “1”) to neural VIII (scored as “8”). Particularly irregular specimens were scored “9” to visually highlight abnormalities, but this scoring was disregarded when establishing the coding of species. Asymmetric reversals found within an individual were initially scored polymorphic (i.e., the scoring for the right and left side), while taking note of this abnormality (Fig. 6g). In rare cases, the reversal can be drawn across several neurals, which is also noted through the use of polymorphism (Fig. 6j, m). Specimens that lack a reversal were scored non-applicable. Highly irregular specimens, including asymmetric specimens, were disregarded when establishing the scoring for a species. In addition, species were only coded polymorphic if more than one in five regularly formed individuals (i.e., more than 20%) displayed the second character state. I see no correlation between ontogeny and the placement of the neural reversal. This character forms a morphocline that can be ordered. As with the previous character, my scorings differ systematically from those of Meylan (1987) as the preneural is not included in the neural count.

### Character 27: Neural 4

Character definition: Octagonal neurals (modified from Joyce et al., 2009, p. 69): 0 = absent (Fig. 6a, b, d, e, g, h, j, k, l); 1 = present (Fig. 6c).

Comments: The neurals of pan-trionychids are generally square or hexagonal in outline (i.e., have four or six contacts with the surrounding elements; state 0; Fig. 6a, b, d, e, g, h, j, k, l). Joyce et al. (2009) developed a character that pertains to the presence of an octagonal neural (i.e., have eight contacts with the surrounding elements), as developed among others in the Paleocene *Hutchemys rememdium* (state 1; Fig. 6c). This arrangement was found asymmetrically on one side of the shell (i.e., four contacts on one side, but only two or three on the other side) in slightly more than 20% of individuals of *Apalone ferox*, *Apalone spinifera*, *Cylanorbis senegalensis*, and *Cycloderma frenatum* available in the sample, all of which were coded polymorphic. In contrast to Joyce et al. (2009), this character is not restricted to a particular neural being octagonal, but it is the second in all recorded instances. There is no evidence that the presence of octagonal neurals is influenced by ontogeny. This character is parsimony uninformative among extant trionychids, as no taxon unambiguously exhibits the derived character state. This character is retained, nonetheless, as numerous fossil forms consistently exhibit octagonal neurals.

### Character 28: Neural 5

Character definition: Neural proportions (new character): 0 = neurals elongate, much longer than wide (Fig. 6a–f, h–m); 1 = neurals broad, nearly as wide as long (Fig. 6g).

Comments: In the vast majority of pan-trionychids, the neurals are elongate elements that are significantly longer than wide (state 0; Fig. 6a–f, h–m). This differs in a small group of fossil and living pan-trionychids, including *Chitra* and *Pelochelys* spp., where the neurals are notably broad, almost as wide as long (state 1; Fig. 6g). As this character lacks naturally discrete character states, specimens with intermediate morphologies are herein scored polymorphic. Species, on the other hand, are only coded polymorphic, if one of the two character states occurs in at least 20% of the population.

### Character 29: Neural 6

Character definition: Reduction of neurals I–VII (new character): 0 = absent, neurals I–VII form a contiguous sequence of elements that fully separate the costals (Fig. 6a–c, g–m); 1 = present, neurals I–VII form a discontinuous series of elements that allows for the formation of secondary intercostal contacts (Fig. 6d, e, f).

Comments: The neural column of pan-trionychids is known to exhibit a particularly large amount of intraspecific variation (Gardner & Russell, 1994; Meylan, 1987; Pritchard, 1988). The available sample documents right/left asymmetries, duplicate elements, a posterior reduction of the neural column, and reduction of neurals within the column. Meylan (1987) developed a character that captures variation to the number of developed neurals (also see Character 24 above), but a literal application of this character, I believe, can lead to spurious results. For instance, a contiguous neural column consisting of five well-formed neurals differs systematically from a neural column consisting of highly reduced neurals I, III, IV, VI, and VII, but the two will score the same, as both consist of five neural elements. To address this issue, I created a neural character that counts neural elements, but the count excludes neural duplicates while including gaps (also Character 24 above). The present character addresses the remaining variation, which is the presence of reduced to absent neurals within the column, which can also be conceptualized as the presence of secondary intercostal contacts (state 1; Fig. 6d, e, f). In extant taxa that display a reduction of the interneural contacts, such as *Cyclanorbis senegalensis*, I record large amount of variation ranging from no reduced neurals to five reduced neurals. I, therefore, grouped all types of reductions, regardless of their frequency or position within the column, into a single derived character state. As with other neural characters, I only coded taxa as polymorphic, if the second character state occurs in 20% of individuals. When establishing the coding for a species, I ignore occasional secondary intercostal contacts that exist between neural VII and VIII, as this element is typically small, when present.

### Character 30: Costal 1

Character definition: “Splitting” of costals along their distal margins in skeletally mature specimens (reworded from Joyce et al., 2009, p. 70): 0 = absent; 1 = present.

Comments: The costals of most trionychids either taper towards their margins or end bluntly (state 0). In a small group of pan-trionychids, especially plastomenids, but also extant *Apalone ferox* and *Pelochelys* spp., the outer margins of the costals expand through the formation of flat projections made up of surficial and visceral bony tissue (state 1). As a result, the distal margin of these bones is decorated by a sulcus and appears to be “split” (see Joyce et al., 2009, Fig. 2e). The splitting may occur over the full length of the costal margins, as in *Hutchemys* spp., or may restricted to a portion of the circumference, as in *Pelochelys* spp. As the costals grow significantly along their distal margins during ontogeny, this character shows a moderate correlation with ontogeny (*SCC* = *0.62* for taxa with split costals; *SCC* = *0.54* for *Pelochelys spp*; *SCC* = *0.43* for *Apalone ferox*; see Additional file 2). Each species is therefore coded by reference to the most skeletally mature individuals.

### Character 31: Costal 2

Character definition: Relationship of costal I to the nuchal callosity (modified from Jasinski et al., 2022, p. 97): 0 = costal I positioned posterior to nuchal callosity (Fig. 4a, b, e, f); 1 = costal I laterally surrounds less than half of the midline length of the nuchal (Fig. 4d); 2 = costal I laterally surrounds more than half of the midline length of the nuchal (Fig. 4c, g, h).

Comments: In the vast majority of trionychids, the anterior margin of costal I runs transversely across the shell and the costals are therefore located posterior to the nuchal (state 0; Fig. 4e, f). In some taxa, the distal margins of costals I expand towards the anterior during ontogeny and thereby either partially (less than 50% of the midline depth of the nuchal; state 1; Fig. 4d) or mostly (more than 50% of the midline depth of the nuchal; state 2; Fig. 4c, g, h) surrounds the nuchal laterally. This character is easiest scored by running a line between the anterolateral tips of the right and left costal I and observing where it crosses the midline of the nuchal (Fig. 4d, red line). As this character relies on non-discrete character states, individuals displaying intermediate morphotypes were liberally scored as polymorphic. As the nuchals expand anterolaterally during growth, a weak correlation with ontogeny can be found for this character in the available data set of extant trionychids in that more of the nuchal is surrounded by the costals in more skeletally mature individuals (*SCC* = *0.48*; see Additional file 2). Each species is therefore coded by reference to the mostly skeletally mature individuals. The available character states form a morphocline that can be ordered.

### Character 32: Costal 3

Character definition: First pair of costals with midline contact posterior to the neural column (modified from Meylan, 1987, p. 16; Vitek, 2011, p. 14): 5 = costal V; 6 = costal VI (Fig. 6e); 7 = costal VII (Fig. 6c, d, j); 8 = costal VIII (Fig. 6a, b, g, k, l); 9 = none (Fig. 6h, i).

Comments: Meylan (1987) developed a character that addresses variation to the most anterior midline contact of the costal elements. To ease scoring, I here rephrased the character definition to have the numbering of character states correspond to the most anterior costal to have a midline contact (e.g., character state “7” is defined as the most anterior costal contact being located at costal VII; Fig. 6c, d), with exception of taxa that lack a midline contact, which are scored as “9” (Fig. 6h, i, m). This approach results in a morphocline that can be ordered. As some trionychids have irregular, supernumerary contacts of the costals within the neural column (see Character 29 above), this character is restricted to midline contacts posterior to the last neural. The most anterior costal to have a midline contact correlates only partially with the number of neurals as it is possible for two taxa to have seven neurals, but to vary in the presence of a midline contact of costal VII (Fig. 6a versus c). This character is, therefore, retained as independent. As with the neural count (see Character 24 above), the scoring for each species was achieved by disregarding specimens with obvious irregularities. In addition, species were only coded polymorphic, if more than 20% of individuals display a second character state.

### Character 33: Costal 4

**Fig. 7:**
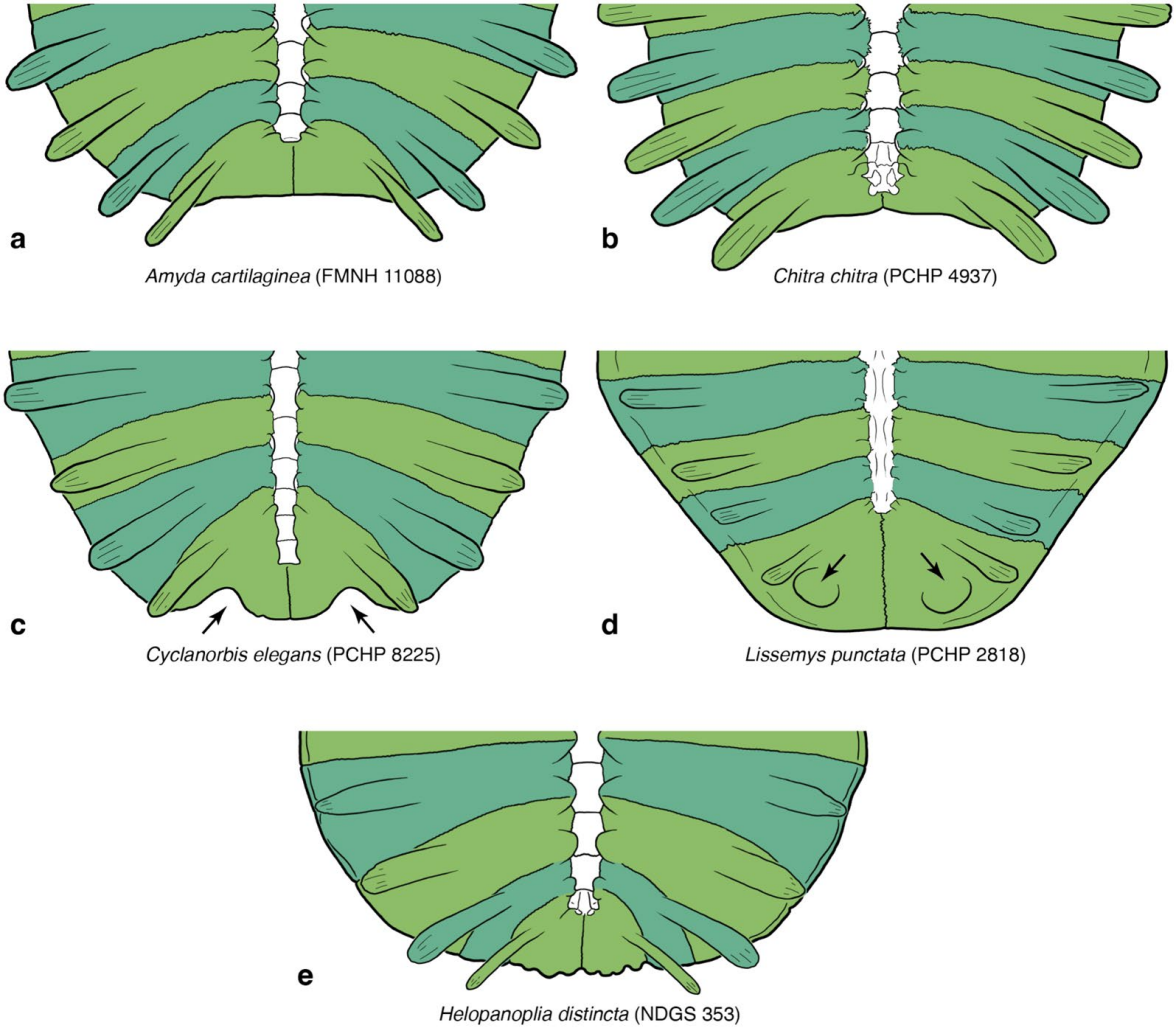
Line drawings of the posterior part of select carapaces in ventral view highlighting variation in bone development: **a** *Amyda cartilaginea* (FMNH 11088), **b** *Chitra chitra* (PCHP 4937), **c** *Cyclanorbis elegans* (PCHP 8225), **d** *Lissemys punctata* (PCHP 2818), **e** *Helopanoplia distincta* (NDGS 353). Images are not to scale. Colors are used to highlight the anatomical systems labelled in Fig. 1. Black arrows highlight sinuous shell margins and depressions on the underside of costals

Character definition: Depression for ilia on the visceral side of costals VIII in skeletally mature individuals (modified from Meylan, 1987, p. 21): 0 = absent, the ilium is not roofed by the shell (Fig. 7a, b, e); 1 = depressions only partially formed, the posterior shell margin is sinuous (Fig. 7c); 2, present, ilia are fully roofed by the shell, depressions are apparent on the underside of costals VIII (Fig. 7d).

Comments: In most pan-trionychids, costals VIII are short and the pelvis is therefore not roofed by bone (state 0; Fig. 7a, b, e). In some pan-trionychids, costals VIII are expanded posteriorly and the pelvis is partially (state 1) roofed. As the area just above the ilia does not ossify, however, the posterior margin of the shell has a sinuous outline (Fig. 7c, black arrows). In other pan-trionychids, the costals VIII fully roof the pelvis resulting in ilial depressions on the underside of the shell (Fig. 7d, black arrows). Although analysis of all taxa that show variation to this characteristic yields no relationship with ontogeny (*SCC* = *0.08*; see Additional file 2), analysis of individual taxa typically yields moderate relationships with ontogeny, in that more skeletally mature individuals have more strongly roofed ilia (*SCC* = *0.45* for *Cyclanorbis elegans*; *SCC* = *0.28* for *Cyclanorbis senegalensis*; *SCC* = *0.57* for *Cycloderma aubryi*; *SCC* = *0.56* for *Lissemys punctata*; *SCC* = *0.87* for *Lissemys scutata*; see Additional file 2). As these numbers almost certainly are underestimates, as the affected species only poorly sample juveniles, I coded taxa by reference to the most skeletally mature individuals.

### Character 34: Costal 5

Character definition: Surface proportion of costals VII + VIII relative to costals VI (modified from Meylan, 1987, 8): 0 =  < 75%, costals VIII typically very reduced to absent; 1 = 75–150%; 2 = 150–225%; 3 =  < 225–300%; 4 =  > 300%. Specimens approaching a given threshold within 5 percentage points are scored polymorphic.

**Fig. 8:**
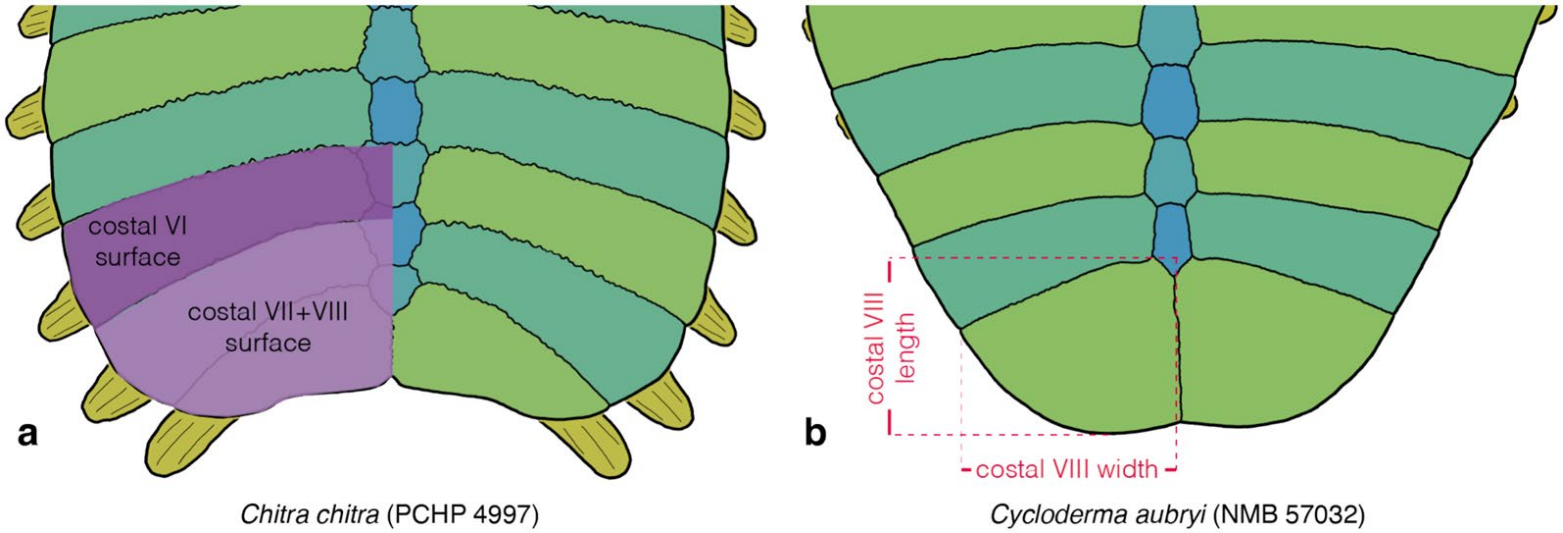
Line drawings of the posterior part of select carapaces in dorsal view highlighting variation in bone development: **a** *Chitra chitra* (PCHP 4997), **b** *Cycloderma aubryi* (NMB 57032). Images are not to scale. Colors are used to highlight the anatomical systems labelled in Fig. 1

Comments: The posterior costals, especially costals VII and VIII, can vary significantly in size relative to the more anterior costals. I here convert Meylan’s (1987) original character pertaining to the reduction or loss of costals VIII into a morphometric character with five character states, as this allows utilizing a far greater band width of the apparent variability. All surface measurements used herein were obtained from photographs using ImageJ (Fig. 8a). As the size of the posterior costals is greatly distorted in straight dorsal views of the carapace, especially in highly domed taxa, measurements were taken based on pictures taken perpendicular to the pygal area. This in return precludes using the entire carapace as the standard against which costal size is measured. As there is much variation to the presence and relative size of the posterior neurals (see Characters 24–29 above), the surface of the adjacent neurals was included in the measurement for each costal. This character, therefore, approximates to the entire neural/costal segment, not just the costals. To average minor right/left asymmetry, measurements were obtained, when possible, for both sides at once and then divided by two. I initially collected separate data for the size of costals VII and costals VIII relative to costals VI, but statistical analysis demonstrates that these measurements are strongly correlated with one another (*SCC* = *0.78*; see Additional file 2). I, therefore, utilize the ratio between the combined size of costals VII/VIII versus costals VI. Specimens that approach thresholds values within 5% points were scored intermediate. The vast majority of specimens that lack costals VIII, Meylan’s (1987) original character, have a value below 80% and are, therefore, either scored 0 or polymorphic.

Further statistical analyses reveal a weak (*SCC* = *0.22*; see Additional file 2) correlation with ontogeny for the size of costals VII + VIII relative to costals VI. Species were therefore only coded by reference to the most skeletally mature individuals if a clear size trend is apparent. If a clear trend is not apparent, species were coded polymorphic. The character states of this character form a morphocline that can be ordered.

### Character 35: Costal 6

Character definition: Width to length proportion of costal VIII (modified from Joyce & Lyson, 2011, p. 83): 0 =  < 75%; 1 = 75–150%; 2 = 150–250%; 3 =  > 250–350%; 4 =  > 350%. Specimens approaching a given threshold within 5 percentage points are scored polymorphic.

Comments: The size and proportion of costals VIII vary greatly across pan-trionychids. In addition to varying in size relative to the rest of the carapace (see Character 34 above), much diversity is apparent in regard to the width of costals VIII relative to the width of the carapace and the width to length proportions of costals VIII. I initially took all three sets of measurements, as their interrelationships were not apparent a priori. The width of costals VIII relative to the width of the carapace is moderately to strongly correlated with the size of costals VII + VIII relative to costals VI (*SCC* = *0.63, 0.76*, and *0.75, respectively*; see Additional file 2), but the width-to-length ratio of costal VIII negatively correlates only weakly with the same three measurements (*SCC* = − *0.31*, − *0.49*, and − *0.47*, respectively; see Additional file 2). To avoid developing correlated characters, I, therefore, only utilize the width-to-height ratio of costal VIII as a second character pertaining to the shape and size of this element.

All measurements were obtained using ImageJ based on photographs taken perpendicular to the pygal area, as the shape of costal VIII can be greatly distorted in dorsal view, especially in taxa with a highly domed carapace (Fig. 8b). To average minor right/left asymmetry, the width of the costal was measured between the most distal points of contact between costals VII and costals VIII and divided by half. Furthermore, as the presence and size of neurals influence the midline length of the costals, the length was measured along the midline from the most anterior level of costals VIII to their posterior margins along the midline (Fig. 8b). Specimens and taxa with particularly small costals VIII were scored inapplicable (see Character 34 above), as highly reduced costals VIII typically have highly irregular shapes. Specimens lacking costals VIII were scored inapplicable. Taxa that have values that approximate the threshold value between two character states within 5 percentage points are scored polymorphic. The shape of costal VIII negatively correlates only weakly with ontogeny (*SCC* = − *0.38*; see Additional file 2). As this is certainly an underestimate, as my sample of juveniles is minimal, I at least coded all taxa by reference to the most skeletally mature individual. Individuals with apparent abnormalities were disregarded. This character forms a clear morphocline that can be ordered.

### Character 36: Costal 7

Character definition: Lateral embayment to the margin of costal V (modified from Joyce et al., 2009, 71): 0 = absent (Fig. 2); 1 = present (Jasinski et al., 2022; Fig. 3; Joyce et al., 2009; Fig. 5).

Comments: The lateral margin of the costals of all extant trionychids form a wavy to smooth carapacial margin. In a small number of fossil forms, a distinct embayment is developed along the margin of costal V only giving the carapace a lightly waisted appearance in dorsal view. Jasinski et al. (2022) suggested expanding this character into a morphocline with two derived character states, but as only few fossil forms exhibit this characteristic and differences are minor, I retain the original definition of Joyce et al. (2009). This character is non-informative for the present set of extant trionychids, but is retained to help resolve the relationships of fossil forms.

### Character 37: Costal ribs 1

Character definition: Development of free dorsal rib ends in skeletally mature individuals (Joyce & Lyson, 2017, p. 94): 0 = all costal ribs end free (i.e., are not dorsally covered by costal callosities, Fig. 7a, b); 1 = only costal ribs VII and VIII end free (Fig. 7e); 2 = all costal rib ends are dorsally fully, or nearly fully covered by the costal callosities (Fig. 7c, d).

Comments: In the majority of trionychines, the costal callosities cover the majority of the dorsal ribs, but all dorsal ribs nevertheless end free (i.e., are not covered dorsally by the costal callosities; state 0; Fig. 7a, b). In contrast, the dorsal ribs of most skeletally mature cyclanorbines are fully covered by the costal callosities (state 2; Fig. 7c, d). An intermediate is apparent in some fossil turtles, such as the Maastrichtian *Helopanoplia distincta* (Joyce & Lyson, 2017), where costal ribs I–VI are fully covered by the costal callosities, but costal ribs VII and VIII end free (state 1, Fig. 7e). This character is best observed in ventral view, as the distal rib ends are sometimes hidden in dorsal view. Special care must also be accorded to the nature of the rib ends, as these are damaged in many osteological and fossil specimens. The three herein formulated states do not form a morphocline so this character should therefore not be run ordered. As a result, it is not possible to test for a correlation with ontogeny, but as the costals universally expand in size while growing, it is apparent that this character exhibits a strong ontogenetic signal. Species are therefore coded by reference to the most skeletally mature individuals.

### Character 38: Costal ribs 2

Character definition: Overall width of dorsal ribs (new character): 0 = broad (Fig. 7a, b, c, e); 1 = distinctly narrow (Fig. 7d).

Comments: Pan-trionychids are notable relative to most other groups of turtles in that the dorsal ribs are particularly broad and flat (state 0; Fig. 7a, b, c, e). The ribs, therefore, cover more than one third of the costal callosities in visceral view. However, in a select group of pan-trionychids, especially cyclanorbines, all dorsal ribs are greatly reduced in size (state 1; Fig. 7d). As a full gradation is apparent between these two character states, individuals with intermediate morphology are scored liberally polymorphic (0/1). Statistical analysis shows no correlation of this character with ontogeny (*SCC* = *0.12* for *Cyclanorbis senegalensis*; SCC = *-0.13* for *Lissemys punctata*; see Additional file 2), so taxa are coded by reference to the dominant pattern. As the posterior ribs of many pan-trionychids are reduced in width relative to the more anterior ones (see Character 39 below), this character should mostly be scored by reference to the anterior six costal ribs. Although I herein otherwise prefer developing quantitative character, this character is left qualitative, as the width of the ribs is difficult to ascertain with confidence in many specimens.

### Character 39: Costal ribs 3

Character definition: Size of costal rib VIII (new character): 0 = costal rib VIII as broad as the remaining costal ribs (Fig. 7b, d); 1 = costal rib VIII reduced in width relative to the remaining ribs (Fig. 7c); 2 = costal rib VIII much slimmer than the remaining costal ribs (Fig. 7a, e).

Comments: In most pan-trionychids, all eight costal ribs have similar proportions to one another (state 0; Fig. 7b), but in some trionychines costal rib VIII (= dorsal rib IX) is partially (state 1, Fig. 7c) or strongly (state 2; Fig. 7a) reduced in width relative to the more anterior costal ribs. In many taxa where costal rib VIII is strongly reduced, a reduction is apparent to costal rib VII as well, which aids in distinguishing states 1 and 2. This character scores relatively consistent across most taxa. Those taxa that show variability exhibit a weak negative correlation at most with ontogeny, in that this characteristic is less prevalent in more skeletally mature specimens (*SCC* = *-0.48*; see Additional file 2). If a clear pattern emerges for any given species, this character is, therefore, coded by reference to the most skeletally mature individual. This character forms a morphocline that can be ordered.

### Character 40: Neomorphic plastral callosities

**Fig. 9:**
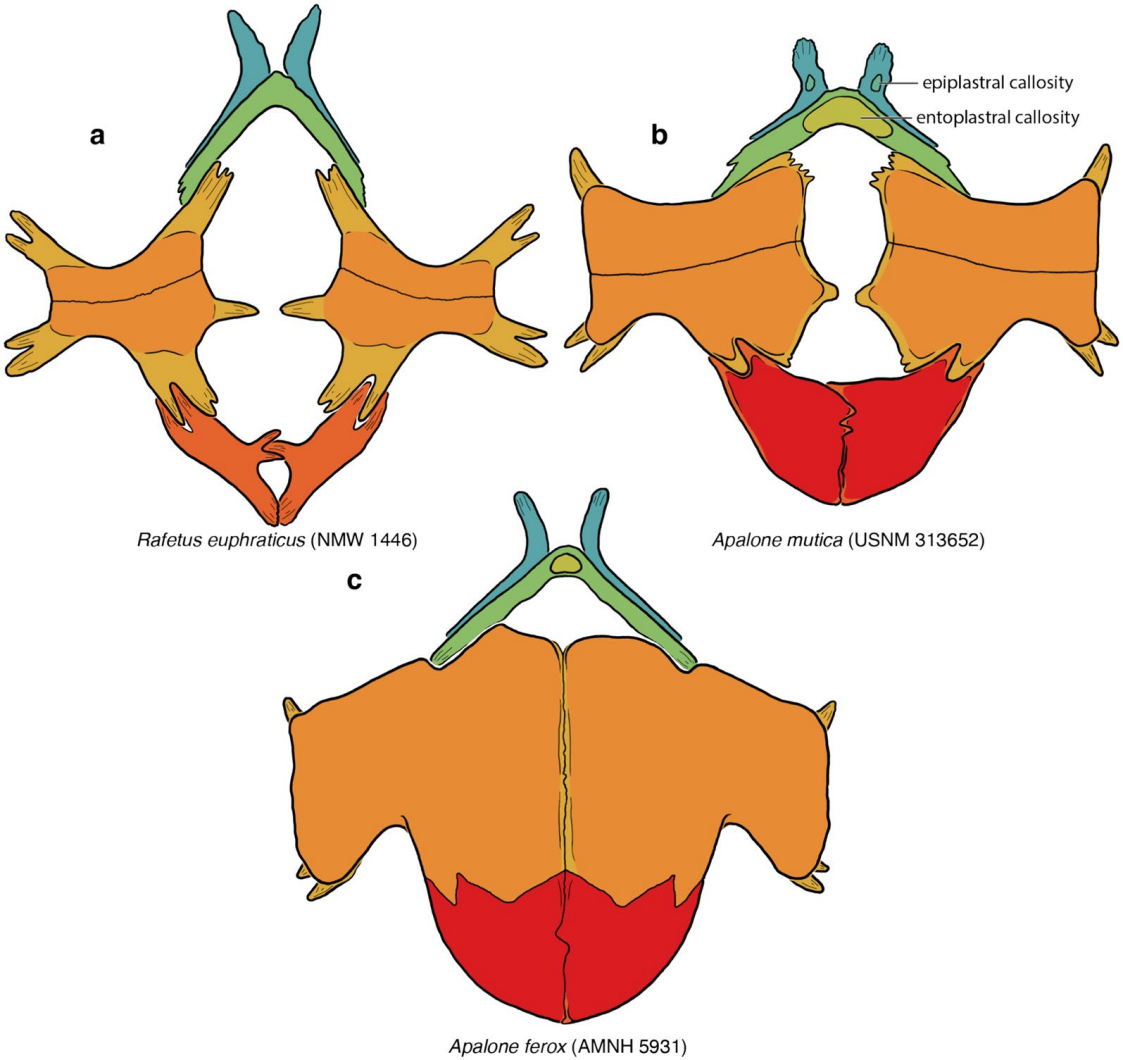
Line drawings of select plastra in ventral view highlighting variation in bone development: **a** *Rafetus euphraticus* (NMW 1446), **b** *Apalone mutica* (USNM 313652), **c** *Apalone ferox* (AMNH 5931). Images are not to scale. Colors are used to highlight the anatomical systems labelled in Fig. 1

**Fig. 10:**
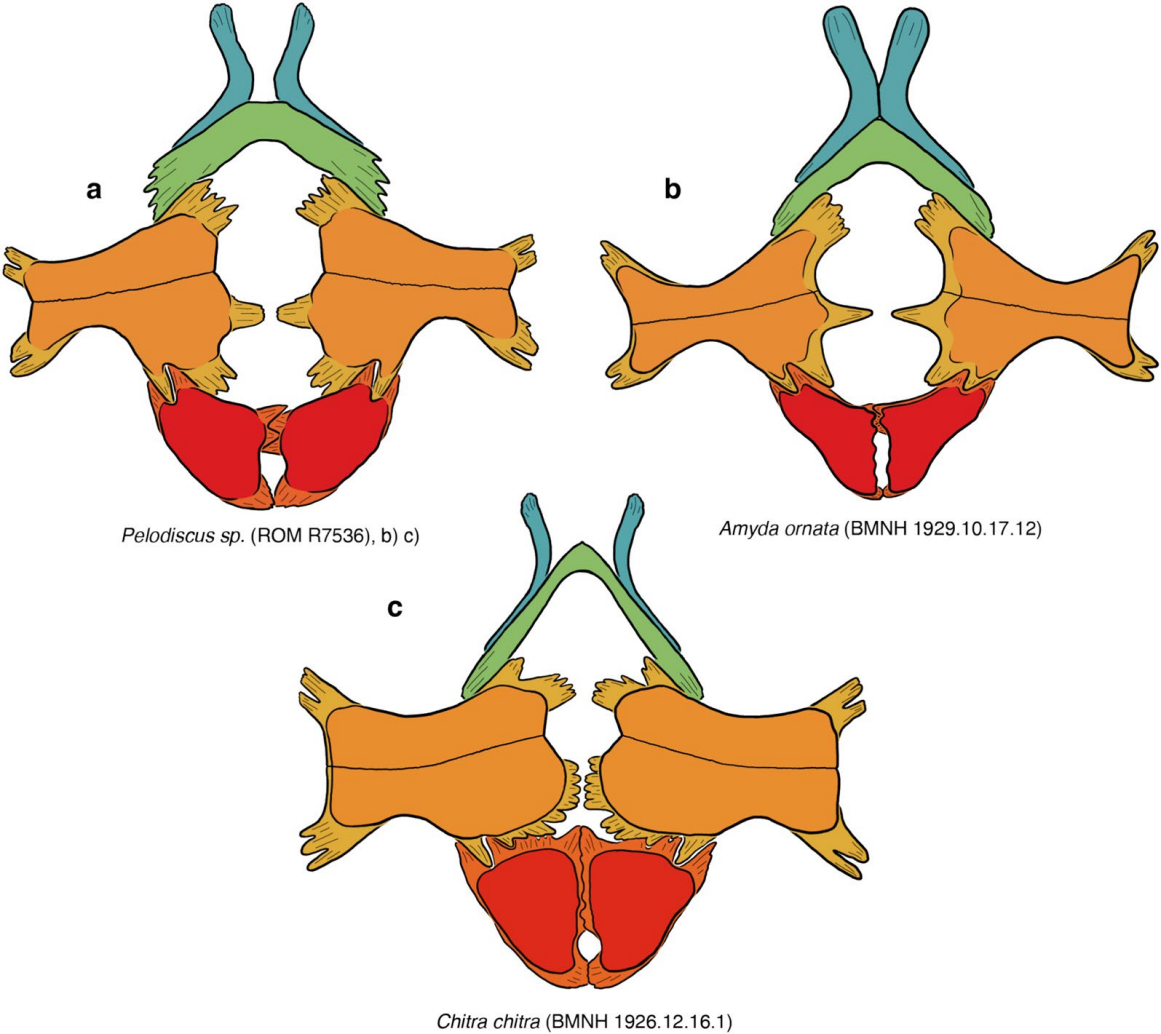
Line drawings of select plastra in ventral view highlighting variation in bone development: **a** *Pelodiscus* sp. (ROM R7536), **b** *Amyda ornata* (BMNH 1929.10.17.12), **c** *Chitra chitra* (BMNH 1926.12.16.1). Images are not to scale. Colors are used to highlight the anatomical systems labelled in Fig. 1

**Fig. 11:**
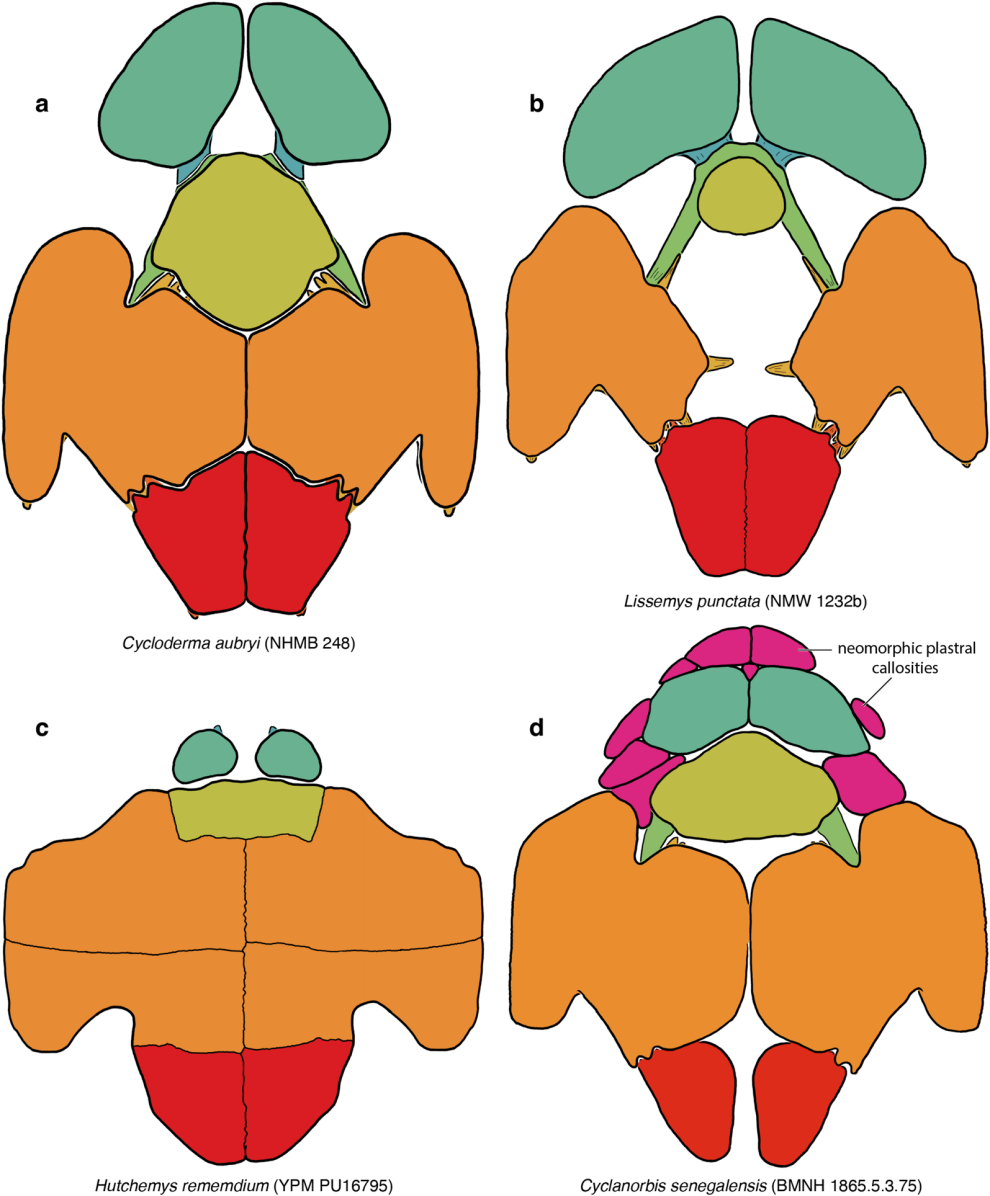
Line drawings of select plastra in ventral view highlighting variation in bone development: **a** *Cycloderma aubryi* (NHMB 248), **b** *Lissemys punctata* (NMW 1232b), **c** *Hutchemys rememdium* (YPM PU16795), **d** *Cyclanorbis senegalensis* (BMNH 1865.5.3.75). Images are not to scale. Colors are used to highlight the anatomical systems labelled in Figs. 1 and 9

Character definition: Neomorphic plastral callosities (as modified by Joyce & Lyson, 2017, p. 12 from Meylan, 1987, p. 9): 0 = absent (Figs. 9, 10, 11a–c); 1 = present (Fig. 11d).

Comments: Plastral callosities are typically associated with one of the seven bones that make up the plastron of pan-trionychids (i.e., the entoplastron and paired epi-, hyo-, hypo-, and xiphiplastra). Numerous supernumerary plastral callosities not associated with these bones, however, are present in *Cyclanorbis senegalensis* (Fig. 11d). As these often disarticulate after death, representatives of *Cyclanorbis* should only be scored for this character, if the plastron is known to be complete. Although all specimens in the sample of *Cyclanorbis senegalensis* available to me possess neomorphic plastral callosities, these structures are known to be absent in hatchlings (McGovern et al., 2021).

### Character 41: Epiplastral callosity

Character definition: Presence and size of sculptured epiplastral callosity (modified from Joyce & Lyson, 2017, p. 8; Meylan, 1987, p. 9): 0 = absent (Figs. 9a, c, 10); 1 = present, but small, callosity only partially covers the epiplastral processes ventrally (Fig. 9b); 2 = present and intermediate in size (Fig. 11c); 3 = present and large (Fig. 11a, b, d).

Comments: Meylan (1987) developed a single character that counts the number of callosities present in the plastron of pan-trionychids. As many pan-trionychid fossils lack a complete plastron that would allow scoring this character, I here follow Joyce and Lyson (2017) by scoring the presence of each plastral callosity separately.

In contrast to Meylan (1987), I restrict this character to epiplastral callosities that have surface texturing, because it is difficult to ascertain the presence of callosities in specimens that lack surface texturing. Minor swellings to the ventral side of the epiplastra are therefore herein disregarded. As I am unaware of forms that exhibit large epiplastral callosities that lack surficial sculpturing, in contrast to the hyo/hypoplastron, this approach appears to be unproblematic.

As a priori knowledge suggests that epiplastral callosities were acquired several times across *Pan-Trionychidae*, I combine the presence and size of epiplastral callosities into a single character that forms a morphocline, as this avoids having a large sample of taxa that are scored inapplicable for a character pertaining to the size of epiplastral callosities in those taxa that exhibit them. Sculptured epiplastral callosities can vary from being a small protrusion that ventrally covers only part of the epiplastron to enormous plates that form much of the anterior plastral lobe (Figs. 9–11). In the available sample, the small condition is mostly found in trionychines (Fig. 9b), the intermediate condition in plastomenids (e.g., Girard et al., 2024; Hutchison, 2009; Joyce et al., 2009; Fig. 11c), and the enlarged condition in cyclanorbines (Fig. 11a, b, d). The large condition typically coincides with a median contact of the epiplastral callosities. Although most taxa show little variation, statistical analyses indicate only a weak relationship with ontogeny (*SCC* = *0.29*; see Additional file 3). This is likely to be an underestimate, as even juveniles of species with large epiplastral callosities have been reported in the literature to exhibit reduced or absent epiplastral callosities (e.g., Deraniyagala, 1939; McGovern et al., 2021). I, therefore, coded taxa by reference to adult specimens, while allowing for polymorphism.

### Character 42: Entoplastral callosity 1

Character definition: Sculptured entoplastral callosity (modified from Meylan, 1987, p. 9 and Joyce & Lyson, 2017, p. 9): 0 = absent (Figs. 9a, 10); 1 = small, callosity only partially covers ventral side of lateral entoplastral processes (Fig. 9b, c); 2 = intermediate, callosity protrudes posteriorly, but less than one half of the median length of the lateral entoplastral processes (Fig. 11b); 3 = large, callosity protrudes posteriorly more than one half of the median length of the lateral entoplastral processes (Fig. 11a, c, d).

Comments: I here follow Joyce and Lyson (2017) by scoring the presence of entoplastral callosities separate from the presence of other plastral callosities, as this allows scoring incomplete fossils. As with the epiplastral callosity, I restrict this character to callosities that have surface texturing, as poorly developed callosities that lack surface texturing are difficult to score unambiguously. All swellings to the ventral side of the entoplastron are therefore scored as absent. As with the previous character, a priori knowledge suggests that epiplastral callosities were acquired several times across *Pan-Trionychidae*. I, therefore, combine the absence versus presence of entoplastral callosities and the relative size of entoplastral callosities into a single character that forms a morphocline, as this avoids creating a character for which most taxa are coded inapplicable. I recognize three derived character states. In species herein defined to have a “small” entoplastral callosity, the callosity only partially covers the median portions of the entoplastron and does not protrude into the triangular space defined by the lateral entoplastral processes (state 1; Fig. 9b, c). In taxa with an “intermediate” entoplastral callosity, the callosity protrudes posteriorly into the triangular space defined by the lateral entoplastral processes, but less than 50% of the depth of this space (state 2; Fig. 10b). In taxa with a “large” callosity, the entoplastron fills more than 50% of the depth of this space (state 3; Fig. 11a, c, d). My sample of polymorphic taxa suggests a weak correlation with ontogeny, in that more adult individuals have proportionally larger entoplastral callosities (*SCC* = *0.41*; see Additional file 3). I, therefore, coded taxa by reference to the most skeletally mature specimen, while allowing for polymorphism if 20% of skeletally mature specimens exhibit a particular character state.

### Character 43: Entoplastral callosity 2

Character definition: Contact of entoplastral callosity with hyo/hypoplastral callosities (new character): 0 = entoplastral callosity absent or, if present, lacks a contact with the hyo/hypoplastral callosity (Figs. 9b, c; 11b, d); 1 = blunt (Fig. 11a); 2 = sutural (Fig. 11c).

Comments: Among pan-trionychids with an entoplastral callosity, variation is apparent to its lateral contacts with the hyo/hypoplastral callosity, in that a contact is either absent (state 0; Figs. 9b, c, 11b, d), blunt (state 1; Fig. 11a), or sutural (state 2; Fig. 11c). In cyclanorbines, a contact is usually absent or blunt, even in taxa with very large entoplastral callosity (Fig. 11a, b, d). The sutured condition, by contrast, is found in extinct species referable to the fossil plastomenid *Hutchemys* (Girard et al., 2024; Hutchison, 2009; Joyce et al., 2009; Fig. 11c), even though they do not have particularly large entoplastra. While the two polymorphic cyclanorbines in my sample show a weak to strong correlation of this characteristic with ontogeny (*SCC* = *0.42* for *Cyclanorbis senegalensis*; *SCC* = *0.89* for *Cycloderma aubryi*; see Additional file 3), none can be found for the only polymorphic trionychine in our sample, *Apalone mutica* (*SCC* = *− 0.08*; see Additional file 3). This is counterintuitive, as all plastral callosities expand during ontogeny, but I suspect that is caused by a lack of juveniles in the sample combined with my inability to distinguish small bodied males from large bodied females due to a lack of sex data. I, therefore, coded all polymorphic taxa by reference to the most skeletally mature specimens. This character forms a morphocline that can be ordered.

### Character 44: Hyo/hypoplastral callosity 1

**Fig. 12:**
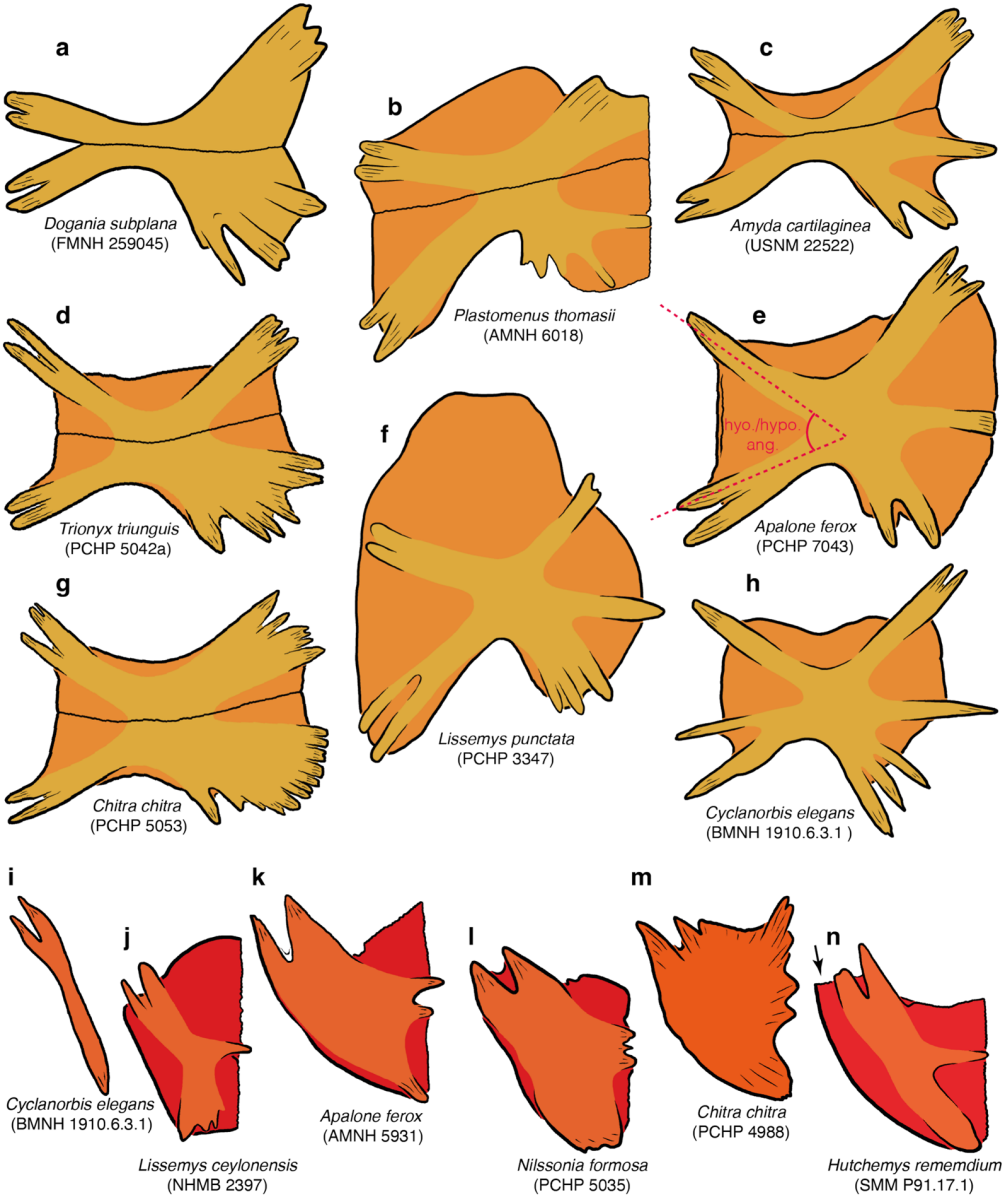
Line drawings of select left hyo/hypoplastra in dorsal view highlighting variation in bone development: **a** *Dogania subplana* (FMNH 259045), **b** *Plastomenus thomasii* (AMNH 6018), **c** *Amyda cartilaginea* (USNM 22522), **d** *Trionyx triunguis* (PCHP 5042), **e** *Apalone ferox* (PCHP 7043), **f** *Lissemys punctata* (PCHP 3347), **g** *Chitra chitra* (PCHP 5053), **h** *Cyclanorbis elegans* (BMNH 1910.6.3.1). Line drawings of select left xiphiplastra in dorsal view highlighting variation in bone development: **i** *Cyclanorbis elegans* (BMNH 1910.6.3.1), **j** *Lissemys ceylonensis* (NHMB 2397), **k** *Apalone ferox* (AMNH 5931), **l** *Nilssonia formosa* (PCHP 5035), **m** *Chitra chitra* (PCHP 4988), **n** *Hutchemys rememdium* (SMM P91.17.1). Images are not to scale. Colors are used to highlight the anatomical systems labelled in Fig. 1. Black arrow highlights lateral extension of hypo-xiphiplastral contact

Character definition: Presence and anterior extent of hyo/hypoplastral callosity (modified from Joyce et al., 2009, p. 75; Joyce & Lyson, 2017, p. 10; Meylan, 1987, p. 9, 23; Vitek et al., 2018, p. 88): 0 = callosity absent; 1 = callosity partially to fully covers hyo/hypoplastral processes and is therefore hidden from dorsal view (Fig. 12a); 2 = callosity protrudes beyond the axillary notch formed by the hyoplastral processes, but does not form an anteriorly protruding lappet (Fig. 12c, d, g, h); 3 = callosity forms a small, anteriorly protruding lappet (Fig. 12b); 4 = callosity forms a large, anteriorly protruding lappet (Fig. 12f).

Comments: Much variation is apparent to the presence and development of the hyo/hypoplastral callosity, of which only little has previously been utilized in phylogenetic analyses. This variation is here captured in five characters that separately address variation to the anterior, lateral, medial, posterior, and posterolateral aspects of this callosity. The resulting characters are partially, but not fully correlated. All characters show a strong ontogenetic component, as juveniles generally have less extensively developed callosities than more skeletally mature individuals.

**Fig. 13:**
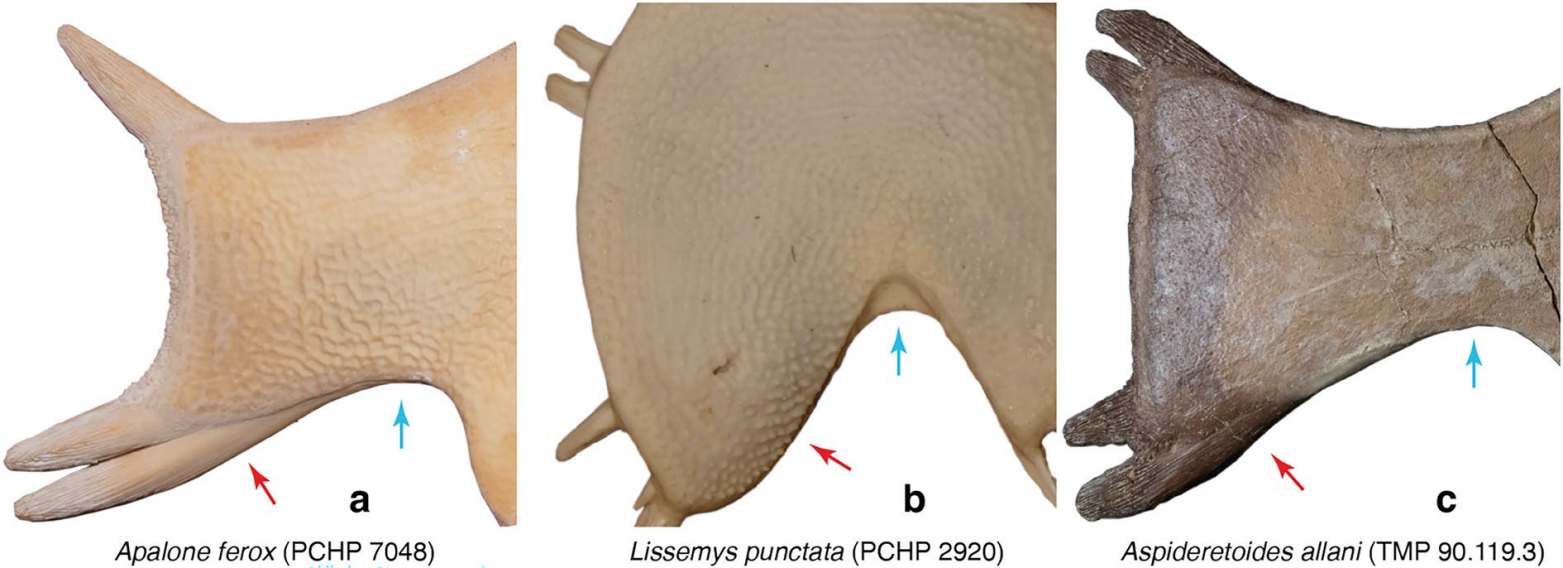
Photographs of select right plastra in ventral view highlighting details in morphology: **a** *Apalone ferox* (PCHP 7048), **b** *Lissemys punctata* (PCHP 2920), **c** *Aspideretoides allani* (TMP 90.119.3). Light blue arrows indicate the inguinal notch, red arrows the posterior margin of the lateral hypoplastral processes. Images are not to scale

The first character from this series scores variation apparent to the presence and anterior extent of the hyo/hypoplastral callosity. In contrast to previous characters that pertain to the presence of callosities, this character does not diagnose the presence of callosities by reference to surface texturing, as some pan-trionychids, such as the late Campanian “*Trionyx*” *allani* (Gardner et al., 1995; Gilmore, 1923; Fig. 13c) possess well-developed callosities (i.e., metaplastic bone that covers the overlying processes in ventral view), but lack surficial sculpturing. The presence of sculpturing is addressed by the following character (see Character 45 below).

Early in ontogeny, the hyo/hypoplastral callosity is absent in trionychids (Sheil, 2003). This condition is retained in the young of some extant trionychids, but universally lost among adults (state 0; not figured). The hyo/hypoplastral callosity at first forms between the axillary and inguinal notches, but does not protrude beyond the margins of the hyo/hypoplastral processes, a condition retained in the adults of some species, such as *Dogania subplana*. The callosity is therefore hidden from view in dorsal view (state 1; Fig. 12a). In most pan-trionychids, the callosity expands anteriorly during ontogeny into the semi-lunate space defined by the hyoplastral processes, but an anteriorly protruding lappet (= “shoulder” or “plastomenid shoulder”) is lacking (state 2). The condition is best scored in visceral view, as the anterior protrusion relative to the hyoplastral processes is only apparent in this view (Fig. 12c, d, g, h). A small, anteriorly convex lappet is apparent in the adults of some extant trionychines and plastomenids (state 3; Fig. 12b), whereas an enlarged lappet is present in some extant cyclanorbines and plastomenids (state 4; Fig. 12f). This anterior lappet may or may not articulate with the entoplastral callosity, but this is addressed in a separate character (see Character 43 above). Statistical analyses yield weak to strong correlations with ontogeny within my sample of polymorphic taxa (*SCC* = *0.39* for all trionychids; *SCC* = *0.61* for all cyclanorbines; *SCC* = *0.91* for *Cycloderma frenatum*; *SCC* = *0.52* for *Amyda* spp.; *SCC* = *0.40* for *Apalone spinifera*; see Additional file 3). Each taxon is therefore coded by reference to the most skeletally mature individuals. This character forms a morphocline that can be ordered.

### Character 45: Hyo/hypoplastral callosity 2

Character definition: Surficial sculpturing of hyo/hypoplastral callosity (modified from Brinkman et al., 2017, p. 67; Vitek, 2012, p. 86): 0 = mostly consisting of raised tubercles (Fig. 13a); 1 = mostly consisting of a netted pattern formed by raised ridges (Fig. 13b); 2 = absent (Fig. 13c).

Comments: The surface sculpturing of pan-trionychid plastra is highly variable. As much variation is apparent at the individual and intraspecific level (also see Character 3 above), I here only recognize three broad categories of plastral sculpturing: a pattern dominated by raised tubercles (Fig. 13a), a pattern dominated by anastomosing raised ridges (Fig. 13b), or the absence of a surface texture (Fig. 13c), which is independent from the lack of a callosity. I liberally scored specimens as polymorphic whenever they display two character states at the same time, e.g., along different portions of the plastron. Although the surface texture of the carapace often matches that of the plastron, many taxa display different surface textures, such as the late Campanian “*Trionyx*” *allani*, which has a netted pattern on the carapace, but lacks texturing on the plastron (Gardner et al., 1995), the late Campanian *Aspideretoides foveatus*, which had a pitted pattern covering the carapace, but a netted pattern on the plastron (Gardner et al., 1995), or the Maastrichtian “*Trionyx*” *beecheri*, which has a netted pattern on the carapace, but possesses raised tubercles on the plastron (Hay, 1904). The use of two separate characters therefore encodes additional information. The three states of this character do not form a morphocline and this character should therefore not be ordered. It is for this reason that I also do not explore ontogenetic trends using statistical analyses. Instead, taxa are coded as polymorphic, if a particular character states is developed in more than 20% of the available sample.

### Character 46: Hyo/hypoplastral callosity 3

Character definition: Fusion of hyo/hypoplastral callosity (modified from Meylan, 1987, 10, 11): 0 = absent (Figs. 9a, b, 10, 11c, 12a–d, g); 1 = present (Figs. 9c, 11a, b, d, 12e, f, h).

Comments: Meylan (1987) noted significant variation in the timing of the fusion of the hyo/hypoplastral callosity, in that it either fuses soon after hatching, later in ontogeny, or never. As the vast majority of extinct pan-trionychids are not known from ontogenetic series and as this matrix is being developed to resolve the relationships of fossil pan-trionychids, I here restrict this character to the simple presence or absence of hyo/hypoplastral fusion (i.e., loss of an apparent suture from the outside; Figs. 9c, 11a, b, d) in a given specimen, while scoring species by reference to the most skeletally mature individual.

### Character 47: Hyo/hypoplastral callosity 4

Character definition: Lateral extent of hyo/hypoplastral callosity (modified from Joyce et al., 2009, p. 76): 0 = callosity absent or small, lateral processes elongate (Figs. 9a); 1 = callosity well-developed, lateral processes are free, but short (Figs. 9b, c, 10); 2 = callosity well-developed and fully covers lateral processes (Fig. 11b, d); 3 = callosity fully covers lateral processes and extends onto dorsal side of shell (Fig. 1d).

Comments: The hyo/hypoplastral callosity is restricted to the junction of the hyo- and hypoplastral processes early in ontogeny of pan-trionychids (e.g., Sheil, 2003; Stoffert, 1889) and the lateral processes are therefore elongate. This condition is retained in the adults of some fossil and recent pan-trionychids (state 0, Fig. 9a), such as *Dogania subplana*. In the majority of pan-trionychids, the callosity laterally expands during ontogeny, but the tips of the lateral processes still remain free, even in adults (state 1; Figs. 9b, c, 10). In adult cyclanorbines and some fossil pan-trionychids, the lateral processes are fully covered by bone in ventral view (state 2; Fig. 11a, d). This morphocline is capped by taxa where the hyo/hypoplastral callosity extends onto the dorsal side of the shell (the peripheral ossification of Joyce et al., 2009). The dorsal aspects of the hyo/hypoplastron are therefore textured with surface sculpturing (state 3; Joyce et al., 2009; Figs. 1d, 10). As this character exhibits a moderate ontogenetic signal (*SCC* = *0.53*; see Additional file 3), which is likely underestimated due to a lack of juveniles, species are coded by reference to the most skeletally mature individuals. The herein developed character states form a morphocline that can be ordered.

### Character 48: Hyo/hypoplastral callosity 5

Character definition: Medial extent of hyo/hypoplastral callosity (modified from Joyce et al., 2009, p. 73): 0 = hyo/hypoplastral callosity absent or small, medial hypoplastral process(es) free and long (Fig. 9a); 1 = hyo/hypoplastral callosity forms a distinct callosity that almost fully or fully covers medial hypoplastral process or processes (Figs. 9b, 10, 11b); 2 = hyo/hypoplastral callosity forms a distinct medial shelf that fully covers medial process, a median butt contact of the callosities may or may not be present (Fig. 11a, d); 3 = hyo/hypoplastral callosities broadly sutured with one another along the midline (Figs. 9c, 11c).

Comments: The hyo/hypoplastral callosity is absent or small early in ontogeny of pan-trionychids (e.g., Sheil, 2003; Stoffert, 1889) and the medial hypoplastral process or processes are therefore free and elongate (Fig. 9a). In the available sample of extant trionychids, this condition is not retained in the adult of any taxon, but this state is retained nonetheless, to allow scoring juveniles and for possible use in adult fossil taxa. In the adults of many pan-trionychids, the hyo/hypoplastral callosity is well developed and forms a medial protrusion that almost fully or fully covers the medial process (Figs. 9b, 10, 11b). In others, the hyo/hypoplastral callosity forms a distinct medial shelf that fully covers medial process (Fig. 11a, d). A medial contact of the hyo/hypoplastral callosity may or may not be present. Finally, the hyo/hypoplastral callosities of some pan-trionychids, mostly fossil forms such as *Hutchemys rememdium* (Girard et al., 2024; Joyce et al., 2009), have an extensive sutural contact with one another along the midline (state 3; Figs. 9c, 11c). As a full gradation is apparent between the character states of this morphocline, specimens were liberally scored intermediate. This character shows polymorphic variation in almost every single sampled species. Statistical analysis highlights a moderate correlation with ontogeny (*SCC* = *0.52* for all taxa; *SCC* = *0.58* for *Trionychinae*; see Additional file 3), in that more skeletally mature individuals have medially better developed callosities. Species are, therefore, coded by reference to the most skeletally mature individuals.

### Character 49: Hyo/Hypoplastral callosity 6

Character definition: Contribution of hyo/hypoplastral callosity to inguinal notch (new character): 0 = inguinal notch formed by hypoplastral process (Fig. 13b, light blue arrow); 1 = inguinal notch formed by hyo/hypoplastral callosity (Fig. 13a, c, light blue arrows).

Comments: In ventral view, the deepest aspect of the inguinal notch is either formed by the hypoplastral process (state 0; Fig. 13b, light blue arrow) or more superficially by the hyo/hypoplastral callosity (state 1; Fig. 1a, c, green arrows). I am unaware of any taxon where the callosity protrudes posteriorly beyond the deeper part of the inguinal notch. Although this character at first sight seems to correlate with the overall development of the hyo/hypoplastral callosity, an uncovered inguinal notch is both found in taxa with poorly developed callosities, such as *Dogania subplana*, and extremely well-developed callosities, such as *Lissemys punctata*. This character shows a weak correlation with ontogeny (*SCC* = *0.32* for all taxa; *SCC* = *0.44* for *Trionychinae*; see Additional file 3) and species are therefore coded by reference to the most skeletally mature individual available.

### Character 50: Hyo/hypoplastral callosity 7

Character definition: Posterolateral extent of hyo/hypoplastral callosity (modified from Joyce & Lyson, 2017, p. 93): 0 = posterior aspects of the lateral hypoplastral processes not covered by the hyo/hypoplastral callosity (Fig. 13a, c, red arrows); 1 = posterior aspects covered by the hyo/hypoplastral callosity (Fig. 13c, red arrow).

Comments: In the majority of pan-trionychids, the hyo/hypoplastral callosity is relatively flat and does not cover the posterior aspects of the lateral hypoplastral processes (state 0; Fig. 13a, c, red arrows). In an eclectic group of pan-trionychids, in particular cyclanorbines and plastomenids, the hyo/hypoplastral callosity expands posterolaterally to posteriorly cover the process (state 1; Fig. 13c, red arrow). The callosity thereby gains a third dimension as the callosity “rolls” over the posterior edge of the process. As a moderate ontogenetic component is apparent, with juveniles having less developed hyo/hypoplastral callosities (*SCC* = *0.53* for all taxa; see Additional file 3), species are coded by reference to the most skeletally mature individuals. A full gradation is apparent between the two characters states and intermediates are therefore liberally coded as polymorphic.

### Character 51: Xiphiplastral callosity I

Character definition: Presence and extent of xiphiplastral callosity (modified from Joyce & Lyson, 2017, p. 11; Meylan, 1987, p. 9; Vitek, 2012, p. 87): 0 = callosity absent (Figs. 9a, 12i); 1 = callosity present that only partially covers the overlying xiphiplastral processes in ventral view (Figs. 10c, 12m); 2 = callosity present that fully covers the xiphiplastral processes in ventral view, but does not laterally protrude significantly beyond the lateral margins of the xiphiplastral processes (Fig. 12k, l); 3 = callosity present that fully covers the xiphiplastral processes and clearly protrudes laterally beyond the margins of the xiphiplastral processes (Fig. 12j, n).

Comments: Much variation is apparent in the development of the xiphiplastral callosity in pan-trionychid turtles. As with the epiplastral and entoplastral callosities, the xiphiplastral callosity is here defined by the presence of surface texturing. A swelling on the ventral side of the xiphiplastral processes is therefore herein not recognized as a callosity. Early in ontogeny, pan-trionychids lack a callosity on the xiphiplastron (Sheil, 2003). This condition is retained in the adult of some species, such as *Rafetus euphraticus* (state 0; Figs. 9a, 12i). The callosity initially forms within the confines of the xiphiplastral processes (state 1; Figs. 10c, 12m), but may expand to fully cover the processes in ventral view (state 2; Fig. 12k, l). Either condition is once again apparent in the adults of various species. In some forms, the callosity expands posterolaterally beyond the margins of the xiphiplastral processes (state 3; Fig. 12j, n). The latter two character states can only be distinguished rigorously in dorsal view, as the margin of the xiphiplastral processes relative to the xiphiplastral callosity needs to be clear. This character is strongly correlated with other characters pertaining to the development of plastral callosities in that individuals with better-developed xiphiplastral callosities tend to have better developed plastral callosities as well, but the correlation is not strict (e.g., xiphiplastral callosities are absent in *Cyclanorbis elegans* even though it has well developed hyo/hypoplastral callosities) and I therefore retain it as separate. A weak to moderate ontogenetic component is apparent for this character (*SCC* = *0.35* for all taxa; *SCC* = *0.57* for *Trionychinae*; see Additional file 3) so species are coded by reference to the most skeletally mature individual. This character forms a morphocline that can be ordered.

### Character 52: Xiphiplastral callosity II

Character definition: Anterior contacts of xiphiplastral callosity with hyo/hypoplastral callosity (new character): 0 = callosities absent or, if present, lack a contact with one another (Figs. 9a, 10); 1 = partially to fully abutting (Figs. 9b, 11a, b, d); 2 = sutured, but with without lateral extension (Fig. 9c); 3 = sutured and with lateral extension along the suture (Figs. 11c; 12n).

Comments: The xiphiplastral callosity can have one of several relationships with the adjacent hyo/hypoplastral callosities. In the majority of extant pan-trionychids, these two callosities are either spatially separated from one another (state 0; Figs. 9a, 10), or they have a blunt butt contact (state 1; Figs. 9b, 11a, b, d). This contact typically follows the outline of the posterior hypoplastral and anterior xiphiplastral processes and therefore looks to be interdigitated, but nevertheless lacks signs of true suturing. In a number of extinct pan-trionychids, such as *Hutchemys rememdium* (Girard et al., 2024; Hutchison, 2009; Joyce et al., 2009), the hyo/hypoplastral and the xiphiplastral callosities form a finely interdigitated suture at their contacts lateral to the deeply interdigitated contact of the hypo- and xiphiplastra (state 2). The suture either ends at the lateral margins of the hypoplastral and xiphiplastral processes or protrudes beyond these margins to form a lateral extension (state 3; Figs. 11c; 12n, black arrow). Specimens that lack either callosity are scored with the 0 state so that phylogenetic software cannot interpolate taxa scored as inapplicable (e.g., lacking a callosity) with non-applicable scorings (e.g., having a large contact). As a morphocline is apparent, specimens are liberally scored intermediate. This character shows an ontogenetic component (*SCC* = *0.46* for all taxa; see Additional file 3). Species are therefore coded by reference to the most skeletally mature individual.

### Character 53: Xiphiplastral callosity III

Character definition: Median contacts of xiphiplastral callosity (modified from Joyce & Lyson, 2017, p. 14; Meylan, 1987, p. 12): 0 = xiphiplastral callosities absent or median contact absent when present (Figs. 9a, 10, 11d); 1 = partially to fully abutting (Fig. 8b); 2 = sutured or fused (Figs. 9c, 11a, b, c).

Comments: If present, the median contact of the xiphiplastral callosities can range from being absent (state 0; Figs. 9a, 10, 11d), to abutting (state 1; Fig. 8b), to sutured, or fused (state 2; Figs. 9c, 11a, b, c). As some taxa with fused xiphiplastra look to be abutting in ventral view (e.g., *Cyclanorbis aubryi*; Fig. 11a) and as fusion cannot be established rigorously in fossil material, the distinct fused character state developed by Meylan (1987) is not utilized herein. Specimens that lack xiphiplastral callosity are scored with the primitive state to avoid phylogenetic software from interpolating taxa scored as inapplicable with non-applicable scorings. This character shows a moderate ontogenetic component, with larger taxa having largest xiphiplastral callosities with more intimate midline contacts (*SCC* = *0.51* for all taxa; see Additional file 3). Species are therefore coded by reference to the most skeletally mature individual. This character forms a morphocline that can be ordered.

### Character 54: Epiplastral processes I

**Fig. 14:**
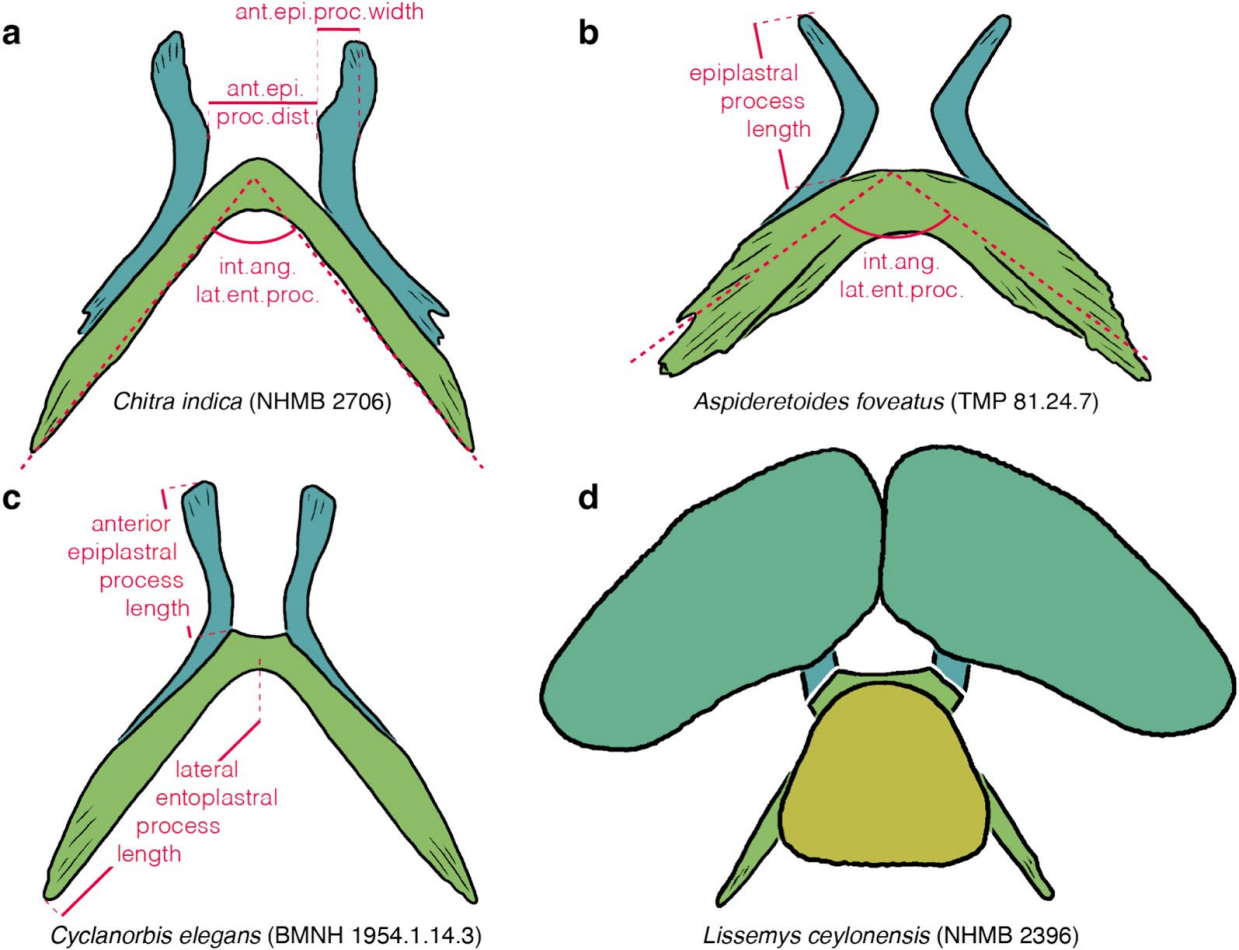
Line drawings of select epi- and entoplastra in ventral view highlighting variation in bone development: **a** *Chitra indica* (NHMB 2706), **b** *Aspideretoides foveatus* (TMP 81.24.7), **c** *Cyclanorbis elegans* (BMNH 1954.1.14.3), **d** *Lissemys ceylonensis* (NHMB 2396). Images are not to scale. Colors are used to highlight the anatomical systems labelled in Fig. 1. int.ang.lat.ent.proc., internal angle of the lateral entoplastral processes; ant.epi.proc.dist., anterior epiplastral process distance; ant.epi.proc.width, anterior epiplastral process width

Character definition: Length of anterior epiplastral processes (Fig. 14c) relative to length of the lateral entoplastral processes (modified from Meylan, 1987, p. 20; Fig. 14c): 0 = less than 50%; 1 = 50–80%; 2 ≥ 80%.

Comments: The length of the anterior epiplastral processes varies greatly among pan-trionychids. A full gradation is apparent so Meylan (1987) measured the length of the anterior plastral process relative to the width of the hyoplastron. As the vast majority of measurements utilized herein are taken from photographs, I here measure the length of the epiplastron relative to the entoplastron, as both structures are typically photographed together. The size of the entoplastron and hyoplastron do not scale isometrically among pan-trionychids, so the relative measurements obtained herein do not overlap with those of Meylan (1987), although the trends remain the same. It should be noted that the anterior epiplastral processes supports a movable flap, so that measurements are easily distorted in dried specimens, as the flap with the anterior epiplastral processes may fold away from the plane of the entoplastron. Specimens that score within 5 percent points of the cut off between character states are scored intermediate (i.e., polymorphic). Although no clear ontogenetic trends are apparent in the available data (*SCC* = *0.12* for all taxa; see Additional file 4), much interspecific variation is apparent in well-sampled species. This variation is captured through the liberal use of polymorphic scoring. A number of taxa, in particular cyclanorbines, are not coded for this character because the epiplastral process cannot be distinguished from the underlying callosity in the available material. Straight measurements are obtained for rare taxa with recurved anterior epiplastral processes (Fig. 14b). This character forms a morphocline that can be ordered.

### Character 55: Epiplastral processes II

Character definition: Posterior epiplastral processes (modified from Meylan, 1987, p. 19): 0 = absent (Figs. 11a, b, 14d); 1 = present (Figs. 9, 10, 14a, b, c).

Comments: Meylan (1987) developed a character that utilizes shape variation to the epiplastral processes. In the majority of pan-trionychids, the anterior epiplastral processes support the flexible anterior flap of the anterior plastral lobe, while the posterior epiplastral processes articulate broadly with the entoplastron. These two processes typically stand in a wide angle to one another ("J-shape" of Meylan, 1987, state 1; Figs. 9, 10, 14a, b, c). In most cyclanorbines, by contrast, the posterior epiplastral processes are absent (I"-shape" of Meylan, 1987, state 0; Figs. 11a, b, 14d) and the remaining anterior plastral processes therefore articulate directly with the entoplastron along a short contact. This character neither shows ontogenetic or intraspecific variation. Single individuals are therefore representative for the entire species.

### Character 56: Epiplastral processes III

*Character definition:* Distance between anterior epiplastral processes (new character): 0 = widely spaced, anterior epiplastral processes spaced > 1.5 times the width of an anterior epiplastral process; 1 = anterior epiplastral processes spaced 0.5–1.5 times the width of an anterior epiplastral process; 2 = close, anterior epiplastral processes spaced < 1.5 times the width of an anterior epiplastral process or in medial contact with one another (Fig. 14a).

Comments: Following Siebenrock (1909), I recognize substantial variation to the spacing between the anterior epiplastral processes. In all pan-trionychids, the anterior epiplastral processes support the soft anterior plastral lobe, but the anterior processes are either widely (state 0), intermediately (1), or tightly spaced or contacting (state 2). To allow scoring this character objectively, the space is measured as the closest distance between the anterior epiplastral processes relative to the width of the processes themselves (Fig. 14a). There is no tight correlation between the shape of the entoplastron (see Character 57 below) and the spacing of the epiplastra in that various taxa with an anteriorly pointed entoplastron can have widely (e.g., *Trionyx triunguis*), intermediately (e.g., *Rafetus euphraticus*), or tightly spaced epiplastra (e.g., *Amyda cartilaginea*). I, therefore, only score this character by reference to articulated specimens, but suspect that disarticulated specimens can be scored as well, if the exact nature of the epiplastral/entoplastral contact is clear. As a morphocline is apparent in the available sample, polymorphism is used liberally to score for intermediates. This character lacks an ontogenetic component (*SCC* = *0.04* for all polymorphic taxa; see Additional file 4). Taxa are only coded polymorphic if more than 20% of individuals show a particular character state.

### Character 57: Entoplastral processes I

Character definition: Shape of lateral entoplastral processes (modified from Joyce et al., 2009, p. 74): 0 = lateral entoplastral processes are straight and anteriorly merge at a point (Fig. 14a); 1 = lateral entoplastral processes are straight, but form a narrow transverse shelf anteriorly (Fig. 14c); 2 = lateral entoplastral processes combined form a crescent-shaped element (Fig. 14b); 3 = lateral entoplastral processes are straight, but form a broad transverse shelf anteriorly (Fig. 14d).

Comments: The overall shape of the entoplastron varies greatly among pan-trionychids. I here recognize four shape categories: the lateral entoplastral processes are either straight and anteriorly converge upon a point (state 0; Fig. 14a), or they are straight, but form a narrow (state 1; Fig. 14c) or broad (state 3; Fig. 14d) anterior shelf, or they combined form a rounded, crescent shaped element (state 2; Fig. 14b) best seen in extinct plastomenids (Gardner et al., 1995; Girard et al., 2024; Hay, 1908). This character is not correlated with the distance between the anterior epiplastral processes, as taxa with widely and closed spaced epiplastra can have the same entoplastral shape (see Character 56 above). A full gradation is apparent between all four character states, so specimens are scored polymorphic when necessary. The nature of the character, not being a morphocline, prohibits the use of statistics to explore ontogenetic trends, but an informal assessment suggests that ontogeny does not have an effect on this character, as the shape of juvenile plastra resembles those of adults. Taxa are coded polymorphic if a particular character state is apparent in more than 20% of the population.

### Character 58: Entoplastral processes II

Character definition: Interior angle between the lateral entoplastral processes (new character): 0 = notably acute, less than 80°; 1 = about right, between 80° and 100°; 2 = notably obtuse, more than 100° (Fig. 14a).

Comments: The angle formed by the two lateral entoplastral processes differs substantially among pan-trionychids ranging from notably acute (state 0) to notably blunt (state 2). As all taxa show much intraspecific variation to this angle and as it is difficult to measure this angle objectively, I here only establish three character states that group the most extreme configurations. All measurements were taken from pictures following the medial margins of the entoplastron or the striations that are typically visible along the medial margins of the entoplastron in dorsal and ventral view (Fig. 14a). Specimens are scored polymorphic if their measurement approaches a threshold within 3 degrees. As the angle between the two lateral entoplastral processes is greatly distorted in oblique views, I only utilized specimens with isolated entoplastra or flat, articulated plastra photographed in straight ventral view. A decisive ontogenetic signal is not apparent (*SCC* = *− 0.15*; see Additional file 4). Taxa were only coded polymorphic if at least 20% of individuals exhibit a particular character state. Intermediate individuals were hereby grouped with the dominant character state. This character forms a morphocline that can be ordered.

### Character 59: Entoplastral processes III

Character definition: Thickness of lateral entoplastral processes (new character): 0 = notably thin, lacking horizontal expansions (Fig. 14a, d); 1 = intermediate, with minor horizontal expansions (Fig. 14c); 2 = notably wide, with well-developed horizontal expansions (Fig. 14b).

Comments: The lateral processes of the entoplastron shows considerable variation across *Pan-Trionychidae* in regard to their dimensions ranging from elongate and rib-like (state 0), to slightly expanded (state 1), and strongly expanded (state 2). In all cases, the expansions are plate like and oriented along the horizontal plane. A full gradation is apparent, but I was not able to quantify the available variation in a meaningful way. I, therefore, generously score specimens with intermediate morphotypes polymorphic. Statistical analysis fails to discover a correlation of this character with ontogeny (*SCC* = − *0.03*; see Additional file 4). I, therefore, coded taxa polymorphic if a character state is found in at least 20% of individuals. Intermediate individuals were hereby grouped with the dominant character state. This character forms a morphocline that can be ordered.

### Character 60: Hyo- and hypoplastral processes I

Character definition: Number of lateral hyoplastral processes (Joyce & Lyson, 2011, p. 77): 1 = one (Figs. 9b, c, 12e, h); 2 = two (Figs. 9a, 10, 12a–d, g); 3 = three.

Comments: The number of lateral hyoplastral processes varies in the available sample from one to three. The latter condition is never developed consistently across any species, but is retained to allow scoring individuals. To ease scoring, the character state number corresponds to the number of processes present. Asymmetric specimens are scored polymorphic. Taxa are coded as polymorphic if a particular character state occurred in at least 20% of the available sample. Although most taxa show a consistent pattern, much variation is apparent across the tree. Variation is particularly high among cyclanorbines, as representatives of this group typically form a single hyoplastral process that may or not be subdivided distally into subparts. This character forms a morphocline and can therefore be ordered.

### Character 61: Hyo- and hypoplastral processes II

Character definition: Number of lateral hypoplastral processes (new character): 1 = one; 2 = two (Figs. 9, 10a, b, c [right], 12a–h); 3 = three (Fig. 10c [left]).

Comments: This character pertains to the lateral processes of the hypoplastron. Similar to the previous character, character state numbers correspond to the number of processes present and asymmetric specimens (e.g., Fig. 10c) are scored as polymorphic. Although variation is apparent across the tree, the vast majority of taxa show variation far below a 20% threshold. The only exception are *Chitra* spp., which show three processes in about two thirds of specimens. Although I would normally score a taxon with this frequency as polymorphic using the 20% threshold used otherwise, I coded all *Chitra* spp. for the derived character state, as this easily scorable character would otherwise be rendered uninformative. The condition with a single lateral hypoplastral process is only known among some Paleogene representatives of the extinct *Axestemys* lineage (Pérez-Garcia & Smith, 2021; Vitek, 2012). This character forms a morphocline that can be ordered.

### Character 62: Hyo- and hypoplastral processes III

Character definition: Angle between the adjacent lateral processes of the hyo- and hypoplastron (new character): 0 ≤ 45°; 1 = 45–70°; 2 ≥ 70° (Fig. 12e). Specimens approaching a given threshold within 3 degrees are scored polymorphic.

Comments: Variation is apparent to the angle formed between the most posterior lateral hyoplastral process and the most anterior lateral hypoplastral process. The angle was herein measured from photographs of skeletal specimens. As this angle changes based on perspective, measurements were only obtained from pictures of isolated hyo/hypoplastra or flat plastra taken in straight vertical views. The prevailing striations of both elements hereby served as guides for the sides of the angle (Fig. 12e). The available morphocline was divided into three character states. Individuals that approach thresholds within 3 degrees were scored polymorphic. Although the data globally does not show a tight relationship between the angle of the lateral processes and ontogeny (*SCC* = − *0.04*; see Additional file 4), individual taxa somewhat surprisingly show a weak or moderate effect (*CC* = *0.28* for *Lissemys scutata*; *CC* = *0.54* for *Amyda ornata*; see Additional file 4) in that more skeletally mature individuals have a larger angle. I, therefore, resolve polymorphic scoring within a species by reference to more skeletally mature individuals.

### Character 63: Hyo- and hypoplastral processes IV

Character definition: Cross section of the lateral hyo/hypoplastral processes (new character): 0 = rounded; 1 = flattened.

Comments: The cross section of the lateral hyo- and hypoplastral process ranges from rounded (state 0) to flattened (state 1). Among extant trionychids, the rounded condition is dominant. Intermediately flattened processes are common among cyclanorbines. Large, fully flattened lateral processes are most commonly developed among Paleogene representative of the now extinct lineage *Axestemys* (Pérez-Garcia & Smith, 2021; Vitek, 2012). This character shows no relationship with ontogeny (*SCC* = *0.11*; see Additional file 4). Taxa are therefore coded by reference to the dominant condition apparent within the species. Intermediate morphologies are hereby attributed to the dominant character state. As the derived character state is only known among extinct pan-trionychids, this character is uninformative among extant trionychids.

### Character 64: Hyo- and hypoplastral processes V

Character definition: Spacing of the lateral hypoplastral processes (new character): 0 = closely packed (Fig. 12a–f); 1 = strongly flared (Fig. 12h).

Comments: The posterior hypoplastra process of the majority of pan-trionychids point in the same direction and are therefore closely packed (state 0). The posterior hypoplastral process of most cyclanorbines, by contrast, point in different directions and are therefore strongly flared (state 1). An intermediate condition, herein scored polymorphic, is apparent in many individuals of *Apalone* spp. This character shows no correlation with ontogeny (*SCC* = *0.04*; see Additional file 4). All polymorphism apparent in a population is therefore retained if at least 20% of individuals exhibit a particular character state. Specimens scored intermediate are hereby attributed to the more commonly observed state.

### Character 65: Hyo- and hypoplastral processes VI

Character definition: Number of medial hyoplastral processes (modified from Brinkman et al., 2017, p. 85, 86): 0 = expansive medial hyoplastral comb present consisting of ≥ 6 processes (Figs. 10c, 12g); 1 = an intermediate medial hyoplastral comb present consisting of 2–5 processes (Fig. 9b, 10a, b, 12a, c, d, e); 2 = a finger-like medial hyoplastral process present consisting of 1–3 sub-processes (Fig. 9a, 11b, 12f, h).

Comments: A large amount of variation is apparent in pan-trionychids in regard to the development of the medial hyoplastral processes (Brinkman et al., 2017). I, here, note a gradation from an expansive medial comb consisting of six or more tightly packed finger-like processes (state 0; Figs. 10c, 12g), to a narrow comb consisting of 2–5 tightly packed processes (state 1; Figs. 9b, 10a, b, 12a, c, d, e), to a finger-like process that may be subdivided into 1–3 tightly packed subprocesses (state 2; Figs. 9a, 11b, 12f, h). The three morphoclinic character states are not discrete and specimens with intermediate morphologies were liberally scored as polymorphic. A clear majority of taxa shows no polymorphism, but those that do show no relationship with ontogeny (*SCC* = *0.13*; see Additional file 4). Species are therefore only scored polymorphic if a particular character state is developed in at least 20% of individuals. Specimens scored intermediate are attributed to the more commonly observed state.

### Character 66: Hyo- and hypoplastral processes VII

Character definition: Number of posteromedial hypoplastral processes (modified from Brinkman et al., 2017, p. 87): 0 = expansive posteromedial hypoplastral comb present consisting of numerous processes, but no gaps (Figs. 10c, 12g); 1 = uneven posteromedial hypoplastral comb present consisting of numerous processes and occasional gaps (Fig. 12d); 2 = a single medial process developed in clear separation from a small number of posterior processes (Figs. 9a, b, 10a, b, 11b, 12a, c, e, f, h).

Comments: As already noted by Brinkman et al. (2017), the posteromedial processes of the hypoplastra show significant variation. I here note three morphotypes. In the first, an expansive posteromedial comb is developed consisting of tightly packed, radially arranged and equally sized processes (state 0; Figs. 10c, 12g). In the second, a comb is still developed, but the processes are unevenly packed, vary in size, and minor gaps may be developed (state 1; Fig. 12d). In the third, a single medial process is developed that is well separated from a small number of posterior processes (state 2; Figs. 9a, b, 10a, b, 11b, 12a, c, e, f, h). Specimens with intermediate morphologies are liberally scored as polymorphic. This character shows no variation within most extant trionychid species, but those that exhibit polymorphism show no relationship with ontogeny (*SCC* = *0.13*; see Additional file 4). As a result, species are only coded polymorphic if a character state is developed in more than 20% of individuals. Specimens with intermediate scoring are hereby attributed to the more commonly observed state. This character forms a morphocline that can be ordered.

### Character 67: Hyo- and hypoplastral processes VIII

Character definition: Posterior hypoplastral processes lateral to xiphiplastron (modified from Meylan, 1987, p. 13): 0 = absent (Figs. 9, 10); 1 = present (Fig. 11a, b, d).

Comments: Meylan (1987) noted that the hypoplastron of all cyclanorbines produces a third, posterior process (Fig. 12f, h) that laterally embraces the anterior processes of the xiphiplastron. The process is absent in all other pan-trionychids, including plastomenids. My sample lacks polymorphic individuals or polymorphic taxa.

### Character 68: Xiphiplastra processes I

Character definition: Number of medial xiphiplastral processes (new character): 0 = none (Fig. 12i); 1 = one or two (Fig. 12j, k, n); 2 = three (Fig. 12l); 3 = four or more (Fig. 12m).

Comments: The number of medial xiphiplastral processes ranges from none in representatives of *Cyclanorbis* spp. (Fig. 12i) to a broad comb that spans the full width of the element in representatives of *Chitra* spp. (Fig. 12m). As much variation is apparent within individuals and within species, I here define four character states that capture the most common arrangements, but liberally score taxa as polymorphic. This character is difficult to score in some specimens with well-developed xiphiplastra callosities, as contrast may be poor between the callosities and the medial processes. Such specimens are scored unknown. This character does not show an ontogenetic component (*SCC* = *0.19*; see Additional file 4) but forms a morphocline that can be ordered. Taxa are coded polymorphic if more than 20% of individuals exhibit a particular character state. Intermediate scorings are hereby attributed to the more common of the adjacent character states.

### Character 69: Xiphiplastra processes II

Character definition: Orientation of posterior xiphiplastral processes (new character): 0 = posteromedially oriented towards each other (Figs. 9a, 10, 12k, l, m, n); 1 = posteriorly arranged parallel to each other (Figs. 11a, 12i, j).

Comments: In the vast majority of pan-trionychids, the posterior processes of both xiphiplastra form compact combs that are oriented posteromedially and therefore converge upon a point at the midline (state 0; Figs. 9a, 10, 12k, l, m, n). This differs clearly from the condition seen in cyclanorbines, where the posterior processes form broad combs that are oriented posteriorly, somewhat parallel to one another and therefore do not converge towards the midline (Figs. 11a, 12i, j). No intraspecific variation is apparent for this character.

## Results and discussion

### Phylogenetic nomenclature

*Amydini* Kordikova, 1994, converted clade name.

*Registration number*: 1093.

*Definition*: The largest crown clade containing *Amyda* (orig. *Testudo*) *cartilaginea* (Boddaert, 1770), but not the apalonine *Apalone* (orig. *Trionyx*) *spinifera* (LeSueur, 1827) or the chitrine *Chitra* (orig. *Trionyx*) *indica* (Gray, 1830).

*Reference phylogeny*: Thomson et al., (2021, Fig. 1).

*Composition*: *Amydini* is currently hypothesized to consist of 15 extant species distributed across much of Asia (TTWG, 2021). The fossil record is mostly unresolved, but likely includes select taxa from the Cenozoic of Asia (Georgalis & Joyce, 2017; Massonne et al., 2023).

*Not established phylogenetic definitions*: Amydona Engstrom et al. (2004).

*Diagnostic Apomorphies*: see Osteological Characterization of Extant Clades and Species below.

*Apalonini* Khosatzky, 1999, converted clade name.

*Registration Number*: 1094.

*Definition*: The largest crown clade containing *Apalone* (orig. *Trionyx*) *spinifera* (LeSueur, 1827), but not the amydine *Amyda* (orig. *Testudo*) *cartilaginea* (Boddaert, 1770) or the chitrine *Chitra* (orig. *Trionyx*) *indica* (Gray, 1830).

*Reference Phylogeny*: Thomson et al., (2021, Fig. 1).

*Composition*: *Apalonini* is currently hypothesized to consist of five extant species with a patchy distribution across the northern hemisphere (TTWG, 2021). At present, only a small number of fossils from the Neogene can be attributed to this clade with confidence (Georgalis & Joyce, 2017; Vitek & Joyce, 2015).

*Not established phylogenetic definitions*: Apalonina Engstrom et al. (2004).

*Diagnostic Apomorphies*: see Osteological Characterization of Extant Clades and Species below.

*Chitrini* Meylan, 1987, converted clade name.

*Registration Number*: 1095.

*Definition*: The largest crown clade containing *Chitra* (orig. *Trionyx*) *indica* (Gray, 1830), but not the amydine *Amyda* (orig. *Testudo*) *cartilaginea* (Boddaert, 1770) or the apalonine *Apalone* (orig. *Trionyx*) *spinifera* (LeSueur, 1827).

*Reference Phylogeny*: Thomson et al., (2021, Fig. 1).

*Composition*: *Chitrini* is currently hypothesized to consist of seven extant species distributed across much of Africa and Eurasia (TTWG, 2021). While a number of fossils from the Neogene of these land masses unambiguously represent this clade, countless fossils published under the name “*Trionyx*” must be viewed with more caution (Georgalis & Joyce, 2017; Vitek & Joyce, 2015).

*Not established phylogenetic definitions*: Gigantaestuarochelys Engstrom et al. (2004); Chitrainae Jasinski et al. (2022),

*Diagnostic Apomorphies*: see Osteological Characterization of Extant Clades and Species below.

*Comments*: As the phylogeny of trionychids had not been resolved until quite recently, there is little tradition on how to name various subgroups, beyond the dichotomy of crown *Trionychidae* into crown *Cyclanorbinae* and crown *Trionychinae* (Engstrom et al., 2004; Hummel, 1929; Joyce et al., 2004, 2021; Meylan, 1987). Plenty of names have nonetheless been proposed over the course of the decades, such as Chitradae Gray (1870), Chitraina Gray (1873), Chitrinae Chkhikvadze (1970), Chitrini Meylan (1987), Chitrina Meylan (1987), or Chitrainae Jasinski et al. (2022) for more or less inclusive groups that include *Chitra indica*. Meylan (1987) suggested subdividing *Trionychinae* into four tribes: Chitrini, Aspideretini, Trionychini, and Pelodiscini. Engstrom et al. (2004) proposed dividing *Trionychinae* into three phylogenetically defined clades: Gigantaestuarochelys, Apalonina, and Amydona. Finally, Jasinski et al. (2022) proposed dividing the same clade into the phylogenetically defined clades Chitrainae and Trionychinae (sensu strictu). Although the topology has remained relatively stable in the last two decades, the nomenclatural systems of Engstrom et al. (2004) and Jasinski et al. (2022) are used little, perhaps because the newly created names are uneven in nature and somewhat difficult to pronounce or do not follow nomenclatural tradition. I, therefore, herein formally define and utilize a phylogenetic nomenclature that follows the system laid out by Meylan (1987) in the formation of names, but Engstrom et al. (2004) in the selection of clades to be named and their specifiers, in particular *Amydini* [Kordikova, 1994], *Apalonini* [Khosatzky, 1999], and *Chitrini* [Meylan, 1987]. Representatives of these three clades are Anglicized herein as amydines, apalonines, and chitrines.

### Preneural

The shell of turtles is now universally agreed to be a composite structure formed, among others, through periosteal outgrowths of the dorsal ribs and the neural arches of the dorsal vertebrae into the superficial dermis, which result in the formation of costals and neurals, respectively (Cherepanov, 1997; Kälin, 1945; Suzuki, 1963). The correspondence between dorsal ribs and costals is easy to observe in visceral view in that the first dorsal ribs are reduced, the second through nineth dorsal ribs form costals, and the tenth dorsal rib is reduced again. As the costals are traditionally labelled "I" through "VIII" from front to back, dorsal rib II forms costal I, dorsal rib III costal II, etc. Kordikova (2000) suggested relabeling costals I–VIII as costals II–IX to more clearly highlight their homology with the vertebral column, but this proposal has mostly been ignored.

The correspondence of the dorsal vertebrae with the above-lying neurals is less apparent for two primary reasons. First, the low neural arches are obscured from sight, making it difficult to assess which neural connects with which neural arch. Second, turtles are unique among vertebrates in that the vertebral centra are shifted posteriorly by a half segment relative to the ribs and neural arches (Goette, 1899). As a result, the vertebral centra are posteriorly offset from their neural arches and neurals, thereby further obscuring correspondence of elements.

Meylan (1987) noted that the preneural of pan-trionychids, whenever present, is associated with the first thoracic vertebra and concluded that it should be identified as the first neural instead. While I agree with Meylan's (1987) observation, I note that the first neural of other turtles (e.g., *Chelydra serpentina*, *Podocnemis expansa*, *Trachemys scripta*) is not connected to the first dorsal arch, but rather with the second. It therefore appears that Meylan (1987) accidentally equated the location of the neural body with the location of the neural arch. The preneural of pan-trionychids is therefore an *additional* element relative to the shell of other turtles and the use of a separate name, as introduced by Hay (1904), appears justified.

Cherepanov (1995) and Kordikova (2002) arrived at the same conclusion as I did, but using different rationales. Cherepanov (1995) believed that the preneural does not arise from the periosteum of the first dorsal arch, but rather only sutures to it later during ontogeny. Although this observation seems to further supports my conclusion, I note that Cherepanov (1995) did not cite any data or study that would support his initial assertion. To the contrary, Kordikova (2000) stated that the preneural does arrive from the periosteum of the first dorsal arch, but I note, again, that no data or study is cited that would support her claim. Her decision to relabel the preneural as the first neural, however, was not intended to equate it with the first neural of other turtles, but rather to distinguish it from the most anterior neural element of other turtles, which she re-labelled as the second neural, as they are associated with the second dorsal vertebra. So, while there is no agreement if the preneural originates from the periosteum of the first dorsal vertebra, it is clear that the preneural is an additional structure relative to most other turtles. More or less convincing "preneurals" have otherwise been identified among early stem turtles (Szczygielski & Sulej, 2019), baenids (e.g., Gilmore, 1919; Hay, 1906), crown cheloniids (e.g., Zangerl, 1969), sinemydids (e.g., Brinkman & Peng, 1993; Tong et al., 2004), carettochelyids (e.g., Joyce, 2014), and meiolaniforms (e.g., Sterli et al., 2015), but I suggest their homology be reviewed independently from what I, here, conclude for pan-trionychids. The preneural found in the shell of many marine turtles (Gentry, 2018; Zangerl, 1953) has recently been confirmed to be situated above the first thoracic vertebra, but not connected to is. The term preneural thus appears appropriate here as well (Menon & Joyce, 2025).

### Comparative basis

It is not the goal of this study to establish the phylogeny of extant trionychid turtles using morphological characters, as this has already been accomplished with high confidence using molecular data (e.g., Engstrom et al., 2004; Le et al., 2014; Thomson et al., 2021). Instead, I here am developing an expanded set of morphological shell characters and investigating which parsimony settings yield the most accurate results by comparison to a molecular tree, with the aim of better resolving the fossil record of the group. As the available molecular trees lack complete sampling and vary slightly in their topology, this demands manual creation of a molecular consensus tree.

**Fig. 15:**
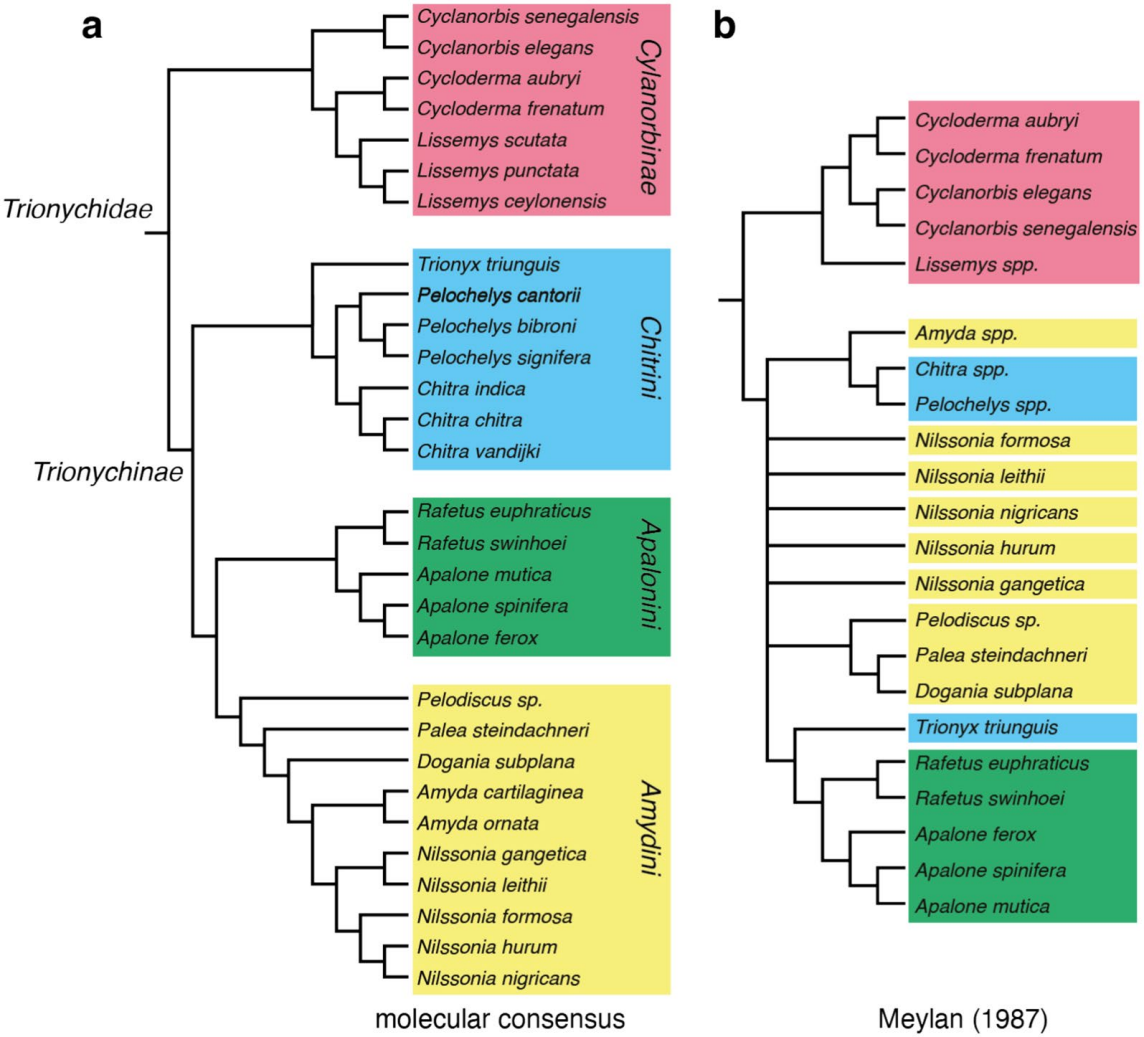
**a** The molecular consensus tree used to assess the quality of the new set of characters, coding schemes, parsimony settings. **b** A strict consensus of the two most parsimonious trees provided by Meylan (1987)

The topology I here utilize as a comparative basis primarily follows those of Engstrom et al. (2004) and Thomson et al. (2021). As the latter analysis is based on more data and newer search algorithms, the two disagreements between the two trees are resolved in favor of the topology of Thomson et al. (2021). As neither Engstrom et al. (2004) nor Thomson et al. (2021) include all three recognized *Lissemys* and *Nilssonia* species, these taxa are internally resolved following Le et al. (2014). I am not aware of any hypothesis for the placement of *Pelochelys signifera*, but its natural range on New Guinea just north of the range of *Pelochelys bibroni* (TTWG, 2021) suggests a sister-group relationship of these two taxa. My manually assembled molecular consensus tree is provided in Fig. 15a and is referred to throughout as the “molecular consensus.”

### Phylogenetic analyses

**Fig. 16:**
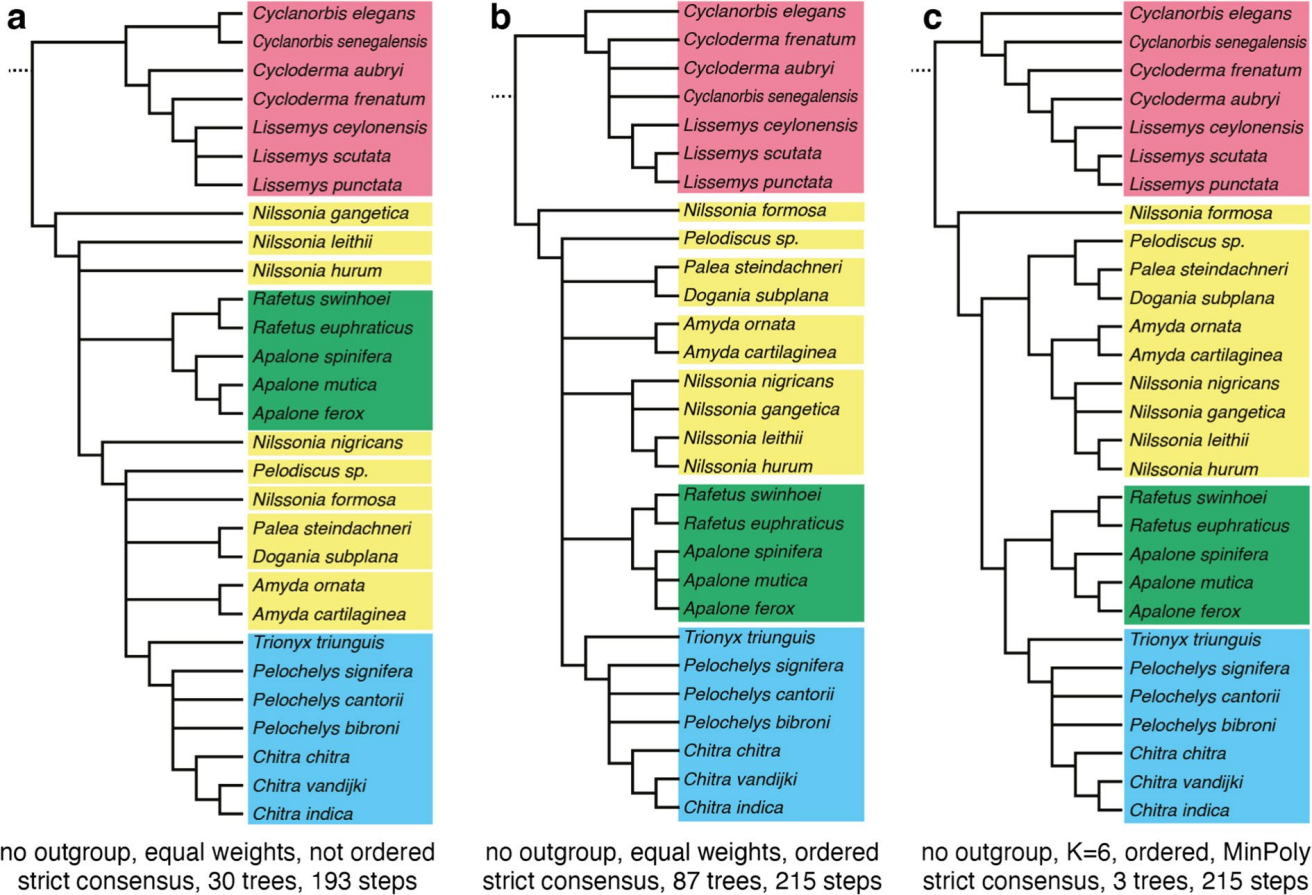
**a–c** strict consensus trees resulting from the analysis of the present character/taxon matrix. The most important settings and results are listed below each tree. The color scheme follows the taxonomy of the molecular consensus (see Fig. 15a)

Character ordering: There is perennial debate in the phylogenetic literature if multistate characters that form morphoclines should be ordered or not (e.g., Brocklehurst & Haridy, 2021; Grand et al., 1993; Slowinski, 1993). To explore the impact of ordering characters upon the present character taxon matrix, I ran two initial analysis using the regular set of terminal taxa, no outgroup, and equal weights, that differ only in the ordering of those characters that form morphoclines (i.e., characters 1, 3, 5, 6, 8, 10, 11, 17, 18, 21, 22, 24, 26, 31, 34, 35, 39, 41–44, 47, 48, 51–54, 56, 58–62, 65, 66, 68). The unordered analysis resulted in 30 most parsimonious trees (MPTs) with 193 steps. These trees, on average, correctly resolve 65% of *Strict Joint Assertions* (*sja*), the value that captures the percentage of resolved quartets in the MPTs that are identically resolved in the molecular reference tree (Table 2). The ordered analysis, by contrast, resulted in 87 MPTs with 215 steps (Fig. 16b), which, on average, yield *sja* values of 73% (Table 2). This demonstrates that more accurate results are obtained from this matrix, if its characters are run ordered.

**Table 2:** Key values resulting from the comparison of trees generated from the present dataset using the R package Quartet (Smith, 2019)

Outgroups	Settings	Figure	Q	x̄s	x̄d	x̄*sja* (%)
Manually rooted	EW, unordered	16a	23,751	15,507	8244	65
Manually rooted	EW, ordered	16b	23,751	17,310	6441	73
Manually rooted	K = 6, ordered	16c	23,751	19,126	4625	81
*P. lamadongensis*	K = 6, ordered	17a	23,751	14,884	8867	70
*C. insculpta* or *A. amtgai*	K = 6, ordered	17b	23,751	16,460	7292	68
MaxPoly manually rooted	K = 6, ordered	17d	23,751	16,468	7283	59

x̄d, average number of quartet statements that are resolved differently in the most parsimonious trees relative to the reference tree; EW, Equal weighting; Q, number of quartet statements possible for the number of terminal taxa present in the trees; x̄s, average number of quartet statements that are resolved identically in the most parsimonious trees relative to the reference tree; x̄sja%, average Strict Joint Assertions (=s /[s + d]), i.e., the proportion of correctly resolved quartets relative to the number of possible quartets

Weighting: Implied Weighting has been suggested to have a beneficial effect upon parsimony analyses, but the weighting factor K must be adjusted to any given character taxon matrix (Ezcurra, 2024; Goloboff, 1993; Goloboff et al., 2018). To investigate the effects of implied weighting, I compare the results of three analyses that differ in their weighting: equal (the same as above), K = 6, and K = 3. Low K-values (i.e., strong weighting) were selected, because the matrix is small (Ezcurra, 2024). In all cases, the regular set of taxa was used, no external outgroup was added, and all morphoclines were run ordered (see previous paragraph for justification). The unweighted analysis resulted in 87 MPTs with 215 steps. The two weighted analyses, by contrast, resulted in 3 trees with a best score of about 12.99 and 20.67, respectively, and a tree length of 215. As the trees are identical in either case of weighting (see Fig. 16c for strict consensus), I only further discuss the results of the analyses using a lighter weighting factor (i.e., K = 6). The average *sja* values of the equal and weighted analyses is 73% versus 81% relative to the molecular consensus, respectively (see Table 2). It is therefore apparent that the use of implied weights yields more accurate results. I can recommend use of implied weighting with K = 6 for the present analysis, but make no recommendation for future analyses that expand upon the present dataset through the inclusion of fossils, as the weighting factor should be adjusted to the size of the matrix (Ezcurra, 2024; Goloboff et al., 2018).

Outgroups and problems with Perochelys lamadongensis: The character/taxon matrix of Meylan (1987) was developed prior to the prevailing use of computer aided analyses. All characters were therefore polarized a priori by reference to outgroups and the trees computed manually (Meylan, 1987). As the results of Meylan’s (1987) work greatly differ from the molecular results of Engstrom et al. (2004) (see Fig. 15a, b), some initial parsimony analyses that are based on this matrix and include fossils used a molecular backbone that allows for fossils to float relative to all extant taxa (e.g., Joyce & Lyson, 2010, 2011; Joyce et al., 2009, 2018; Li et al., 2015). This approach somewhat obviates the need for outgroups, as the molecular backbone provides the structure of the tree. This solution is not tenable in long term, however, as use of a molecular backbone alone cannot clarify if ancient taxa are correctly placed inside or outside of the crown.

An ideal outgroup should provide accurate polarity information, while not negatively affecting the topology. As previous, unpublished analyses undertaken by myself over the course of the year suggest a negative impact of outgroups upon topology, I ran four analyses using ordered characters and a weighting factor of K = 6 (see justifications above). The first analysis used no external outgroup and was rooted manually (same as weighted analysis in previous section). The three remaining analyses varied in the use of the Early Cretaceous pan-trionychid *Perochelys lamadongensis* Li et al., 2015 (as described by Li et al., 2015), the extant carettochelyid *Carettochelys insculpta* Ramsay, 1887, and the Late Cretaceous adocid *Adocus amtgai* Narmandakh, 1985 (as described by Syromyatnikova et al., 2013) as an outgroup. The first taxon was chosen, as it is the oldest known pan-trionychid with a fully documented shell and a plausible stem trionychid. *Carettochelys insculpta* was chosen as it is the extant sister group of trionychids. And *Adocus amtgai* was selected, because it is a well-preserved aquatic representative of the clade *Adocusia*, which is commonly hypothesized to be the closest fossil outgroup of the extant clade *Trionychia* (e.g., Danilov & Parham, 2006; Joyce et al., 2021).

**Fig. 17:**
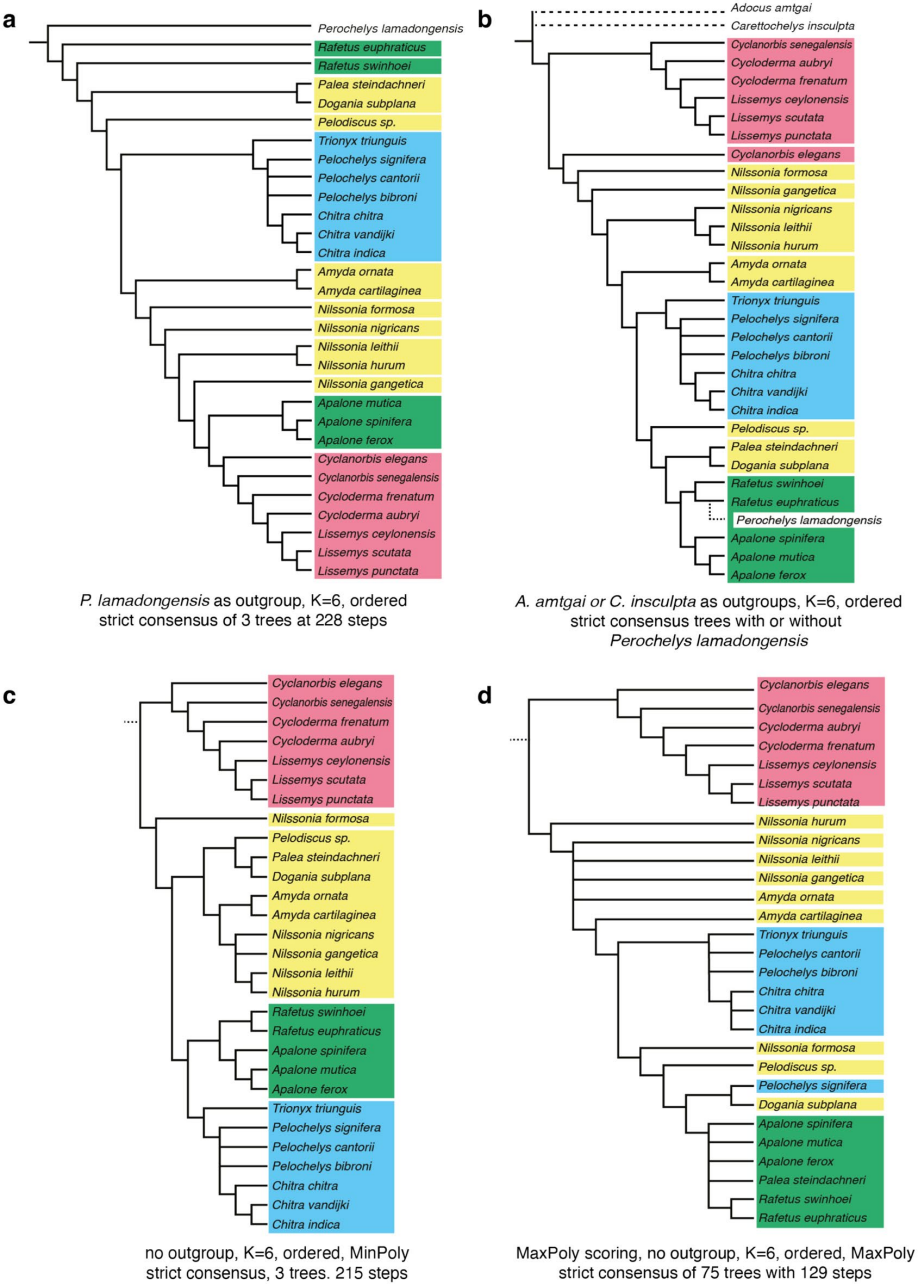
**a–d** strict consensus trees resulting from further analysis of the present character/taxon matrix. The most important settings and results are listed below each tree. The color scheme follows the taxonomy of the molecular consensus (see Fig. 15a)

The manually rooted analysis resulted in 3 most parsimonious trees (MPTs) that imply 215 steps (Fig. 16c). The analyses rooted using *Perochelys lamadongensis* yielded 3 MPTs that imply 228 steps (Fig. 17a) and the two analyses using *Carettochelys insculpta* and *Adocus amtgai* each yielded the same 6 trees, which imply 233 versus 238 steps, respectively (Fig. 17b). Additional analyses with *Carettochelys insculpta* and *Adocus amtgai* that include *Perochelys lamadongensis* as an additional ingroup taxon place the latter deep within the tree as sister to *Rafetus euphraticus* (Fig. 17b). Interestingly, while the ingroup portion of the manually rooted tree has an average *sja* value of 81% relative to the molecular consensus, that of the analyses using *Perochelys lamadongensis* has an average *sja* value of 70% and those using *Carettochelys insculpta* or *Adocus amtgai* is 70% have an average *sja* value of 68% (Table 2), suggesting a negative effect upon topology from the inclusion of fossil outgroups. This is surprising given the strong consilience otherwise found between the morphological and molecular signal of extant trionychids (see previous sections). In particular, the analysis using *Perochelys lamadongensis* as the outgroup is able to retrieve a monophyletic *Cyclanorbinae* and *Chitrini*, but *Apalonini* and *Amydini* are rendered deeply polyphyletic and at the base of the tree (Fig. 17a). The analyses using *Carettochelys insculpta* and *Adocus amtgai*, by contrast, retrieve a monophyletic *Apalonini* and *Chitrini*, but *Cyclanorbinae* is rendered paraphyletic and *Amydini* polyphyletic (17b).

While it is clear that all selected outgroups deeply affect the topology of the tree and imply evolutionary histories different from the molecular result, the tree rooted using *Perochelys lamadongensis* appears to be particularly problematic, as cyclanorbines are found nested deeply within trionychines. This appears to be the direct result of numerous juvenile (neotenic) characteristics, particularly the poorly ossified plastron with small or missing callosities, shared between *Perochelys lamadongensis* and extant *Rafetus* spp., which pull the latter to the base of the tree to unite all more heavily ossified (peramorphic) morphotypes. I see two possible causes to this problem. First, it is possible that *Perochelys lamadongensis* is not a neotenic taxon, but rather a true juvenile not representative of its species. This possibility is somewhat negated by the neotenic appearance (Kordikova, 2000) of most other Early Cretaceous pan-trionychids, in particular *Perochelys hengshanensis* (Brinkman et al., 2017) and *Petrochelys kyrgyzensis* (Nessov, 1995). Brinkman et al. (2017) recently suggested these turtles to be adults, as their costal callosities are well-developed, but my observations as part of this study indicate that their metric (i.e., the relative development of the costal callosities) is not applicable across the tree, which, of course, does not indicate their conclusion to be incorrect. The second possibility is that *Perochelys lamadongensis* faithfully represent the morphology of early trionychids (Brinkman et al., 2017; Kordikova, 2000) but that character evolution was more complicated at the base of the tree than suggested by living trionychids alone. A possible scenario is that poorly ossified, neotenic morphotypes were favorable during the early evolution of the group, but that multiple lineages converged upon more heavily ossified, peramorphic morphotypes in later stages of evolution, perhaps in response to the evolution of small bodied, predatory mammals. This scenario is supported by the demonstrably independent evolution of at least two heavily ossified plastomenid lineages around the K/Pg boundary (e.g., Girard et al., 2024) and the independent rise of predatory defense strategies in other lineages of turtles around the same time (Evers et al., 2025; Lyson & Joyce, 2009).

Li et al. (2015) already noted that resolving the phylogenetic position of *Perochelys lamadongensis* was difficult, as this taxon was found in their analyses as sister to various extant species, such as *Pelodiscus sinensis*, but not at the base of the tree, as would be expected from the oldest then known pan-trionychid. This problem is herein further highlighted by the two analyses using non-trionychid outgroups, which retrieve *Perochelys lamadongensis* as sister to *Rafetus euphraticus*. This appears highly implausible, as it predicts that the extant *Rafetus* diverged in the Early Cretaceous. This may be in part due to the fact that the chosen outgroups (*Carettochelys insculpta* and *Adocus amtgai*) have heavily ossified shells, which gives them a peramorphic appearance relative to *Perochelys lamadongensis* and most trionychines. The prediction that the heavily ossified cyclanorbine morphotype is the ancestral crown trionychid condition, however, is contradicted by the observation that trionychids with unambiguous cyclanorbine affiliations are not known from the Mesozoic (Brinkman et al., 2017; Joyce et al., 2013).

Brinkman et al. (2017) addressed the issues outlined above for their matrix through the use of implied weights in combination with a molecular backbone. An exploratory analysis (not shown) indicates that these steps are not sufficient for the novel data matrix presented herein, as *Perochelys lamadongensis* is still retrieved as sister to *Rafetus euphraticus*. In the short-term, this problem may perhaps be overcome through the use of Bayesian methods, which take stratigraphy into account, as recently implemented by Evers et al. (2023) and Girard et al. (2024). In the long-term, this issue can hopefully be resolved through the inclusion of fossils, as had been done by Brinkman et al. (2017).

Polymorphism: The present matrix reduces the amount of observed polymorphism by scoring ontogenetically controlled characters by reference to the most adult available specimens and by otherwise ignoring polymorphism below a frequency of 20% (see Materials and Methods above). To explore the impact of this coding strategy, I created a second set of taxa that utilizes all polymorphism observed among the individuals of each species (MaxPoly). The MaxPoly scoring was then compared to the regular scoring with reduced polymorphism (MinPoly) in two analyses that otherwise overlap in the use of ordered characters, lack of an outgroup, and a weighting factor of K = 6. The MinPoly analysis (as same as in the previous sections) yielded 3 trees with a best score of about 12.99 and a tree length of 215 (Figs. 16c, 17c). The MaxPoly dataset, by contrast, resulted in 75 trees with a best score of about 5.17 and 129 steps (Fig. 17d). While the strict consensus of the MinPoly analysis has an accuracy of 81%, the MaxPoly analysis shows a reduced accuracy of 59% (Table 2). The MaxPoly dataset thus performed far below the MinPoly dataset.

The “list changes” function of TNT allows exploring the causes of this difference. In the MinPoly analysis, 5 of the 69 included characters (i.e., characters 4, 15, 27, 36, 63, using regular numbering) are listed as parsimony uninformative (i.e., have 0 character state changes). In all cases, these are characters that were included in the list because they address variation seen among fossil pan-trionychids. For the MaxPoly analysis, by contrast, 13 characters are listed as parsimony uninformative (i.e., characters 4, 10, 12, 14, 15, 25, 27, 29, 30, 36, 61, 62, 63, using regular numbering). The reduction of polymorphism thus proximally leads to the creation of informative characters that yield accurate phylogenetic information that would have otherwise been disregarded.

The MinPoly and MaxPoly analyses differ strongly by the number of steps they imply, 215 versus 129. The “list changes” function of TNT demonstrates that the 86 additional steps are relatively evenly distributed across about two thirds of characters. The reduction of polymorphism thus also secondarily led to the recognition of additional transitions that document meaningful phylogenetic information that would otherwise have been disregarded.

The present analysis primarily reduced polymorphism using two methods. The first method scores characters that show ontogenetic variation by reference to the most skeletally mature specimens. This approach had not previously been implemented for turtles to this extreme, in part, I suspect, as most other groups of turtles do not show as much variation as trionychids in later stages of growth, but the results of this study certainly suggest that other analyses might profit from similar methods (i.e., the partial to full dismissal of information obtained from juveniles). Of course, there is no reason to dismiss the possibility that juvenile stages by themselves yield useful phylogenetic data, as has been shown for numerous groups of arthropods using Hennig’s (1966) concept (e.g., Sharma et al., 2017). However, as the fossil record of turtles is relatively depauperated of identifiable juveniles, the development of characters focused on juvenile characteristics is unlikely to yield meaningful results, in part also as most juveniles found in the fossil record cannot be attributed with confident to any particular taxon.

The present analysis furthermore reduced polymorphism by implementing a 20% filter, both for characters controlled, or not controlled by ontogeny. This method was chosen over a frequency method for several reasons. First, as sample sizes are relatively low for turtles, especially fossils, it is unreasonable to expect that observed frequencies are accurate. Second, the application of a 20% threshold yields discrete character states and discrete character state changes, which are easy to implement using standard phylogenetic software. And third, and perhaps most importantly, the dismissal of characteristics found in less than 20% of individuals allows focusing on the “normal” morphology of a taxon, instead of morphologies caused by the noise-like phenotypic intraspecific variation so typic of trionychids. This approach may be useful for other groups of turtles as well known to show particularly high levels of polymorphism, particularly various groups of testudinoids (Garbin et al., 2018; Vlachos & Rabi, 2018).

### Osteological characterization of extant clades and species

An accessory realization of this study is that the shell osteology of extant trionychids is poorly characterized, resulting in the misidentification of many specimens held in museum collections. I, therefore, am taking the liberty to provide a brief characterization of all currently recognized species with focus on those recently established using molecular data. The list of diagnostic characters was mostly guided by mapping the herein established characters onto the molecular consensus tree (see above).

*Amydini*: The present analysis finds no apomorphic characters in support of the clade *Amydini*, but they can be diagnosed relative to *Apalonini*, *Chitrini*, and *Cyclanorbinae* by the apomorphic characters they lack (see relevant sections below). As the internal nodes are poorly supported as well, I focus on the diagnoses of the currently recognized genera and species.

*Amyda: Amyda* is diagnosed by the presence of an anteroposteriorly deep nuchal callosity (character 11, state 2) which correlates with development of a distinct anteromedial nuchal callosity protrusion (character 13, state 1). They exhibit, like *Nilssonia* spp., notably long anterior epiplastral processes (character 54, state 2).

The current literature recognizes two species of *Amyda*, *Amyda cartilaginea*, which occurs south of the Malay Peninsula, and *Amyda ornata*, which occurs north of the Malay Peninsula (TTWG, 2021), but a study using more extensive molecular data suggests presence of up to six lineages with differing coloration and texture patterns but only relatively shallow divergences (Fritz et al., 2014). When partially overlapping scorings are excluded (e.g., character 26 where *Amyda cartilaginea* scores 6, while *Amyda ornata* scores 5&6), only two differences are apparent for the two currently recognized species: character 1, midline length of carapacial disk (*Amyda cartilaginea* scores 4 [carapace disk length 40–50 cm], while *Amyda ornata* scores 5 [carapace disk length 50–60 cm]) and character 44, the anterior development of the hyoplastral callosity (*Amyda cartilaginea* scores 2 [hyoplastral callosity anteriorly expands into the axillary notch], while *Amyda ornata* scores 1 [hyoplastral callosity restricted to the hyoplastral processes]). The development of an anteriorly expanded hyoplastral callosity in *Amyda cartilaginea* represents a peramorphosis for this species relative to *Amyda ornata* (sensu Alberch et al., 1979), as this characteristic is ontogenetically controlled (see Character 44 above), but *Amyda cartilaginea* is the smaller of the two species. The two *Amyda* species are therefore not fully cryptic from the perspective of shell osteology. The observed differences are nonetheless insufficient to allow diagnosing most individuals, as they are only observed in the very largest specimens.

Dogania subplana: Dogania subplana is diagnosed by characters unique among extant trionychids, in particular the presence of carapacial kinesis (character 7, state 1), a continuous series of neurals that fully subdivide the costals (character 32, state 9), and highly reduced hyo/hypoplastral callosity lacking any lateral extension (character 47, state 2). A number of homoplastic characters are further characteristic for this species: a particularly short nuchal callosity (character 11, state 0), presence of medially confluent suprascapular fontanelles (character 16, state 2) that never close in ontogeny (character 17, state 3), placement of paired pits for articulation with the neck in the posterior third of the nuchal (character 22, state 0), consistent lack of free rib ends (character 37, state 2), development of narrow dorsal ribs (character 38, state 1), lacking plastral sculpturing or plastral sculpturing consisting of raised tubercles (character 45, 0&2), and a particularly narrow angle between the facing lateral processes of the hyo- and hypoplastron (character 62, state 0).

*Nilssonia*: No unique or particularly meaningful characters unite the five currently recognized species of *Nilssonia*, but they can be diagnosed by the presence of particularly long anterior epiplastral processes (character 54, state 2), which are also seen in *Amyda* spp., and the presence of a preneural (character 23, state 0, versus 1), which, however, is secondarily absent in *Nilssonia formosa*. *Nilssonia* spp. are notable for often displaying a particularly coarse netted shell surface texture. Although the matrix shows much variation among the five named species, only few characters are consistent for the shell, which is why it is difficult to diagnose *Nilssonia* specimens based on a shell alone. The following characteristics stand out: *Nilssonia formosa* is unique among *Nilssonia* species by lacking a preneural (character 23, state 0, versus 1), consistently lacks an entoplastral callosity (character 42, state 0, versus 1), and often exhibits fused hyo/hypoplastral callosity (character 46, state 1). In adults of *Nilssonia gangeticus*, costal I embraces more than one half of the nuchal laterally. *Nilssonia nigricans* has particularly large costals VIII (character 34, state 2, versus 1). *Nilssonia leithii* possesses particularly small xiphiplastral callosities that only partially cover the xiphiplastral processes (character 51, state 1, versus 2&3). As difficult as it is to diagnose *Nilssonia* species based on their shell, their skulls are highly distinct (Gray, 1864).

Palea steindachneri: Palea steindachneri is a small sized amydine that greatly resembles *Pelodiscus* spp. Some individuals display a distinct carapacial surface texture consisting of anteroposteriorly elongate pustules (e.g., MTD 33802), but this texture is typically lacking. In contrast to *Pelodiscus* spp., *Palea steindachneri* typically lacks reduced eighth costal ribs (character 39, state 0, not 1), displays a plastron textured by raised pustules, not netting (character 45, state 0, not 1), has reduced hyo/hypoplastral callosities that do not protrude beyond the hyo-hypoplastral processes anteriorly (character 42, state 1, not 2) or medially (character 48, state 0&1, not 2), has small xiphiplastral callosities that barely cover the processes (character 51, state 1, not 2), exhibits a narrower angle between the lateral processes of the entoplastron (character 58, state 1, not 2), and only slightly expanded lateral processes of the entoplastron (character 59, state 1, not 2).

*Pelodiscus*: A number of features are highlighted above that distinguish *Pelodiscus* spp. from *Palea steindachneri*, the only other unspecialized and small sized amydine.

Six species of *Pelodiscus* are currently accepted (TTWG, 2021), but the available dataset is insufficient to explore interspecific variation, as most specimens cannot be reliably referred to a particular taxon. Given the relatively deep divergence of the group (Le et al., 2014), however, I expect future analyses to reveal many osteological differences.

Apalonini: Extant apalonines are united by two synapomorphies, the closure of suprascapular fontanelles either late in life or never (character 16, states 2&3), which occurs homoplastically in small sized amydines, and the strong reduction of costal rib VIII (character 39, state 2), which otherwise occurs in *Trionyx triunguis* as well. The reduction of the costal rib VIII correlates strongly with the reduction to loss of costal VIII (character 34, states 0&1), which, however, does not serve as a synapomorphy to the group, as it is scored polymorphically across much of the tree.

*Apalone*: All three species of *Apalone* can easily be recognized among trionychines by the development of single lateral hyoplastral processes (character 60, state 1), a feature that otherwise only occurs in cyclanorbines, though associated with a different plastral arrangement. The clade *Apalone* is otherwise supported by the expansion of the hypoplastral callosity to cover the medial hypoplastral process or even fused at the midline (character 48, states 2&3) and the abutting to sutured contact of the hypoplastral and xiphiplastral callosities (character 60, states 1&2), but these characters, too, occur homoplastically across the tree.

*Apalone mutica* is the smallest of the three species, with a carapace disk length typically well below 18 cm. Its shell is unique among extant trionychids by having particularly short anterior epiplastral processes, a character not used in the present analysis, and by having a thick lateral hyoplastral process that is almost oriented anteriorly due to a particularly wide angle with the lateral hypoplastral processes (character 62, state 2). The first costal otherwise surrounds the posterior half of the nuchal (character 31, state 1), small epi- and entoplastral callosities are often present (characters 40 and 42, state 1), and the angle between the lateral entoplastral processes are particularly wide (character 58, state 2). The netted to pitted shell texture is fine.

*Apalone ferox* is the largest *Apalone*, with a carapace disk length often above 25 cm. It can easily be distinguished from other *Apalone* by having a particularly wide nuchal (character 10, state 2), split costals (character 30, state 1), a more ossified plastron, which sometimes includes an anteriorly protruding hyoplastral lappet (character 42, state 3), suturally connected hyo-, hypo, and xiphiplastra (character 48, state 3; character 51, state 3; character 52, state 2), and fusion of the hyo/hypoplastral callosity (character 46, state 1). Its surface texture is always netted and notably coarse.

*Apalone spinifera* is intermediate in size (a carapace disk length typically around 20 cm) and notable for lacking unique apomorphies beyond the common presence of an unsculptured lateral rim on the carapace (character 6, state 0&1) and octagonal neurals (character 28, state 1).

*Rafetus*: The two extant species of *Rafetus* are united by three synapomorphies, all of which related to their highly reduced plastron, in particular hyo/hypoplastral callosities that lack contacts with all surrounding elements (character 42, state 1; character 48, state 0), which occurs homoplastically in *Dogania subplana* and *Palea steindachneri*, and the complete lack of xiphiplastral callosities (character 51, state 0), which otherwise only occurs in *Cyclanorbis elegans*. The carapace typically exhibits a narrow, unsculptured margin (character 6, state 0&1), a rounded, particularly deep nuchal (character 11, state 1&2), and relatively broad anterior neurals, a feature not otherwise developed herein.

*Rafetus euphraticus* is much smaller than *Rafetus swinhoei*. The most notable differences that diagnose *Rafetus euphraticus* are suprascapular fontanelles that remain open throughout life (character 17, state 2; versus state 3 for *Rafetus swinhoei*) and highly reduced hyo-hypoplastral callosities that do not expand laterally to cover the lateral processes (character 47, state 0; versus state 1 for *Rafetus swinhoei*) or posteriorly to cover the inguinal notch (character 49, state 0; versus state 1 for *Rafetus swinhoei*).

*Chitrini*: All representatives of *Chitrini* are large bodied turtles (character 1, states 4 and higher) that exhibit two synapomorphies: the development of widely spaced epiplastra (character 56, state 0), homoplastically present in cyclanorbines, and formation of an expanded medial hypoplastral comb (character 66, states 0&1). Within this clade, *Chitra* and *Pelochelys* can most easily be distinguished from *Trionyx triunguis* by having wide neurals (character 28, state1), a narrow angle of the lateral entoplastral processes (character 58, state 0), and particularly expansive medial hyoplastral (character 65, state 0), medial hypoplastral (character 66, state 0), and medial xiphiplastral processes (character 68, state 3).

*Chitra: Chitra* is highly apomorphic relative to all other trionychids by closing their suprascapular fontanelles early in life (character 19, state 0), a feature also seen in cyclanorbines, and by uniquely having a nuchal with a rib-like lateral nuchal process (character 19, state 0), paired depressions in the anterior third of the nuchal for the cervical articulation (state 20, state 2), and three lateral hypoplastral processes (character 61, state 3).

What was formerly *Chitra indica* is now divided into *Chitra indica*, *Chitra vandijki*, and *Chitra chitra*, which occur west, within, and east of the Irrawaddy River System of southern Asia (TTWG, 2021). As the present analysis only includes a single, subadult specimen of *Chitra vandijki* that is unlikely to be fully representative of its species, I am focusing on difference between the other two. Ignoring partially overlapping scorings, only two differences are apparent from the matrix: character 1, median shell length (*Chitra chitra* scores 7 [carapace disk length 70–80 cm], while *Chitra indica* scores 5 [carapace disk length 50–60 cm]) and character 31, costal I extension lateral to the nuchal (*Chitra chitra* scores 1 [costal I laterally surrounded posterior half of nuchal], while *Chitra indica* scores 0 [costal I located posterior to nuchal]). Given that the development of character 31 correlates with size (see Character 31 above), the two differences are interrelated and may represent a sampling artifact. Although scorings overlap, the larger *Chitra chitra* tends to have a broader nuchal callosity (character 10, state 2, versus states 1&2 in *C. indica*), wider costal VIII (character 35, states 2&3, versus state 2 in *C. indica*), and a broader angle between the entoplastral processes (character 58, states 0&1, versus state 0 in *C. indica*), but these differences might be obliviated by further sampling as these characters are size controlled as well. However, the smaller *Chitra indica* tends to have larger xiphiplastral callosities (character 51, states 1&2, versus state 1 for *C. chitra*) with a broader midline contact (character 53, states 0&1, versus state 0 for *C. chitra*), two features that are peramorphic relative to *Chitra chitra*, while *C. chitra* tends to form a poorly- to well-developed third lateral hyoplastral process (character 60, states 0&1, versus 0 for *C. indica*), a character not controlled by size (see Character 60 above). I therefore conclude that there is at least some morphological support for the recognition of *Chitra chitra* and *Chitra indica* as distinct species, but note that the observed differences are relatively nuanced and mostly developed in the largest available specimens only.

*Pelochelys: Pelochelys* stands out by exhibiting broadly split costals (character 30, state 1), a feature that otherwise only occurs in *Apalone spinifera*.

The former taxon *Pelochelys bibroni* was split into three species over the course of the last two decades: *Pelochelys cantorii*, which occurs across southeast Asia, *Pelochelys bibroni*, which occurs across southern New Guinea, and *Pelochelys signifera*, which occurs across northern New Guinea (TTWG, 2021). As I only have access to a single specimen of *Pelochelys signifera* that is unlikely to be representative of its species, I focus on differences between the other two. These two species can only be differentiated by relatively nuanced differences to the development of the contact between the xiphiplastral and hypoplastral callosities, which is absent in the available specimens of *Pelochelys bibroni* (character 52, state 0), but blunt in *Pelochelys cantorii* (character 52, state 1). *Pelochelys bibroni* otherwise tends to have a more anteriorly located nuchal depression for the cervical joint (character 22, state 0&1; versus state 0 for *Pelochelys cantorii*), more reduced costal ribs VIII (character 39, state sate 0&1; versus state 0 for *Pelochelys cantorii*), and a denser medial hyoplastral comb (character 65, state sate 0&1; versus state 0 for *Pelochelys cantorii*), while *Pelochelys cantorii* tends to have proportionally broader costal VIII (character 35, state 2&3; versus state 2 for *Pelochelys bibroni*) and somewhat broader lateral entoplastral processes (character 59, state 0&1; versus state 0 for *Pelochelys bibroni*). I find these differences to be particularly nuanced. This, in return, somewhat questions the recognition of three distinct species.

*Trionyx triunguis:* The analysis shows no autapomorphies for *Trionyx triunguis*, but numerous characters diagnose it as part of *Chitrini* and distinguish it from other representatives of the group (see *Chitrini* above).

*Cyclanorbinae:* The clade Cyclanorbinae is supported by five unambiguous characters: the minor to strong underlap of costal I by the nuchal processes (character 21, states 1&2), the common absence of a neural reversals (character 25, state 0), partial to full roofing of the ilia by the carapace (character 33, states 1&2), formation of additional posterior hypoplastral processes that laterally embraces the anterior xiphiplastral processes (character 67, 1), and the posterior orientation of the posterior xiphiplastral processes (character 69, state 1). A number of additional characteristics diagnose this group, but occur homoplastically among select trionychines as well: presence of preneural (character 23, state 1), the development of large costal VIII (character 38, state 2&3), fusion of the hyo/hypoplastra callosity (character 46, state 1), covering of the posterior margin of the lateral hypoplastral processes by the hypoplastral callosity (character 50, state 1), and widely spaced epiplastra (character 56, state 0), which is homoplastic with some chitrines. The clade consisting of *Cycloderma* and *Lissemys* is supported by the development of a posteriorly constricted carapace (character 2, state 1), presence of a laterally split nuchal comb (character 19, state 2), absence of posterior epiplastral processes (character 55, state 0), and an entoplastron with a broad anterior shelf (character 57, state 3).

*Cyclanorbis:* The clade *Cyclanorbis* is supported by a single, but unique character among extant trionychids, which is the loss of all medial xiphiplastral processes (character 68, state 0). *Cyclanorbis elegans* can be recognized by only having a partially roofed ilium (character 34, state 1) and complete absence of a xiphiplastral callosity, which also is lacking in *Rafetus*. *Cyclanorbis senegalensis*, by contrast, is a particularly autapomorphic taxon, diagnosed by four apomorphies unique among extant trionychids: lateral expansion of the nuchal callosity relative to the nuchal processes (character 14, state 1), reduction to the neural count (character 24, states 5&6), development of neomorphic plastral callosities (character 40, state 1), and development of a clear, blunt contact of the entoplastral callosity with the hyoplastral callosity (character 47, state 1). In addition, *Cyclanorbis senegalensis* can be diagnosed by presence of a prenuchal (character 8, state 1, homoplastically found in *Lissemys*) and formation of an expanded medial shelf by the hyo/hypoplastral callosity (character 48, state 2, homoplastically found in *Cycloderma aubryi*).

*Cycloderma:* The monophyly of *Cycloderma* is not supported by a single meaningful osteological shell character. *Cycloderma aubryi* can be differentiated from *Cycloderma frenatum* by lack of a protrusion of the nuchal callosity along the full length of the nuchal (character 13, state 0, versus 2), expansion of the hyo/hypoplastral callosity to the dorsal side of the shell (character 47, state 3, versus 1), development of a much more extensive medial extension of the hyo/hypoplastral callosity (character 48, 3, versus 1), notably wide entoplastral processes (character 59, state 2, versus 0&1), only a single lateral hyoplastral process (character 60, state 1, versus 2), a particularly wide angle between the facing lateral hyo- and hypoplastral processes (character 62, state 2, versus 1), and presence of an uneven medial hypoplastral comb (character 66, state 1, versus 2).

*Lissemys:* The taxon *Lissemys* can be diagnosed by the presence of a prenuchal (character 8, state 1, homoplastically found in *Cyclanorbis senegalensis*), presence of neomorphic peripherals (character 9, state 1), development of a deep nuchal notch (character 12, state 1), anterior protrusion of the nuchal callosity along the full length of the nuchal (character 13, state 2, also in *Cycloderma frenatum*), distinctly narrow costal ribs (character 38, state 1), expansion of the hyo/hypoplastral callosity to the dorsal side of the shell (character 47, state 3, also in in *Cycloderma aubryi*), and large xiphiplastral callosities that are sutured along the midline (character 53, also found in some trionychines).

Three species of *Lissemys* are currently recognized: *Lissemys ceylonensis*, which occurs in Sri Lanka, *Lissemys punctata*, which occurs across Bangladesh, India, and Pakistan, and *Lissemys scutata*, which occurs in the Irrawaddy River System, mostly of Myanmar (TTWG, 2021). In contrast to the other species recently established using molecular data, these can be differentiated with relative ease. *Lissemys ceylonensis* differs notably from the others by being larger (character 1, state 3 [carapace disk length 30–40 cm], versus state 2 for the others), which correlated with proportionally larger costal VIII (character 34, state 3; versus 2&3 for the others), a contribution of the hypoplastral callosity to the inguinal notch (character 49, state 1; versus state 0 for the others), and a broader angle between the lateral entoplastral processes (character 58, state 1; versus state 0 for the others). However, it also consistently lacks a neural reversal (character 25, state 0; versus 0&1 for the others) and exhibits only a single lateral hyoplastral process (character 60, state 0; versus 0&1&2 for the others), two characters that are not controlled by size.

*Lissemys punctata* is intermediate in size (character 1, state 2 [carapace disk length 20–30 cm], which is achieved by most individuals), which correlates somewhat with it tending to have a reduced contact between the xiphiplastra and hypoplastral callosities (character 52, states 0&1; versus 1 for the others). It differs notably, however, by tending to have fewer intercostal contacts (character 32, state 7&8, versus state 8 for the others) and tending to have more tightly spaced lateral hypoplastral processes (character 64, states 0&1; versus 1 for the others), features that are not size controlled. Most importantly, the prenuchal of *Lissemys punctata* fully fuses to the carapace during ontogeny (character 8, state 2), a feature that is peramorphic relative to *Lissemys ceylonensis*.

*Lissemys scutata* is the smallest of the three species (character 1, state 2 [carapace disk length 20–30 cm], which is only barely achieved by some individuals). This correlates with a tendency to lack central fading of the carapace texture (character 5, state 0; versus 0&1 for the others) and a narrower nuchal callosity (character 10, state 1; versus 1&2 for the others). Yet, this species consistently exhibits only 7 neurals (character 24, state 7; versus 6&7&8 for the others) but shows much variability in regard to the number of lateral hyoplastral processes (character 60, 1&2&3). Most notable, *Lissemys scutata* tends to have a proportionally larger entoplastral callosity that may bluntly contact the hyo/hypoplastral callosity (character 43, state 0&1; versus 0 for the others). Given the small size of this species, this feature must be viewed as peramorphic.

In conclusion, the three currently recognized species of *Lissemys* can be distinguished with relative ease. I suspect this is caused by a preference of upland habitats, which hinders gene flow along the coasts.

## Conclusions

This study is an attempt at best practices in exploring the shell anatomy of extant soft-shelled turtles in search of an expanded list of characters that might help elucidate the fossil record of the group, which is dominated by shell remains. For this purpose, a list of 69 new or revised characters was established and scored separately for 530 individuals representing all currently recognized extant trionychid species, with exception of *Pelodiscus* spp, which had to be included into a single taxon as most available specimens cannot be attributed to any particular species with confidence. This step was only made possible by the availability of a large collection of pictures that I had assembled from across the globe over the course of the last 20 years with help from the fossil turtle research community (see Acknowledgements) combined with the fact that trionychid shells are relatively flat and simple structures whose internal and external morphology can be captured efficiently through the use of photography. A sample of at least 10 individuals per taxon is probably a prerequisite of any future analyses wishing to document polymorphism in any other group of living turtles, but will be difficult to obtain using photographs alone, as the shells of other groups of turtles are typically highly domed structures whose internal and external anatomy cannot be captured readily using snap shots. In that regard, new imaging technologies might provide new impulses, particularly CT scans, as they allow obtaining detailed 3D models, not only of osteological specimens, but also specimens preserved in alcohol. For the moment, however, scanning and processing times are still prohibitive and CT scanners unevenly distributed across the globe.

This study firmly demonstrates that polymorphism is pervasive in the shell of trionychids, but should not be considered problematic a priori. Indeed, although characters were defined herein as precisely as possible, scoring individuals with intermediate morphologies as polymorphic actually eases the process of scoring, especially when a large sample is utilized, as every single observation is downweighed relative to the final result. Similarly, in the case of multistate characters, which dominate the present dataset, the use of polymorphism is a way to gain information as scoring specimens or taxa polymorphic is still more informative that no character information at all.

This study also firmly demonstrates that much polymorphism is caused in trionychids by ontogenetic changes apparent among subadult to adult individuals. Indeed, trionychids seem to be unique among turtles by significantly altering their morphology even late in ontogeny. All together these observations suggest that fossil taxa need to be established with a full understanding of phenotypic and ontogenetic variation in mind.

This study used four simple techniques to stepwise reduce polymorphism. First, prior to scoring individuals, specimens were disregarded that showed extreme pathologies. Second, while scoring individuals, some characters were corrected for minor pathologies (e.g., split neurals were counted as one neural, not two). Third, for character controlled by polymorphism, species were coded by reference to the most ontogenetically mature individuals. And fourth, the remaining polymorphism was further reduced by disregarding all character states were a frequency of less than 20%. While the impact of the first two steps cannot be assessed, the final two demonstrably add resolution to the tree, mostly by rendering characters informative.

The final identification of characters as being ontogenetically controlled, the choice of a 20% threshold, and final coding of species are unique to this study, but may not be ideal or supported by additional data. It is for this very reason that all primary data was scored at the level of the individual. All observations can thus be verified by reference to the original material, additional specimens can be integrated with ease, and the coding of species updated by reference to an expanded primary dataset or using alternative coding strategies. Though onerous, this transparent and flexible approach should be implemented for other groups of turtles as well.

In contrast to earlier attempts at reconstructing the phylogeny of a group of turtles, this study takes full advantage of the emerging molecular consensus, not by implementing a molecular backbone alone, but rather by varying the parsimony setting in search of the best fit relative to the molecular tree. This approach clearly demonstrates that the present dataset should be run with all morphoclines run ordered, but also that the lacking overlap with the molecular tree still calls for the additional use of a molecular backbone.

Although improved sampling, an improved character list, and improved methodologies yielded a result far superior to previous attempts, the present analysis still cannot replicate the emerging molecular consensus completely. A notable problem is the inability of the analysis to retrieve *Amydini* as monophyletic. I am skeptical that further sampling of individuals to better document variability will be able to address this problem, as amydines already are well sampled. And given my systematic approach to the present analysis, I am also skeptical that additional shell character will be found that unite this group. This does not bode well for fully resolving the evolutionary history of the group using fossilized shell material alone, but I remain optimistic that the inclusion of cranial characters and the utilization of stratigraphic and biogeographic data within a Bayesian framework should allow establishing the evolutionary history of the group with far greater precision that currently possible.

## Supplementary Information

Additional file 1. Character-taxon matrix in nexus format, including full list of terminals and individuals, character names, measurements, and other comments.

Additional file 2. Spread sheet utilized to compute Spearman's rank-order correlation for characters 2, 5–8, 10–14, 18, 30, 31, 33–35, 38, 39.

Additional file 3. Spread sheet utilized to compute Spearman's rank-order correlation for characters 41–44, 47–53.

Additional file 4. Spread sheet utilized to compute Spearman's rank-order correlation for characters 54, 56, 58, 59, 62–66, 68.

## Data Availability

All research data is provided in the additional files.
